# Social Determinants of Childhood Vaccination Coverage in the United States Using National Immunization Survey Data From 2010 to 2023: Cross-Sectional Study

**DOI:** 10.2196/81746

**Published:** 2026-04-09

**Authors:** Ramya Keerthi Majji, Yan Zhuang, Felix M Pabon-Rodriguez

**Affiliations:** 1 Department of Biomedical Engineering and Informatics Luddy School of Informatics, Computing, and Engineering Indiana University Indianapolis, IN United States; 2 Department of Biostatistics and Health Data Science School of Medicine Indiana University Indianapolis, IN United States

**Keywords:** childhood immunization, national immunization survey, social determinants of health, survey-weighted regression, vaccination coverage

## Abstract

**Background:**

Vaccination in early childhood is essential to prevent serious infectious diseases and protect community health. In the United States, 7 vaccines (diphtheria, tetanus, and acellular pertussis; polio; measles, mumps, and rubella; *Haemophilus influenzae* type b; hepatitis B; varicella; and pneumococcal conjugate) are recommended by the Centers for Disease Control and Prevention for children aged 19-35 months, along with 3 others (hepatitis A, influenza, and rotavirus), which are recommended for full protection. Despite these guidelines, recent measles outbreaks in the United States have drawn attention to persistent gaps in coverage.

**Objective:**

This cross-sectional study explores immunization patterns among children aged 19-35 months using provider-verified data from the National Immunization Survey-Child between 2010 and 2023.

**Methods:**

Survey-weighted logistic regression models were used to assess associations between vaccination status and social determinants of health, including child age group, maternal education, insurance status, birth order, and region. Separate survey-weighted logistic regression models were fit for each survey year from 2010 to 2023. We considered the complex survey design to calculate vaccination coverage rates and their CIs, accounting for clustering and stratification in the sampling methodology. A composite variable was created to measure full coverage of the 7 recommended vaccines, and within each year, models were estimated separately for each individual vaccine and for the 7-vaccine series composite outcome to identify vaccine-specific barriers to immunization.

**Results:**

Provider-verified response rates declined from 70.8% (17004/24013) in 2010 to 47.7% (18412/38619) in 2023, although the analytic sample size remained stable. Coverage for the Combined 7-Vaccine Series increased from 70.8% (11893/16798) in 2010 to 77.4% (13957/18032) in 2023. Older age was consistently associated with higher odds of complete vaccination (2010: odds ratio [OR] 1.10, 95% CI 1.02-1.19; 2023: OR 1.24, 95% CI 1.13-1.35), whereas lack of insurance and Hispanic origin were associated with lower uptake for selected vaccines. In 2022, uninsured children had 26% lower odds of complete vaccination compared with insured children (OR 0.74, 95% CI 0.65-0.84). Higher income-to-poverty ratio was consistently associated with increased vaccination uptake, including influenza vaccination in 2020 (OR 1.25, 95% CI 1.13-1.39). Regional and language-related disparities persisted across survey years.

**Conclusions:**

Persistent socioeconomic and structural gradients in childhood vaccination coverage highlight the need for equity-focused immunization strategies that address insurance access, language barriers, and geographic disparities.

## Introduction

### Background

Vaccination programs have transformed child health outcomes by lowering the incidence of vaccine-preventable diseases worldwide. These efforts prevent an estimated 2-3 million deaths annually across all age groups, making childhood immunization one of the most effective public health interventions to date [1]. Despite decades of global investment in vaccination infrastructure, gaps remain in immunization coverage, particularly among marginalized communities and for specific vaccines [2]. In the United States, early childhood vaccinations protect against conditions such as measles, pertussis, and polio. Yet, recent resurgence of measles in the United States, along with suboptimal uptake of influenza and hepatitis A vaccines, underscore persistent disparities. These patterns raise concern about whether emerging infectious threats, vaccine hesitancy, and systemic inequities are eroding past gains in population protection [3]. The persistence of undervaccination calls for a deeper understanding of social and structural barriers to vaccine access and trust [4]. Understanding the factors that limit complete immunization is essential for improving equitable vaccine delivery and maintaining high coverage across the population [5].

### Prior Work

Social determinants of health (SDoH) are increasingly recognized as critical drivers of health outcomes, including vaccine access and uptake [6,7]. These determinants include socioeconomic status, maternal education, employment, household size, geographic location, access to health care, and cultural or language barriers [8-10], among others. Financial constraints and limited-service availability are common barriers for low-income families, particularly those without private insurance or who reside in underserved areas. County-level data from the United States have revealed pronounced geographic disparities in immunization, often related to income and education levels [11]. In such settings, tools like small area estimation have been applied to identify pockets of low coverage and guide outreach strategies [11]. These disparities often intersect with race, ethnicity, and immigration status, further limiting vaccination access in vulnerable populations [12]. Together, these interrelated structural and socioeconomic factors shape access to pediatric services and influence the likelihood of full immunization [13,14].

In parallel, vaccine hesitancy, defined as a delay in acceptance or refusal despite availability, remains a serious threat to public health and can exacerbate inequities [15]. Misinformation, perceived safety concerns, and social influences have been identified as key factors in vaccine decision-making [2]. Hesitancy tends to cluster within populations and regions, making it harder to maintain herd immunity [16,17]. When combined with structural disadvantages, these behavioral factors further widen gaps in immunization coverage.

Recognizing and addressing these social and structural factors is central to reducing health inequities. Research incorporating SDoH into public health analysis has demonstrated that targeted interventions, including Medicaid expansion, community-based care investment, culturally tailored communication strategies, and patient navigation services, can improve vaccine uptake [18,19]. Community-driven models that consider cultural norms, transportation access, and flexible scheduling have shown promise in overcoming barriers to care and should be prioritized in public health planning [20]. Partnerships between public health officials, policymakers, researchers, and local organizations are essential to operationalize solutions that account for the full social context in which health decisions occur [21,22]. Integrating SDoH into immunization research not only informs policy decisions but also helps develop more tailored interventions that are likely to succeed across diverse populations [14,23]. Furthermore, vaccination efforts contribute directly to broader Sustainable Development Goals of the United Nations by reducing disease burden, supporting educational attainment, and enabling economic participation [6].

Although prior research has documented associations between social determinants and childhood vaccination coverage, many studies rely on pooled multiyear data, limited time windows, or focus on single vaccine outcomes. Less is known about the temporal stability of these associations across independent survey cycles, particularly before and after the COVID-19 pandemic, and about whether disparities differ systematically across specific vaccine types. Addressing these gaps is essential for understanding whether observed inequities represent persistent structural patterns or time-specific disruptions.

### Study Objective

This study conducts year-specific survey-weighted analyses of National Immunization Survey-Child (NIS-Child) data from 2010 to 2023 to examine the temporal stability, pandemic-related shifts, and vaccine-specific heterogeneity of associations between social determinants and childhood immunization coverage. Focusing on children aged 19-35 months, we assess how maternal education, insurance status, income-to-poverty ratio, language, and geographic region are associated with individual vaccine uptake and completion of the Combined 7-Vaccine Series. These analyses provide updated evidence on disparities in childhood immunization coverage across prepandemic and postpandemic periods.

## Methods

### Data

This cross-sectional study uses data from the NIS-Child, which is publicly available through the Centers for Disease Control and Prevention (CDC) [24,25]. The NIS-Child is an annual, nationally representative survey conducted by the CDC using a random digit–dial sampling design of landline and cellular telephone numbers. Households with children aged 19-35 months are identified through telephone screening interviews. With parental consent, vaccination providers are contacted by mail to obtain official immunization records. Final survey weights adjust for sampling probability, nonresponse, and poststratification to US population benchmarks. The survey produces reliable, population-based estimates at the national, state, and selected local levels using a consistent sampling and verification process. The survey is administered by the University of Chicago under the direction of the CDC [26]. This study used all available NIS-Child data meeting inclusion criteria from 2010 to 2023. No formal sample size calculation was performed as this was a secondary analysis of existing surveillance data. The final analytic sample included all children aged 19-35 months with provider-verified immunization records across the 13-year study period.

### Variables

To assess childhood immunization trends and disparities, we selected key variables grounded in demographic, socioeconomic, and household contexts. These variables align with SDoHs identified in prior literature as influential factors in vaccine uptake. In addition to child characteristics like age group, sex, and birth order, we considered household and maternal variables, such as maternal education, income-to-poverty ratio, and language of interview, to capture broader influences on immunization behavior.

For every year from 2010 to 2023, we derived new binary coverage variables indicating whether a child received the minimum required doses based on provider-verified data (adequate provider data identifier [PDAT]). These thresholds were based on the CDC’s standard immunization schedule. The thresholds recommended by the CDC for children aged 19-35 months are diphtheria, tetanus, and acellular pertussis (DTaP) ≥4 doses, polio ≥3 doses, measles, mumps, and rubella (MMR) ≥1 dose, *Haemophilus influenzae* type b (Hib) ≥3 doses, hepatitis B ≥3 doses, varicella ≥1 dose, and pneumococcal conjugate (PCV) ≥4 doses. These dose thresholds were based on CDC recommendations for children aged 19-35 months and were applied consistently across all survey years (2010-2023) to ensure comparability over time.

The outcome of interest throughout our analysis was whether the child was up to date on individual vaccines. All other variables are listed in Table S1 in Multimedia Appendix 1, which were treated as covariates for understanding patterns in coverage across years. In addition to individual vaccine indicators, we created a composite variable denoted as the Combined 7-Vaccine Series, capturing whether a child was fully vaccinated with all 7 core vaccines (DTaP, polio, MMR, Hib, hepatitis B, varicella, and PCV) as defined by the CDC for national coverage monitoring. Although school entry vaccine requirements vary by state and year, this composite reflects the standardized CDC core series definition rather than uniform state mandates. Additional vaccines (hepatitis A, influenza, and rotavirus) were analyzed separately as individual outcomes. This composite variable served as a unified measure of complete immunization and played a key role in year-over-year comparisons.

Observations were restricted to children with provider-verified immunization records (using PDAT=1 as a filter), consistent with the study eligibility criteria. No additional manual row deletion was performed. Across study years, vaccine coverage variables exhibited no missing values, and most covariates demonstrated <6% missingness. Survey-weighted logistic regression models were estimated using complete-case analysis inherent to the modeling procedure, whereby observations with missing covariate values were excluded automatically at the model estimation stage.

One variable, representing the participation in the Supplemental Nutrition Program for Women, Infants, and Children (WIC) and denoted as CWIC_02, however, exhibited a markedly higher proportion of missing responses, ranging approximately from 50% to 60% across years. This pattern reflects survey flow structure and item-level nonresponse within the NIS-Child rather than data processing decisions. Because WIC participation is captured through multiple related indicators within the survey, including an alternative measure (CWIC_01) with lower missingness and comparable conceptual relevance, sensitivity analyses were conducted to evaluate whether associations were robust to alternative WIC specifications and resulting differences in analytic sample size.

### Survey Design

We incorporated the core survey design variables required to ensure valid, nationally representative estimates across all years of analysis. To maintain data integrity, only records with provider-verified data were included, reflecting the study’s eligibility criteria: children aged 19-35 months with completed NIS-Child interviews and verified immunization records from health care providers. Each year’s dataset was structured using a consistent survey design framework that aligned with the NIS-Child’s complex sampling methodology. The unique child identification was used as the primary sampling unit to account for clustering within the sample, ensuring that variance estimates reflected the survey’s multistage sampling design. A stratification variable was used to optimize precision and reduce sampling error in national estimates. Sampling weights for the children were applied to each observation to adjust for unequal probabilities of selection, survey nonresponse, and to align the weighted data with US population totals.

### Bias

Several measures were implemented to minimize potential bias. Provider verification of immunization records reduced recall and reporting bias compared to parental self-report. Survey weights accounted for differential selection probabilities and nonresponse bias. It is important to mention that the restriction to provider-verified data may have introduced selection bias toward children with better health care access.

### Analysis

Statistical analyses were conducted using survey-weighted methods to account for the complex sampling design of NIS-Child. Complete-case analysis was used within each survey-weighted model, excluding records with missing survey weights or covariate values at the estimation stage. Out of all 15 independent variables, the income-to-poverty ratio was the only variable analyzed as continuous, while the remaining 14 predictors were treated as categorical variables with predefined NIS-Child groupings. No interaction terms were included in the primary models. However, subgroup analyses and sensitivity analyses were conducted to evaluate the robustness of findings with the inclusion of interaction effects. Subgroup analyses examining associations across key demographic strata are presented in Multimedia Appendix 1. To examine annual trends in childhood immunization and assess how they relate to demographic and social factors, we used survey-weighted logistic regression models. Two sets of analyses were conducted. The first focused on the Combined 7-Vaccine Series, which is the binary variable created to reflect whether a child received all 7 core vaccines recommended by the CDC. The second set of models was run for each individual vaccine, allowing us to explore differences in coverage patterns across specific immunizations. We applied the same modeling process across all years from 2010 to 2023. This ensured consistent treatment of variables, uniform survey design application, and systematic evaluation of associations across time. All models used the appropriate sampling weights, clustering, and stratification variables provided by the NIS-Child to produce estimates that represent the US child population.

To evaluate the stability of associations related to WIC participation, 3 model specifications were examined. A base model excluded WIC participation entirely. A second model included CWIC_01, an indicator of WIC participation with lower missingness. The third model included CWIC_02, denoting current participation. Comparing these specifications allowed assessment of whether associations between vaccination coverage and social determinants were sensitive to WIC measurement and resulting analytic sample size differences.

To ensure methodological transparency, categorical predictors were coded using treatment contrasts in R software (version 2024.12.1+563; R Foundation for Statistical Computing), with the first-factor level serving as the reference category. Variables were harmonized across survey years to maintain consistent category definitions and reference groups. The income-to-poverty ratio was modeled as a continuous predictor to preserve information. Although formal spline-based testing of the linearity assumption was not performed, coefficient stability across years did not suggest substantial deviation from linearity on the logit scale. Formal variance inflation factors were not calculated within the survey-weighted framework; however, inspection of coefficient and SE stability indicated no evidence of meaningful multicollinearity. Statistical significance was evaluated at α=0.05 without formal adjustment for multiple comparisons, as each year-specific model addressed a substantively distinct survey population and temporal comparison. Interpretation focused on effect sizes, CIs, and consistency of associations across survey years rather than isolated *P* values.

The survey-weighted logistic regression models estimate the likelihood of a child being up to date on recommended vaccines while accounting for the complex sampling design of the NIS-Child. The mathematical formulation of the survey-weighted logistic regression models is provided in Section S1 in Multimedia Appendix 1.

All data processing and statistical analyses were conducted using R statistical programming [27]. The *survey* package was used to implement complex survey design and fit survey-weighted logistic regression models [28]. From this, it is important to note that variance estimation was obtained from using Taylor linearization via the indicated R package. For each survey year, a year-specific survey design object was created, and associations between vaccination coverage and contextual factors (as listed in Table S1 in Multimedia Appendix 1) were examined. This modeling process was conducted separately for each vaccine outcome variable, including the derived Combined 7-Vaccine Series indicator. To visualize trends across years, we used heat maps showing the direction and significance of associations at the 5% significance level, where green indicates statistically significant effects in the positive direction, red indicates statistically significant effects in the negative direction, and gray indicates nonsignificant results. Additional gradient heat maps were generated to display the magnitude and direction of statistically significant log-odds estimates across model specifications. Separate heat maps were also created for each vaccine. Selected figures are included in the main text of this manuscript, and additional figures are present in Multimedia Appendix 1.

### Ethical Considerations

The Indiana University Human Research Protection Program staff determined that the analysis done in this study was not human subject research and did not require further institutional review board (IRB) review before conducting the study.

This study used publicly available, deidentified data from the NIS-Child, conducted annually by the CDC. The NIS-Child protocol is reviewed and approved by the CDC IRB, and informed consent is obtained from participating households, with additional parental consent for provider record verification. Because this analysis relied on secondary, deidentified data that is publicly available, IRB review was not required. The dataset contains no personally identifiable information, and no participant compensation was provided as part of this secondary analysis.

## Results

**Figure 1:**
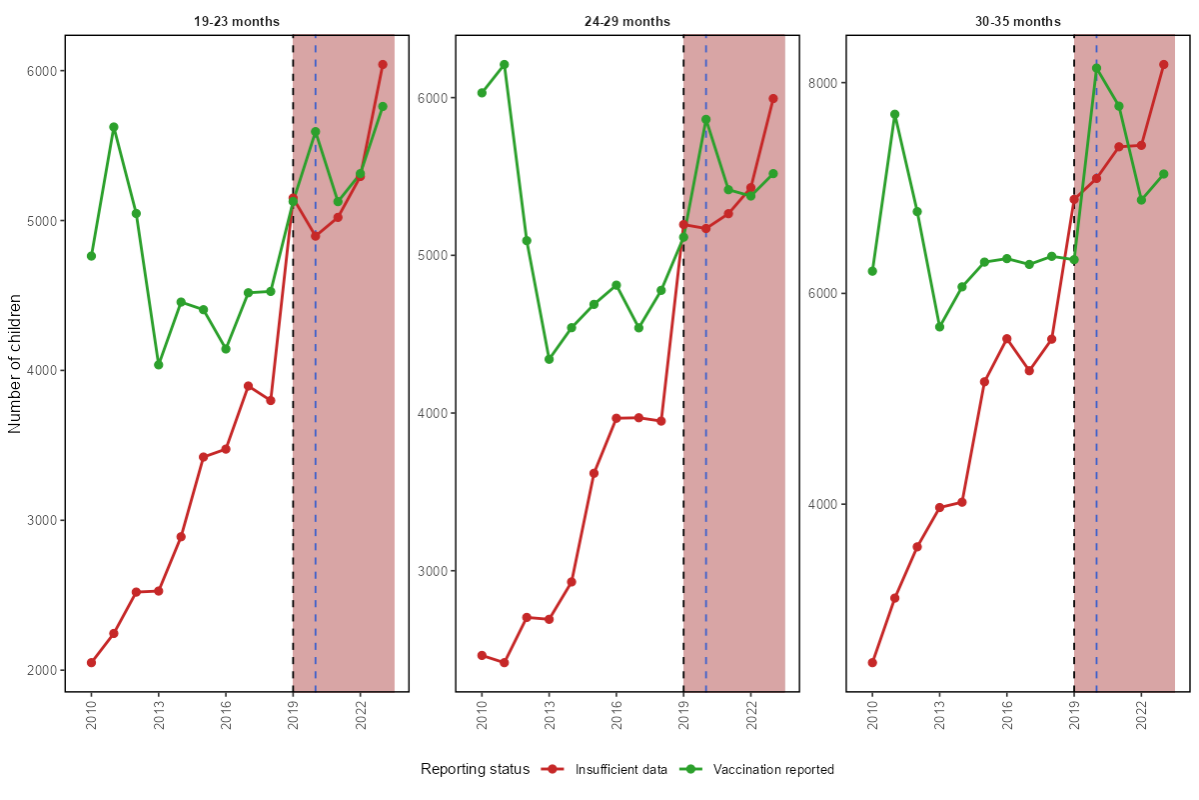
Provider-verified (green) versus insufficient provider data (red) before and after COVID-19 emergence in the United States. Insufficient provider data refers to completed interviews without adequate provider-verified vaccination records. The dashed line marks 2020; the shaded region indicates the post–COVID-19 period.

**Figure 2:**
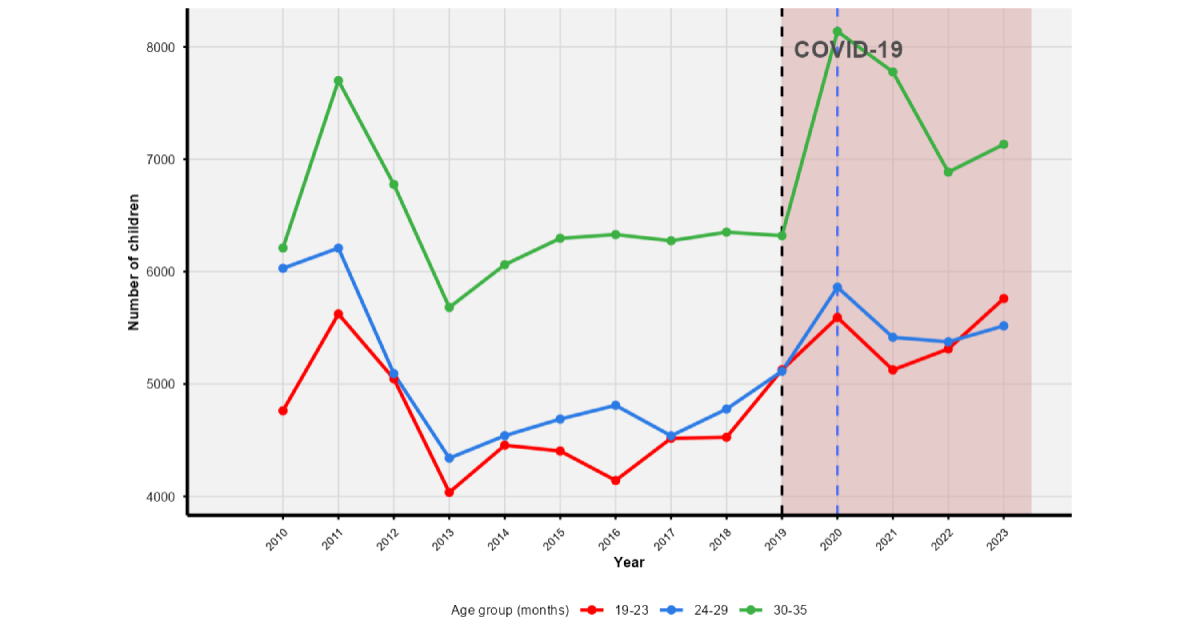
Trends in provider-verified data by age group during the study period (2010-2023), highlighting the impact of the COVID-19 pandemic.

Figure 1 shows the trends in the number of children with provider-verified vaccination data (green) versus those with insufficient provider data (red), stratified by age groups (19-23, 24-29, and 30-35 months). A notable shift is observed during the COVID-19 pandemic (highlighted region in red), with a marked increase in missing provider data across all age groups starting in 2020, which coincides with the emergence of COVID-19 in the United States. By 2023, the number of unverified records surpassed verified ones in all age categories, especially among older children (30-35 months), suggesting disruptions in provider follow-up and data collection practices during and after the pandemic. This pattern is further corroborated by Figure 2, which shows a sharp rise in insufficient provider data following the pandemic across all age groups, particularly for children aged 30-35 months. Vaccination reporting by age group, as seen in Figure 2, shows that children aged 30-35 months consistently had higher rates of up-to-date vaccination, while children aged 19-23 months lagged. The disparity widened after 2020, possibly reflecting pandemic-related disruptions to scheduled vaccinations among younger children.

**Figure 3:**
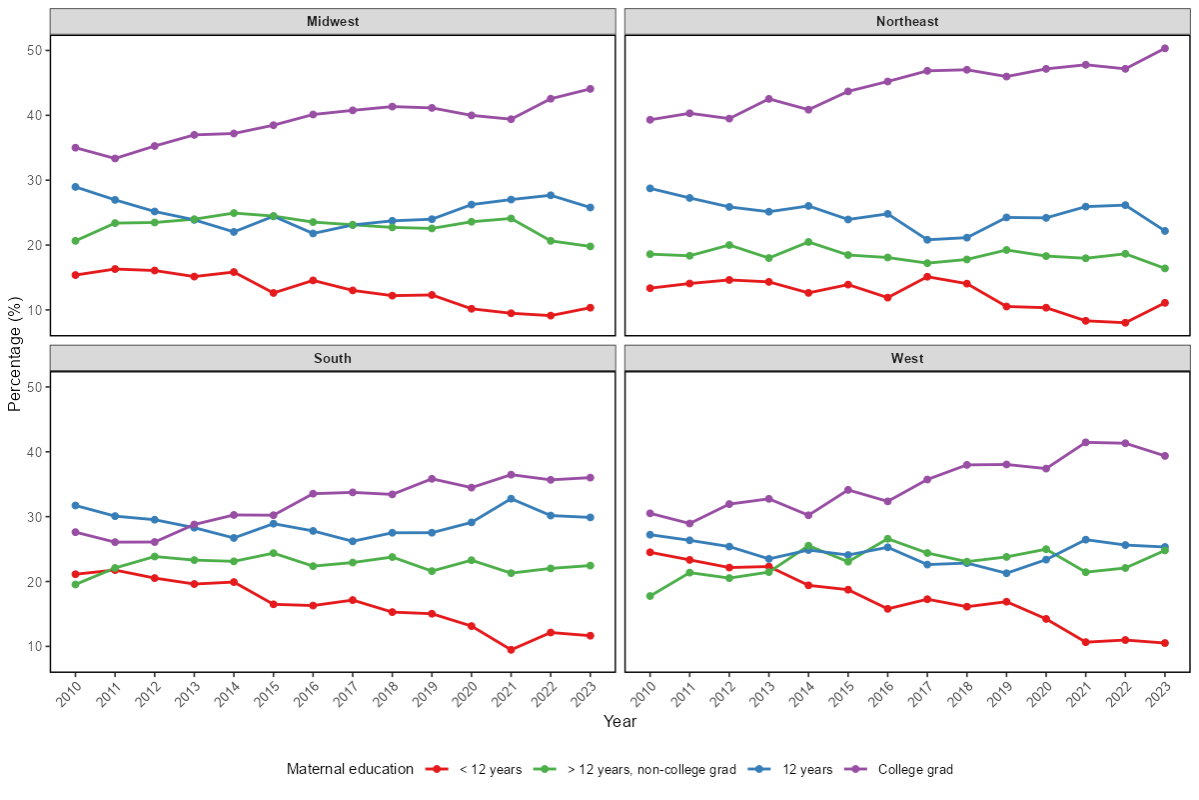
Maternal education trends by regions. grad: graduate.

**Figure 4:**
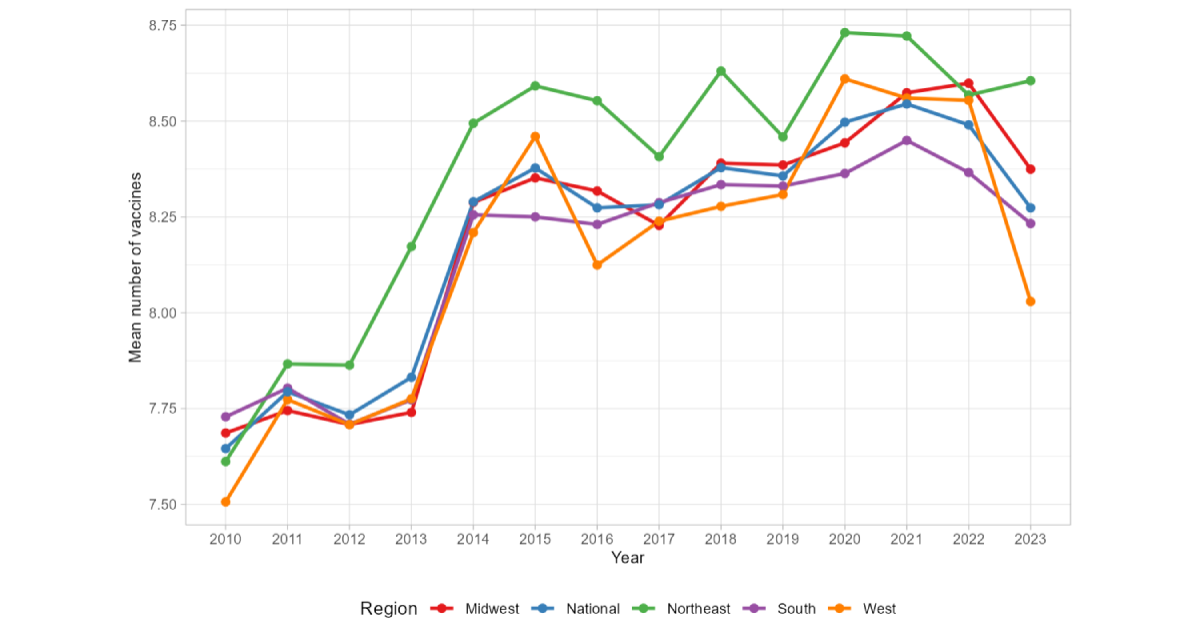
Mean number of vaccines received by children by regions.

Maternal education levels, a key variable in our models, showed consistent regional differences across time. Figure 3 illustrates that the Northeast and Midwest regions had a higher proportion of college-educated mothers, with an upward trend over time. By contrast, the South had persistently higher proportions of mothers with less than 12 years of education. These regional disparities in maternal education may contribute to the observed geographic differences in vaccination outcomes. Figure 4 presents the mean number of vaccines received per child by region. The Northeast consistently achieved higher average vaccine counts compared to other regions, while the South and West trailed behind.

Figures S5 and S6 in Multimedia Appendix 1 illustrate trends in the language of interview among Hispanic respondents and the weighted proportion of Hispanic to non-Hispanic children included in the NIS-Child survey over time. Figure S5 shows a marked shift from Spanish to English interviews among Hispanic families between 2010 and 2023. Early in the time series, Spanish-language interviews comprised a considerable proportion of the total, suggesting a need for linguistically appropriate outreach. The increasing predominance of English-language responses in later years may reflect demographic transitions or changes in data collection protocols, but could also indicate reduced inclusion of less acculturated families. Figure S6 demonstrates a gradual decline in the number of Hispanic children included in the weighted sample over the years. This trend raises questions about representation and potential undercounting of vulnerable subgroups.

**Figure 5:**
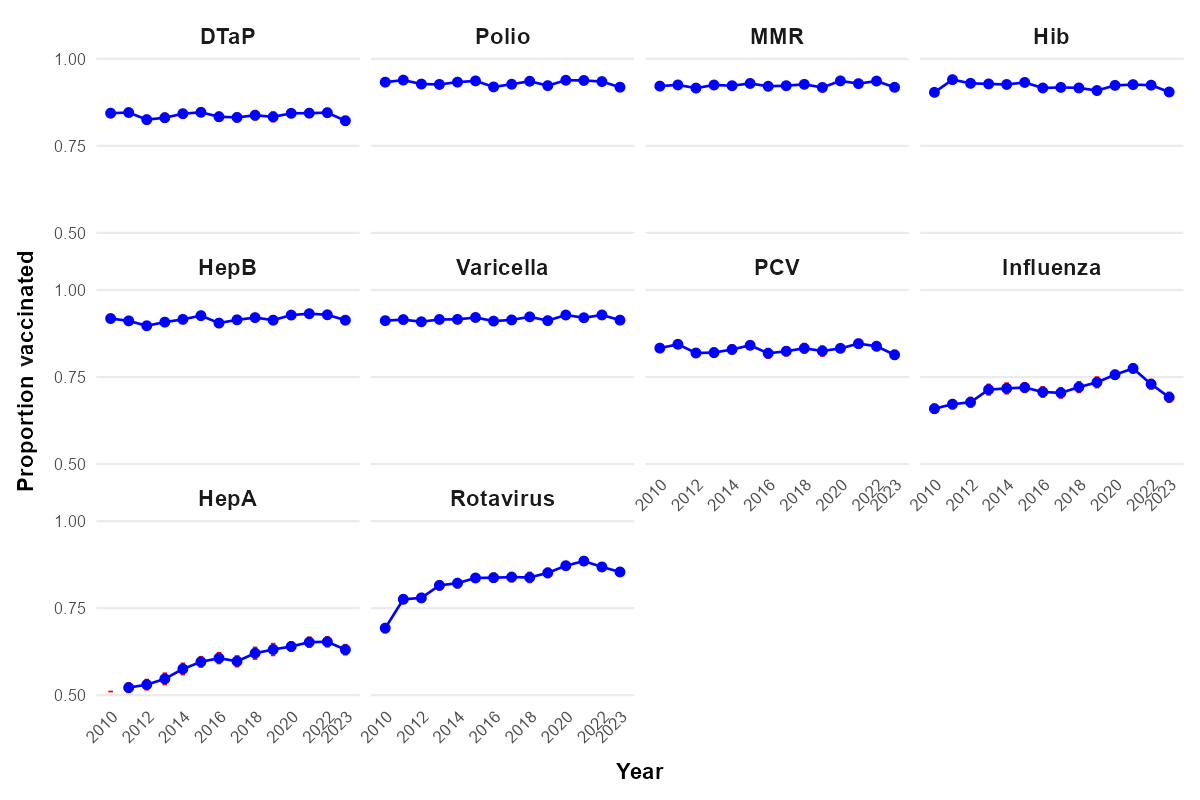
Trends in individual vaccine coverage. Year-wise (2010-2023) coverage rates for diphtheria, tetanus, and acellular pertussis (DTaP); polio; measles, mumps, and rubella (MMR); Haemophilus influenzae type b (Hib); hepatitis B; varicella; pneumococcal conjugate (PCV); influenza; hepatitis A; and rotavirus.

**Figure 6:**
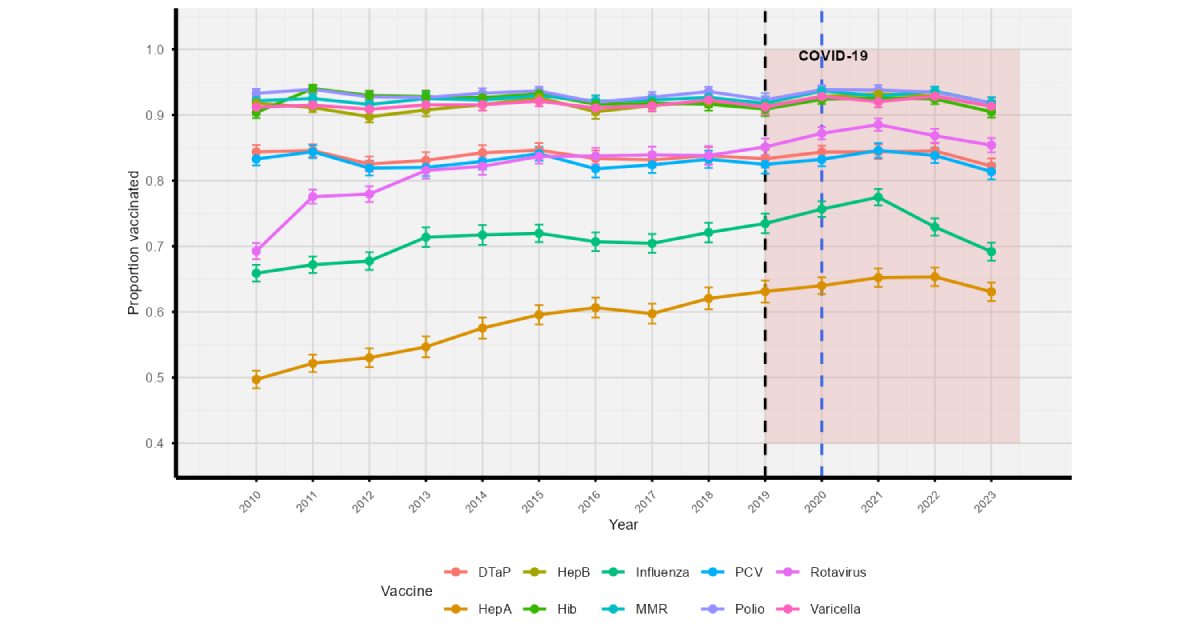
Vaccine coverage with 95% CIs. Combined coverage trend with bootstrapped confidence bands from 2010 to 2023. DTaP: diphtheria, tetanus, and acellular pertussis; HepA: hepatitis A; HepB: hepatitis B; Hib: Haemophilus influenzae type b; MMR: measles, mumps, and rubella; PCV: pneumococcal conjugate.

Figure 5 shows year-wise vaccination coverage trends for each individual vaccine. While coverage remained above 90% for core vaccines such as polio, MMR, and PCV, substantial gaps persisted for influenza and hepatitis A. Rotavirus and influenza saw visible declines in 2022 and 2023, diverging from otherwise stable trajectories. Figure 6 reinforces these trends, showing relatively tight 95% CIs for high-coverage vaccines, but wider intervals for hepatitis A and influenza.

To illustrate model-based coverage estimates, a sample of 2022 CIs is shown in Table 1. This includes estimated means and 95% CIs for each vaccine coverage. For example, polio had a mean coverage of 93.5% (95% CI 92.7%-94.3%), while influenza had 72.9% (95% CI 71.6%-74.3%). Complete year-specific coverage estimates for all vaccines are provided in Table S3 in Multimedia Appendix 1.

**Table 1:** Estimated vaccine coverage for example year 2022 with 95% CIs.

Vaccine	Mean (95% CI)
DTaP^a^	0.8453 (0.8340-0.8567)
Polio	0.9347 (0.9268-0.9426)
MMR^b^	0.9361 (0.9291-0.9432)
Hib^c^	0.9243 (0.9164-0.9322)
HepB^d^	0.9288 (0.9213-0.9364)
Varicella	0.9283 (0.9208-0.9359)
PCV^e^	0.8383 (0.8267-0.8501)
Influenza	0.7294 (0.7161-0.7427)
HepA^f^	0.6534 (0.6394-0.6675)
Rotavirus	0.8685 (0.8581-0.8788)

^a^DTaP: diphtheria, tetanus, and acellular pertussis.

^b^MMR: measles, mumps, and rubella.

^c^Hib: *Haemophilus influenzae* type b.

^d^HepB: hepatitis B.

^e^PCV: pneumococcal conjugate.

^f^HepA: hepatitis A.

The primary multivariable model included demographic, socioeconomic, household, and maternal factors as detailed in Table S1 in Multimedia Appendix 1, alongside an indicator reflecting whether the child had ever received benefits through the WIC program. This specification was selected due to lower missingness and broader representation of cumulative program exposure across survey years. Sensitivity analyses were conducted using an alternative indicator reflecting current WIC participation, which exhibited substantial structural missingness and resulted in a marked reduction in analytic sample size. A third model excluded WIC participation entirely while retaining all other social and demographic covariates to evaluate whether inclusion of WIC altered associations among core predictors. Across specifications, the direction and magnitude of non-WIC predictors remained largely consistent, supporting the stability of the primary model.

By 2023, coverage for the Combined 7-Vaccine Series reached 77.4% (14107/18032). In 2010, estimated coverage was 70.8% (SE 0.006), increasing to 71.5% (SE 0.007) by 2012 and continuing to rise thereafter.

In the primary model, older child age was consistently associated with higher odds of being fully vaccinated across the study period (2010: odds ratio [OR] 1.10, 95% CI 1.02-1.19, SE 0.040; *P*=.02; 2023: OR 1.24, 95% CI 1.13-1.36, SE 0.047; *P*<.001). Larger household size was associated with lower odds of vaccination completion in early years (2010: OR 0.89, 95% CI 0.85-0.94, SE 0.027; *P*<.001) and remained a persistent barrier across multiple survey cycles. The language of the interview demonstrated positive associations in selected years. For example, in 2010, children from households interviewed in English had higher odds of complete vaccination (OR 1.50, 95% CI 1.18-1.90, SE 0.123; *P*<.001), and this association reemerged in 2018 (OR 1.50, 95% CI 1.15-1.95, SE 0.407; *P*=.003).

Insurance coverage became increasingly influential in later years. In 2022, insured children had higher odds of complete vaccination compared with uninsured children (OR 0.74, 95% CI 0.65-0.84, SE 0.063; *P*<.001). Maternal education similarly demonstrated positive associations in recent years, indicating a widening gradient by educational attainment. Regional disparities intensified after 2020, with certain geographic regions exhibiting lower odds of complete vaccination (OR 0.74, 95% CI 0.65-0.84, SE 0.063; *P*<.001). Hispanic origin showed intermittent negative associations across survey years but was not uniformly significant throughout the study period. Overall, age-related advantages and household size barriers were the most stable predictors across time.

Influenza coverage improved from 65.9% (11470/16798) in 2010 to 77.5% (13408/17232) in 2022 before plateauing. In 2020, marital status (OR 0.75, 95% CI 0.63-0.90, SE 0.087; *P*=.001) and income-to-poverty ratio (OR 1.25, 95% CI 1.13-1.39, SE 0.054; *P*<.001) were significantly associated with influenza vaccination. Insurance coverage and regional disparities also became more prominent after 2020. In 2010, hepatitis A coverage was 49.7% (SE 0.007). Older age (2010: OR 1.94, 95% CI 1.80-2.09, SE 0.038; *P*<.001), smaller household size (2010: OR 0.90, 95% CI 0.85-0.94, SE 0.027; *P*<.001), Hispanic origin (2010: OR 0.75, 95% CI 0.63-0.89, SE 0.086; *P*=.001), language of interview (2010: OR 1.39, 95% CI 1.13-1.73, SE 0.108; *P*=.002), insurance coverage (2010: OR 0.81, 95% CI 0.80-0.98, SE 0.049; *P*=.001) and WIC benefits (2010: OR 0.69, 95% CI 0.56-0.87, SE 0.107; *P*<.001) were significantly associated with uptake. These disparities persisted in 2022 and 2023, indicating continued structural and access-related inequities. PCV and rotavirus vaccines showed persistent negative associations with increasing household size (2010; rotavirus: OR 0.74, 95% CI 0.65-0.84, SE 0.063; *P*<.001; PCV: OR 0.88, 95% CI 0.82-0.93 SE 0.030; *P*<.001), which are highly significant, suggesting logistical challenges among larger families. Regional disparities and Hispanic origin effects were also evident for these vaccines, particularly after 2020.

**Figure 7:**
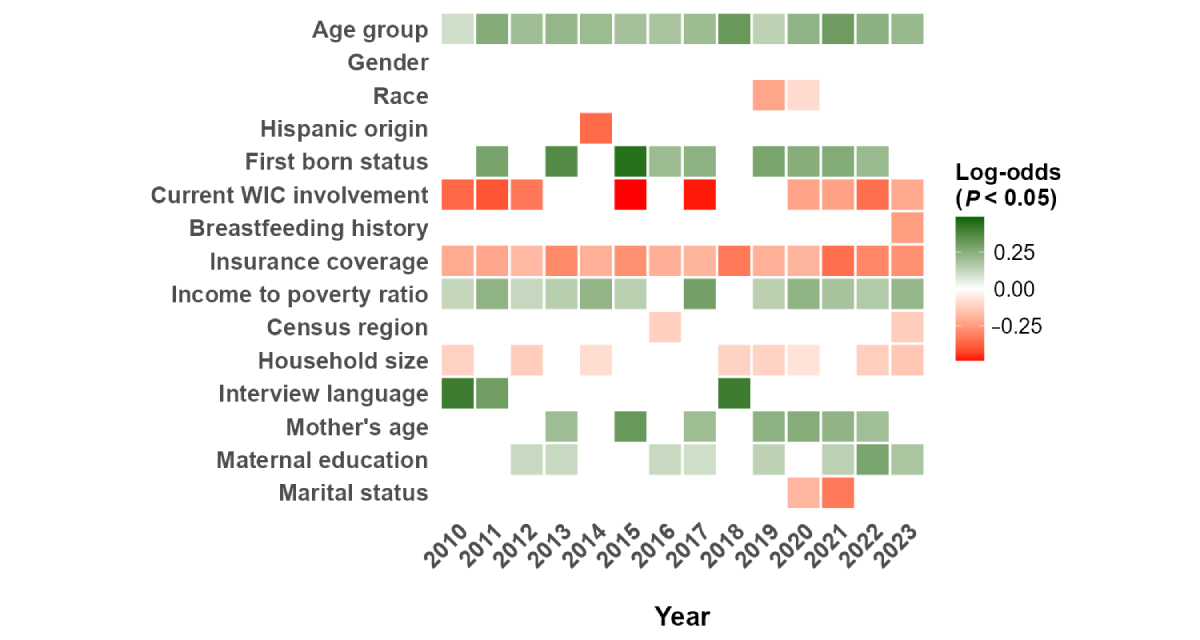
Heat map of associations between predictors and the uptake of the Combined 7-Vaccine Series. WIC: Supplemental Nutrition Program for Women, Infants, and Children.

For MMR vaccination, the strength of age-related associations declined over time. In 2018, age group was strongly associated with higher odds of vaccination (OR 1.37, 95% CI 1.20-1.57, SE 0.069; *P*<.001), and this association remained significant in 2022 (OR 1.34, 95% CI 1.14-1.57, SE 0.081; *P*<.001). However, by 2023, the association weakened and became nonsignificant (OR 1.12, 95% CI 0.97-1.31, SE 0.076; *P*=.12). Participation in WIC demonstrated notable temporal shifts. In 2013, WIC participation was associated with significantly lower odds of MMR vaccination (OR 0.63, 95% CI 0.44-0.90, SE 0.180; *P*=.01). This inverse association attenuated in subsequent years and was not statistically significant in 2016 (OR 0.64, 95% CI 0.40-1.03, SE 0.241; *P*=.06). During the COVID-19 period, the direction of association shifted toward higher vaccination likelihood among WIC participants, although not reaching statistical significance in 2022 (OR 0.72, 95% CI 0.49-1.05, SE 0.194; *P*=.09) and 2023 (OR 0.83, 95% CI 0.62-1.11, SE 0.151; *P*=.21). These temporal fluctuations suggest that socioeconomic program engagement and age-based eligibility effects on MMR vaccination evolved over the study period, particularly during the pandemic years. Additional heat maps for individual vaccines are presented in Figures S6-S15 in Multimedia Appendix 1, demonstrating statistical significance patterns of SDoH across each vaccine type. These disparities remained evident across the study period when looking at the Combined 7-Vaccine Series, reinforcing the persistence of structural and access-related barriers, as seen in Figure 7. Complete table of effect sizes, CIs, and *P* values is provided in Multimedia Appendix 1.

Sensitivity analyses using the current-participation WIC measure yielded comparable directional effects but wider CIs due to reduced sample size. The base model excluding WIC showed no material changes in non-WIC predictor associations, indicating that WIC inclusion did not distort relationships among core social determinants. Detailed model comparisons and corresponding association patterns are provided in Multimedia Appendix 1, where year-specific estimates across WIC specifications are presented.

## Discussion

### Principal Findings

This cross-sectional analysis of provider-verified NIS-Child data from 2010 to 2023 documents persistent associations between childhood vaccination coverage and multiple SDoH across the United States. Although coverage for several core vaccines remained high, consistent socioeconomic, regional, and language-related gradients were observed across survey years. These findings describe associations rather than causal effects and highlight structural patterns that may contribute to inequities in immunization uptake.

Maternal education was consistently associated with higher odds of being up to date on vaccines. This pattern aligns with global evidence identifying maternal education as an important determinant of child health outcomes, including immunization coverage [29]. Higher educational attainment may reflect differences in health literacy, access to reliable information, and the ability to navigate health care systems. Caregivers with more education may be better positioned to understand vaccine schedules, communicate with providers, and respond to reminders, which may facilitate timely immunization.

Household income-to-poverty ratio was similarly associated with vaccination coverage, consistent with prior research documenting economic disparities in immunization [30]. Financial constraints may limit transportation access, appointment flexibility, and continuity of care. Even when vaccines are available at low or no cost, structural barriers such as unstable employment, limited clinic availability, and neighborhood-level resource gaps may shape preventive service use. Insurance status demonstrated strong associations, particularly in later survey years. According to a nationwide analysis, children from higher-income families were more likely to complete the full vaccine schedule on time compared to those in lower-income households. Insurance status was another factor with a strong association [31]. Uninsured children were less likely to be fully vaccinated compared to those with public or private coverage. CDC reports have similarly noted lower immunization rates among uninsured children despite the presence of the Vaccines for Children program [32]. Insurance coverage may therefore reflect broader system engagement and continuity of pediatric care.

Ethnicity-related differences were observed for several vaccines, including hepatitis A, influenza, and rotavirus. Hispanic origin was intermittently associated with lower vaccination odds, though not uniformly across all years. These findings are consistent with the literature attributing disparities in preventive care to structural barriers, including language discordance, immigration-related concerns, and differential provider engagement. The Hispanic paradox, which refers to the phenomenon in which Hispanic individuals experience health outcomes that are unexpectedly favorable relative to their socioeconomic status, has been well documented in outcomes such as mortality and chronic disease. However, these results indicate that this paradox does not extend to preventive care measures like childhood immunization, where disparities in access and use remain prominent [33,34]. Preventive services such as vaccination require active health care system engagement and documentation, which may be more sensitive to structural access barriers than mortality outcomes.

Language of interview was associated with vaccination coverage in selected years, with non–English-speaking households less likely to achieve complete immunization. This supports evidence that language discordance between caregivers and providers may contribute to miscommunication, reduced trust, and missed vaccination opportunities [35]. However, the language of the interview likely reflects broader structural barriers beyond communication alone and should be interpreted as a proxy marker rather than a purely linguistic effect. Improvements in culturally and linguistically appropriate services may therefore play an important role in reducing disparities.

Regional variation persisted throughout the study period and appeared more pronounced after 2020. Differences across census regions likely reflect variation in health care infrastructure, provider density, rural-urban distribution, and state-level immunization policies. Lower vaccination coverage in certain regions may be related to previously documented geographic disparities in pediatric service access, particularly in rural or underserved areas where limited provider availability and long travel distances create barriers to timely immunization [36]. Regional heterogeneity in vaccination coverage may therefore represent structural differences in health care delivery rather than individual-level preferences alone. While regional analyses provide stable comparisons across years, they may mask meaningful state-level variation; future research should examine state-specific differences to better inform policy decisions.

Household size was consistently associated with lower vaccination completion for several vaccines, including PCV and rotavirus. Larger households may face logistical challenges related to scheduling, transportation, and competing caregiving demands. Age-related differences in coverage likely reflect timing of eligibility and health care encounters rather than behavioral differences.

The COVID-19 period coincided with increased insufficient provider data and widening disparities for selected vaccines, particularly influenza and hepatitis A. Disruptions in routine care and shifts in health care use during this period may have influenced vaccination patterns. Recent CDC reports indicate declines in kindergarten vaccination coverage and rising exemption rates, reinforcing concerns about sustained population-level protection. MMR coverage dropped from 93.1% (17931/19342) to 92.7% (17931/18032), a decrease of 0.4 percentage points), while DTaP coverage fell from 92.7% (14909/17232) to 92.3% (15903/18032), also a 0.4 percentage point decline) between the 2022-2023 and 2023-2024 school years. Simultaneously, exemption rates reached a record high of 3.3%, up from 3.0% the previous year [37]. The findings of this study reinforce the need to address coverage disparities through targeted public health strategies. Although the analytic sample size remained stable and standard survey weighting procedures were applied, estimates from 2020-2023 should be interpreted with caution, given potential shifts in provider participation and survey response dynamics during the pandemic period.

As a cross-sectional analysis, this study documents associations between social determinants and vaccination coverage but cannot establish causal relationships. Longitudinal or quasi-experimental studies are needed to determine whether interventions targeting identified social and structural barriers would improve immunization uptake. Given the number of year-specific models estimated, findings should be interpreted in the context of temporal consistency rather than reliance on isolated statistical significance. Overall, the persistence of disparities across education, income, insurance, language, and region suggests that structural inequities remain central to understanding gaps in childhood vaccination coverage.

### Limitations

This study has several limitations. First, the cross-sectional design limits inference to associations and does not establish causality. Second, analyses were restricted to children with provider-verified vaccination records, consistent with NIS-Child guidance; however, if children without verified records differ systematically in health care access or sociodemographic characteristics, disparities may be underestimated. Survey-weighted regression models were estimated using complete-case analysis. Although most covariates had low missingness, bias may occur if the data were not missing at random. WIC participation required additional consideration. While the primary WIC indicator used in the main models had relatively low missingness, an alternative WIC variable exhibited substantial structural missingness due to survey design. Sensitivity analyses indicated that this missingness influenced certain associations, particularly involving insurance status, suggesting that the missingness mechanism was not random. However, associations for other core social determinants remained stable across alternative model specifications, including a model excluding WIC, supporting the robustness of the primary findings.

Changes in provider verification patterns and health care use during 2020 to 2023 may affect the interpretation of year-specific estimates, even though provider-phase weights and a consistent analytic framework were applied across all years. Several relevant factors, including immigration status, health literacy, vaccine confidence, and access barriers, were not captured in the public dataset, raising the possibility of unmeasured confounding. Regional variables were measured at an aggregate level and may be subject to ecological bias. Statistical significance was assessed at 5% without formal adjustment for multiple comparisons, and findings should be interpreted with emphasis on effect sizes and consistency over time. Finally, generalizability is limited to US children aged 0-35 months with provider-verified records and may not extend to other populations or health systems.

### Future Work

Future research should build on these findings by using longitudinal and quasi-experimental approaches to better understand how structural and policy changes influence vaccination disparities over time. Qualitative and mixed method studies may help clarify mechanisms not captured in NIS-Child, including health literacy, cultural mistrust, transportation barriers, and health care navigation challenges that may contribute to undervaccination among Hispanic, uninsured, and low-income families [38]. State-level and geospatial analyses are also needed to examine within-region heterogeneity and link disparities to policy environments such as Medicaid eligibility, exemption laws, and public health funding differences across states [39]. Incorporating interaction analyses in future models as part of a formal causal inference model, such as ethnicity by language or insurance by income, may further identify subgroups experiencing compounded barriers and inform more targeted interventions.

### Conclusion

This study used nationally representative, provider-verified NIS-Child data from 2010 to 2023 to examine associations between SDoH and early childhood vaccination coverage of CDC recommended vaccines, including completion of a Combined 7-Vaccine Series. Across survey years, vaccination coverage was consistently patterned by maternal education, insurance status, household income, language of interview, and region, with particularly persistent gaps for hepatitis A, influenza, and rotavirus. Although provider verification decreased after 2020, the analytic sample of children with adequate provider data remained stable, and results were generally robust across sensitivity specifications. These findings support the need for equity-focused immunization strategies that address structural barriers to accessing preventive care, including insurance coverage, culturally and linguistically appropriate services, and geographically tailored outreach. Continued surveillance and policy-relevant analyses are needed to monitor whether disparities narrow over time and to guide interventions that improve protection against vaccine-preventable diseases for all children.

## Appendix

Multimedia Appendix 1 Additional tables, figures, and information.

## Abbreviations

CDC: Centers for Disease Control and Prevention
CWIC: Supplemental Nutrition Program for Women, Infants, and Children measure
DTaP: diphtheria, tetanus, and acellular pertussis
Hib: Haemophilus influenzae type b
IRB: institutional review board
MMR: measles, mumps, and rubella
NIS-Child: National Immunization Survey-Child
OR: odds ratio
PCV: pneumococcal conjugate
PDAT: adequate provider data identifier
SDoH: social determinants of health
WIC: Supplemental Nutrition Program for Women, Infants, and Children

## Data Availability

The data used for this study were sourced from the National Immunization Surveys database, available on the Centers for Disease Control and Prevention (CDC) website. Access to the dataset can be obtained from the CDC’s official data repository, ensuring transparency and reproducibility in research findings. The study involved secondary analysis of publicly available, deidentified surveillance data. No new datasets were generated. Statistical code used for data processing and analysis is available from the corresponding author on reasonable request.
