# Supplementary material for: Social Determinants of Childhood Vaccination Coverage in the United States Using National Immunization Survey Data From 2010 to 2023: Cross-Sectional Study
Source: JMIR Public Health Surveill. 2026 Apr 9;12:e81746. doi: 10.2196/81746 (PMC13107105; doi:10.2196/81746)
Supplement: Multimedia Appendix 1 [file publichealth_v12i1e81746_app1.docx]

**[Supplemental Materials]**

**Social Determinants of Childhood Vaccination Coverage in the United States Using National Immunization Survey Data From 2010 to 2023: Cross-Sectional Study**

Ramya Keerthi Majji^1^, Yan Zhuang^1^, and Felix M. Pabon-Rodriguez^1,2^*

^1^Department of Biomedical Engineering and Informatics, Indiana University, Luddy School of Informatics, Computing, and Engineering, Indianapolis, Indiana, United States

^2^Department of Biostatistics and Health Data Science, Indiana University, School of Medicine, Indianapolis, Indiana, United States

**MATHEMATICAL FORMULATION**

**S1. Survey-Weighted Logistic Regression Formulation**

The general formulation of the logistic regression model used is:

$logit (P\left( Y_{i}=1 | \boldsymbol{X}_{i} \right))=\boldsymbol{X}_{i}^{'}\boldsymbol{\beta=}\beta_{0}+\beta_{1}X_{i1}+\beta_{1}X_{i2}+\ldots+\beta_{p}X_{ip}$ for $i=1,\ldots,n$

where $\pi_{i} := P\left( Y_{i}=1 | \boldsymbol{X}_{i} \right)=\frac{1}{1+exp(-\boldsymbol{X}_{i}^{'}\boldsymbol{\beta})}$. The outcome variable $Y_{i}$ indicates whether child $i$ is up to date on a specific vaccine, while the predictors $X_{1}, X_{2},\ldots,X_{p}$ represent the covariates included in the model, such as age group, maternal education, household income-to-poverty ratio, and language of interview. The coefficients $\beta_{0}, \beta_{1},\ldots,\beta_{p}$ are the estimated effects of these covariates. The model accounts for the complex survey design by incorporating sampling weights, cluster IDs, and strata. Each child $i$ is assigned a survey weight $w_{i}$, which reflects the inverse of its probability of selection (often adjusted for non-response and post-stratification). The pseudo log-likelihood is:

$$l\left( \boldsymbol{\beta} \right)=\sum_{i=1}^{n} w_{i}[Y_{i}\log\left( \pi_{i} \right)+(1-Y_{i})log(1-\pi_{i})]$$

This pseudo log-likelihood is maximized to obtain $\hat{\boldsymbol{\beta}}$. To avoid issues with the variance estimation, which leads to issues with the standard errors, confidence intervals, and *P*-values, we also accounted for both the strata and sampling unit. If we assume $H$ stratums indexed by $h$, and $C_{h}$ primary sampling units within stratum $h$, then the full sample is partitioned as follows, where $S_{hc}$ are the set of all children in the PSU $c$ of stratum $h$:

$$\left\{ 1,\ldots,n \right\}=\bigcup_{h=1}^{H} \bigcup_{c=1}^{C_{h}} S_{hc}$$

While the estimation is done via the pseudo-likelihood above, the variance-covariance matrix of $\hat{\boldsymbol{\beta}}$ is estimated using Taylor linearization, which rely on the strata and PSU structure. Mathematically, this is:

$$\hat{var(\hat{\boldsymbol{\beta}})}=\sum_{h=1}^{H} \sum_{c=1}^{C_{h}} (\boldsymbol{u}_{hc}-\bar{\boldsymbol{u}_{h}})(\boldsymbol{u}_{hc}-\bar{\boldsymbol{u}_{h}})'$$

where $\boldsymbol{u}_{hc}$ is the score contribution from PSU $c$ in stratum $h$, and $\bar{\boldsymbol{u}_{h}}=\frac{1}{C_{h}}\sum_{c=1}^{C_{h}} \boldsymbol{u}_{hc}$, the average score in the stratum $h$.

**SUPPLEMENTARY TABLES**

Table S1: Social determinants of health variables used in Survey-Weighted Logistic Regression Models.

| **Variable Name (Codebook)** | **Description** |
| --- | --- |
| AGEGRP | Child’s age grouped into ranges |
| SEX | Child’s Gender |
| RACE_K | Racial identity categories |
| I_HISP_K | Indicator for Hispanic ethnicity |
| FRSTBRN | Whether the child is the firstborn |
| CWIC_01 | Indicates if the child ever received WIC |
| CWIC_02 | Indicates if the child receives WIC benefits currently |
| CBF_01 | Indicates if the child was ever breastfed |
| INCPORAR | Ratio of family income to federal poverty threshold |
| CEN_REG | Region of residence (Northeast, Midwest, South, West) |
| INS_STAT2_I | Consolidated insurance status indicator |
| C1R | Total number of people in the household |
| LANGUAGE | Language in which the interview was conducted |
| M_AGEGRP2 | Age of the child’s mother at the time of interview |
| EDUC1 | Highest education level attained by the child’s mother |
| MARTIAL2 | Marital status of the child’s mother |

Table S2: Percentage of provider-verified and non-provider verified records from 2010- 2023.

| **Year** | **Provider- verified data** | **Non- verified data** |
| --- | --- | --- |
| 2010 | 70.81 | 29.19 |
| 2011 | 71.54 | 28.46 |
| 2012 | 65.73 | 34.27 |
| 2013 | 90.48 | 39.52 |
| 2014 | 60.48 | 39.51 |
| 2015 | 55.78 | 44.22 |
| 2016 | 54.01 | 45.98 |
| 2017 | 53.87 | 46.13 |
| 2018 | 54.04 | 45.95 |
| 2019 | 49.00 | 51.00 |
| 2020 | 53.31 | 46.68 |
| 2021 | 50.90 | 49.11 |
| 2022 | 49.22 | 50.48 |
| 2023 | 47.68 | 52.32 |

Table S3: Estimated vaccine coverage for all the years from 2010-2023 with 95% confidence intervals.

| **Vaccine** | **Year** | **Mean** | **Lower CI** | **Upper CI** |
| --- | --- | --- | --- | --- |
| DTaP | 2023 | 0.8221 | 0.8104 | 0.8339 |
| Polio | 2023 | 0.9185 | 0.9099 | 0.9272 |
| MMR | 2023 | 0.9183 | 0.9100 | 0.9266 |
| Hib | 2023 | 0.9048 | 0.8960 | 0.9136 |
| Hep B | 2023 | 0.9132 | 0.9046 | 0.9219 |
| Varicella | 2023 | 0.9134 | 0.9050 | 0.9218 |
| PCV | 2023 | 0.8138 | 0.8016 | 0.8260 |
| Influenza | 2023 | 0.6918 | 0.6781 | 0.7056 |
| Hep A | 2023 | 0.6307 | 0.6165 | 0.6448 |
| Rotavirus | 2023 | 0.8540 | 0.8431 | 0.8649 |
| **Vaccine** | **Year** | **Mean** | **Lower CI** | **Upper CI** |
| DTaP | 2022 | 0.8453 | 0.8340 | 0.8567 |
| Polio | 2022 | 0.9347 | 0.9268 | 0.9426 |
| MMR | 2022 | 0.9361 | 0.9291 | 0.9432 |
| Hib | 2022 | 0.9243 | 0.9164 | 0.9322 |
| Hep B | 2022 | 0.9288 | 0.9213 | 0.9364 |
| Varicella | 2022 | 0.9283 | 0.9208 | 0.9359 |
| PCV | 2022 | 0.8383 | 0.8267 | 0.8501 |
| Influenza | 2022 | 0.7294 | 0.7161 | 0.7427 |
| Hep A | 2022 | 0.6534 | 0.6394 | 0.6675 |
| Rotavirus | 2022 | 0.8685 | 0.8581 | 0.8788 |

| **Vaccine** | **Year** | **Mean** | **Lower CI** | **Upper CI** |
| --- | --- | --- | --- | --- |
| DTaP | 2021 | 0.8440 | 0.8330 | 0.8550 |
| Polio | 2021 | 0.9380 | 0.9380 | 0.9453 |
| MMR | 2021 | 0.9288 | 0.9288 | 0.9369 |
| Hib | 2021 | 0.9260 | 0.9260 | 0.9341 |
| Hep B | 2021 | 0.9320 | 0.9319 | 0.9392 |
| Varicella | 2021 | 0.9203 | 0.9203 | 0.9289 |
| PCV | 2021 | 0.8460 | 0.8459 | 0.8570 |
| Influenza | 2021 | 0.7747 | 0.7747 | 0.7872 |
| Hep A | 2021 | 0.6523 | 0.6523 | 0.6664 |
| Rotavirus | 2021 | 0.8853 | 0.8853 | 0.8948 |
| **Vaccine** | **Year** | **Mean** | **Lower CI** | **Upper CI** |
| DTaP | 2020 | 0.8435 | 0.8340 | 0.8530 |
| Polio | 2020 | 0.9386 | 0.9321 | 0.9450 |
| MMR | 2020 | 0.9367 | 0.9303 | 0.9430 |
| Hib | 2020 | 0.9238 | 0.9167 | 0.9309 |
| Hep B | 2020 | 0.9280 | 0.9208 | 0.9351 |
| Varicella | 2022 | 0.9282 | 0.9215 | 0.9349 |
| PCV | 2020 | 0.8323 | 0.8220 | 0.8427 |
| Influenza | 2020 | 0.7566 | 0.7448 | 0.7684 |
| Hep A | 2020 | 0.6400 | 0.6273 | 0.6527 |
| Rotavirus | 2020 | 0.8720 | 0.8626 | 0.8814 |
| **Vaccine** | **Year** | **Mean** | **Lower CI** | **Upper CI** |
| DTaP | 2019 | 0.8333 | 0.8196 | 0.8499 |
| Polio | 2019 | 0.9230 | 0.9130 | 0.9330 |
| MMR | 2019 | 0.9178 | 0.9071 | 0.9284 |
| Hib | 2019 | 0.9090 | 0.8982 | 0.9198 |
| Hep B | 2019 | 0.9134 | 0.9026 | 0.9241 |
| Varicella | 2019 | 0.9121 | 0.9014 | 0.9228 |
| PCV | 2019 | 0.8247 | 0.8107 | 0.8387 |
| Influenza | 2019 | 0.7347 | 0.7198 | 0.7496 |
| Hep A | 2019 | 0.6312 | 0.6144 | 0.6479 |
| Rotavirus | 2019 | 0.8514 | 0.8385 | 0.8643 |
| **Vaccine** | **Year** | **Mean** | **Lower CI** | **Upper CI** |
| DTaP | 2018 | 0.8379 | 0.8250 | 0.8509 |
| Polio | 2018 | 0.9357 | 0.9284 | 0.9429 |
| MMR | 2018 | 0.9267 | 0.9181 | 0.9354 |
| Hib | 2018 | 0.9165 | 0.9068 | 0.9261 |
| Hep B | 2018 | 0.9207 | 0.9117 | 0.9297 |
| Varicella | 2018 | 0.9229 | 0.9150 | 0.9307 |
| PCV | 2018 | 0.8325 | 0.8192 | 0.8458 |
| Influenza | 2018 | 0.7210 | 0.7061 | 0.7358 |
| Hep A | 2018 | 0.6206 | 0.6040 | 0.6372 |
| Rotavirus | 2018 | 0.8383 | 0.8240 | 0.8526 |

| **Vaccine** | **Year** | **Mean** | **Lower CI** | **Upper CI** |
| --- | --- | --- | --- | --- |
| DTaP | 2017 | 0.8316 | 0.8201 | 0.8432 |
| Polio | 2017 | 0.9271 | 0.9191 | 0.9350 |
| MMR | 2017 | 0.9228 | 0.9147 | 0.9310 |
| Hib | 2017 | 0.9180 | 0.9095 | 0.9265 |
| Hep B | 2017 | 0.9143 | 0.9058 | 0.9232 |
| Varicella | 2017 | 0.9141 | 0.9057 | 0.9225 |
| PCV | 2017 | 0.8241 | 0.8116 | 0.8365 |
| Influenza | 2017 | 0.7044 | 0.6902 | 0.7187 |
| Hep A | 2017 | 0.5973 | 0.5821 | 0.6126 |
| Rotavirus | 2017 | 0.8392 | 0.8265 | 0.8519 |
| **Vaccine** | **Year** | **Mean** | **Lower CI** | **Upper CI** |
| DTaP | 2016 | 0.8336 | 0.8212 | 0.8461 |
| Polio | 2016 | 0.9193 | 0.9096 | 0.9290 |
| MMR | 2016 | 0.9211 | 0.9125 | 0.9298 |
| Hib | 2016 | 0.9163 | 0.9073 | 0.9252 |
| Hep B | 2016 | 0.9050 | 0.8942 | 0.9158 |
| Varicella | 2016 | 0.9107 | 0.9012 | 0.9201 |
| PCV | 2016 | 0.8180 | 0.8046 | 0.8314 |
| Influenza | 2016 | 0.7070 | 0.6927 | 0.7212 |
| Hep A | 2016 | 0.6065 | 0.5913 | 0.6217 |
| Rotavirus | 2016 | 0.8375 | 0.8251 | 0.8500 |
| **Vaccine** | **Year** | **Mean** | **Lower CI** | **Upper CI** |
| DTaP | 2015 | 0.8464 | 0.8355 | 0.8572 |
| Polio | 2015 | 0.9367 | 0.9303 | 0.9430 |
| MMR | 2015 | 0.9293 | 0.9217 | 0.9369 |
| Hib | 2015 | 0.9323 | 0.9258 | 0.9387 |
| Hep B | 2015 | 0.9264 | 0.9191 | 0.9336 |
| Varicella | 2015 | 0.9211 | 0.9134 | 0.9287 |
| PCV | 2015 | 0.8411 | 0.8305 | 0.8517 |
| Influenza | 2015 | 0.7198 | 0.7064 | 0.7331 |
| Hep A | 2015 | 0.5957 | 0.5811 | 0.6103 |
| Rotavirus | 2015 | 0.8368 | 0.8258 | 0.8478 |
| **Vaccine** | **Year** | **Mean** | **Lower CI** | **Upper CI** |
| DTaP | 2014 | 0.8424 | 0.8309 | 0.8539 |
| Polio | 2014 | 0.9331 | 0.9255 | 0.9407 |
| MMR | 2014 | 0.9228 | 0.9144 | 0.9312 |
| Hib | 2014 | 0.9264 | 0.9183 | 0.9344 |
| Hep B | 2014 | 0.9156 | 0.9071 | 0.9242 |
| Varicella | 2014 | 0.9156 | 0.9069 | 0.9243 |
| PCV | 2014 | 0.8292 | 0.8167 | 0.8417 |
| Influenza | 2014 | 0.7172 | 0.7021 | 0.7323 |
| Hep A | 2014 | 0.5754 | 0.5594 | 0.5913 |
| Rotavirus | 2014 | 0.8218 | 0.8089 | 0.8345 |

| **Vaccine** | **Year** | **Mean** | **Lower CI** | **Upper CI** |
| --- | --- | --- | --- | --- |
| DTaP | 2013 | 0.8308 | 0.8182 | 0.8434 |
| Polio | 2013 | 0.9265 | 0.9170 | 0.9361 |
| MMR | 2013 | 0.9249 | 0.9159 | 0.9339 |
| Hib | 2013 | 0.9279 | 0.9187 | 0.9372 |
| Hep B | 2013 | 0.9077 | 0.8980 | 0.9174 |
| Varicella | 2013 | 0.9154 | 0.9060 | 0.9248 |
| PCV | 2013 | 0.8201 | 0.8070 | 0.8332 |
| Influenza | 2013 | 0.7138 | 0.6989 | 0.7287 |
| Hep A | 2013 | 0.5468 | 0.5308 | 0.5629 |
| Rotavirus | 2013 | 0.8155 | 0.8031 | 0.8279 |
| **Vaccine** | **Year** | **Mean** | **Lower CI** | **Upper CI** |
| DTaP | 2012 | 0.8253 | 0.8138 | 0.8368 |
| Polio | 2012 | 0.9276 | 0.9203 | 0.9349 |
| MMR | 2012 | 0.9160 | 0.9079 | 0.9240 |
| Hib | 2012 | 0.9297 | 0.9224 | 0.9369 |
| Hep B | 2012 | 0.8972 | 0.8889 | 0.9056 |
| Varicella | 2012 | 0.9087 | 0.9007 | 0.9167 |
| PCV | 2012 | 0.8190 | 0.8077 | 0.8303 |
| Influenza | 2012 | 0.6775 | 0.6640 | 0.6910 |
| Hep A | 2012 | 0.5303 | 0.5158 | 0.5447 |
| Rotavirus | 2012 | 0.7795 | 0.7675 | 0.7915 |
| **Vaccine** | **Year** | **Mean** | **Lower CI** | **Upper CI** |
| DTaP | 2011 | 0.8457 | 0.8360 | 0.8555 |
| Polio | 2011 | 0.9389 | 0.9330 | 0.9450 |
| MMR | 2011 | 0.9251 | 0.9184 | 0.9317 |
| Hib | 2011 | 0.9402 | 0.9343 | 0.9462 |
| Hep B | 2011 | 0.9113 | 0.9042 | 0.9184 |
| Varicella | 2011 | 0.9150 | 0.9082 | 0.9218 |
| PCV | 2011 | 0.8440 | 0.8344 | 0.8536 |
| Influenza | 2011 | 0.6719 | 0.6593 | 0.6845 |
| Hep A | 2011 | 0.5217 | 0.5084 | 0.5350 |
| Rotavirus | 2011 | 0.7755 | 0.7648 | 0.7863 |
| **Vaccine** | **Year** | **Mean** | **Lower CI** | **Upper CI** |
| DTaP | 2010 | 0.8438 | 0.8335 | 0.8541 |
| Polio | 2010 | 0.9330 | 0.9263 | 0.9397 |
| MMR | 2010 | 0.9217 | 0.9148 | 0.9285 |
| Hib | 2010 | 0.9038 | 0.8953 | 0.9122 |
| Hep B | 2010 | 0.9180 | 0.9108 | 0.9253 |
| Varicella | 2010 | 0.9119 | 0.9040 | 0.9197 |
| PCV | 2010 | 0.8330 | 0.8230 | 0.8430 |
| Influenza | 2010 | 0.6590 | 0.6462 | 0.6716 |
| Hep A | 2010 | 0.4972 | 0.4839 | 0.5105 |
| Rotavirus | 2010 | 0.6926 | 0.6802 | 0.7050 |

**SUPPLEMENTARY FIGURES**


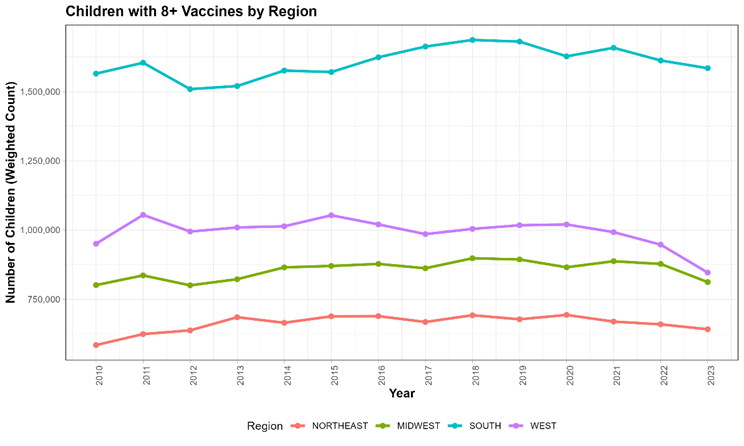


Figure S1: Weighted number of children with 0 reported vaccines by US census region from 2010 to 2023.


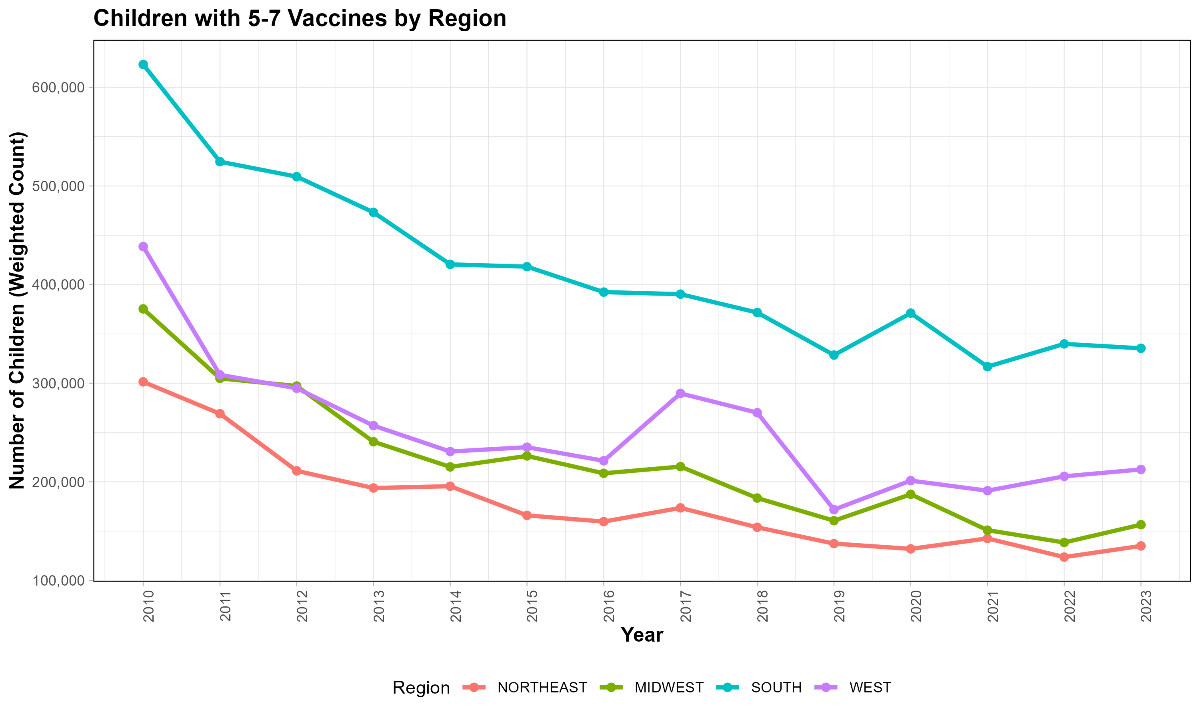


Figure S2: Weighted number of children receiving 1-4 vaccines by region from 2010 to 2023.


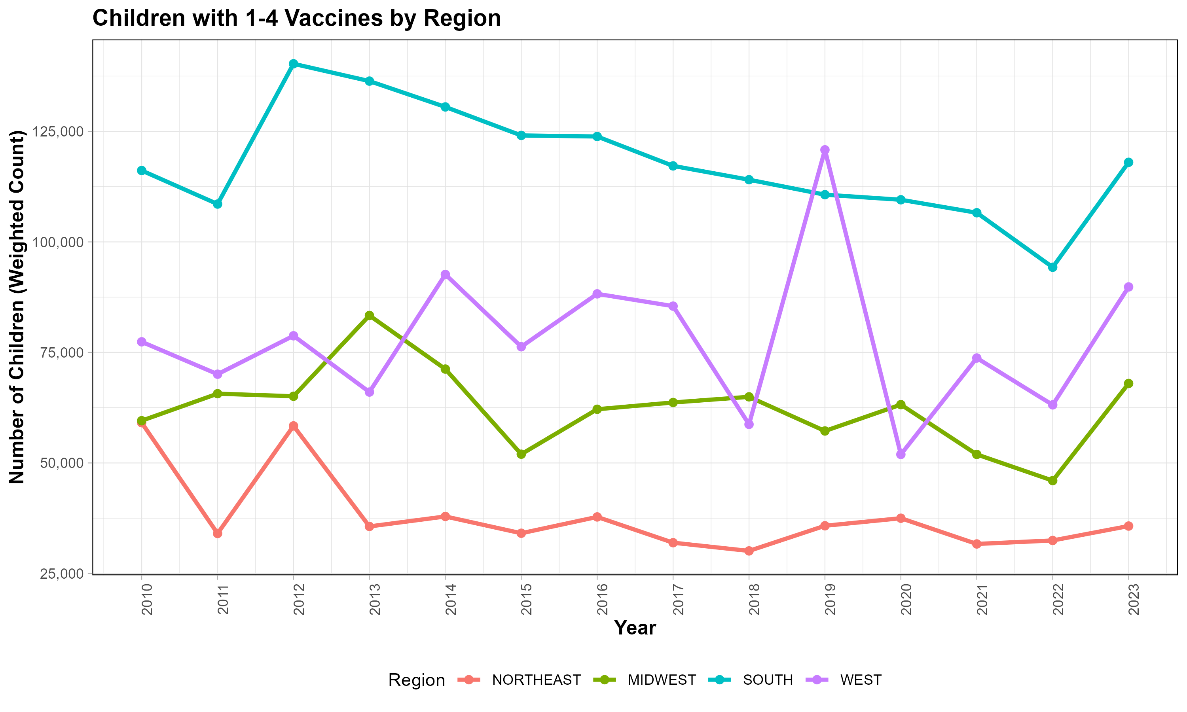


Figure S3: Weighted number of children receiving 5-7 vaccines by region from 2010 to 2023.


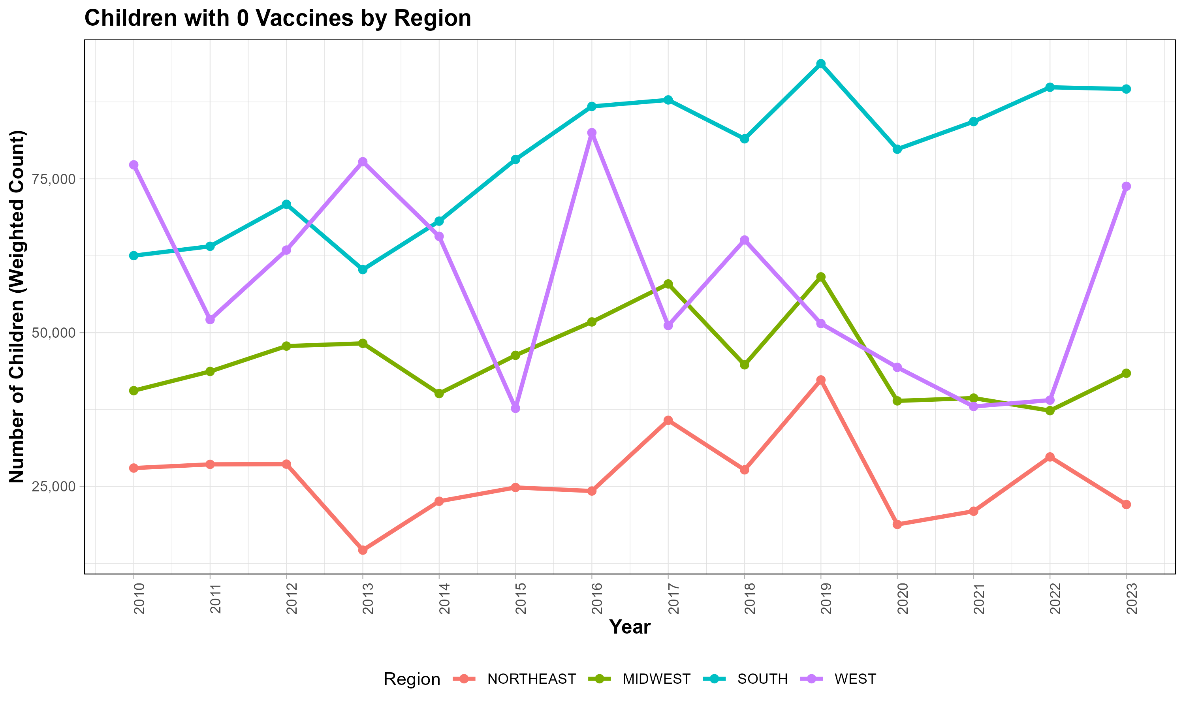


Figure S4: Weighted number of Children receiving 8 or more vaccines by region from 2010 to 2023.


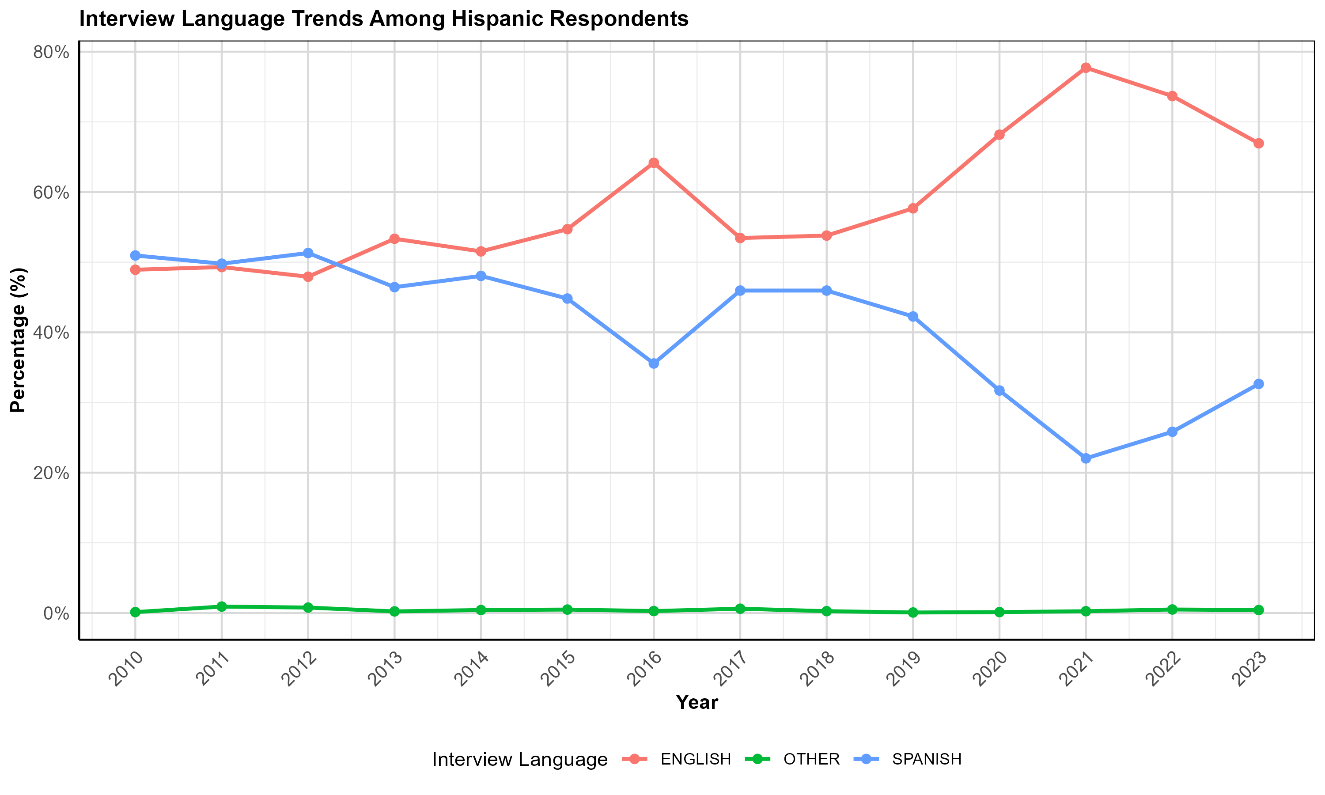


Figure S5: Trends in Spanish vs English interviews among Hispanic and non-Hispanic families.


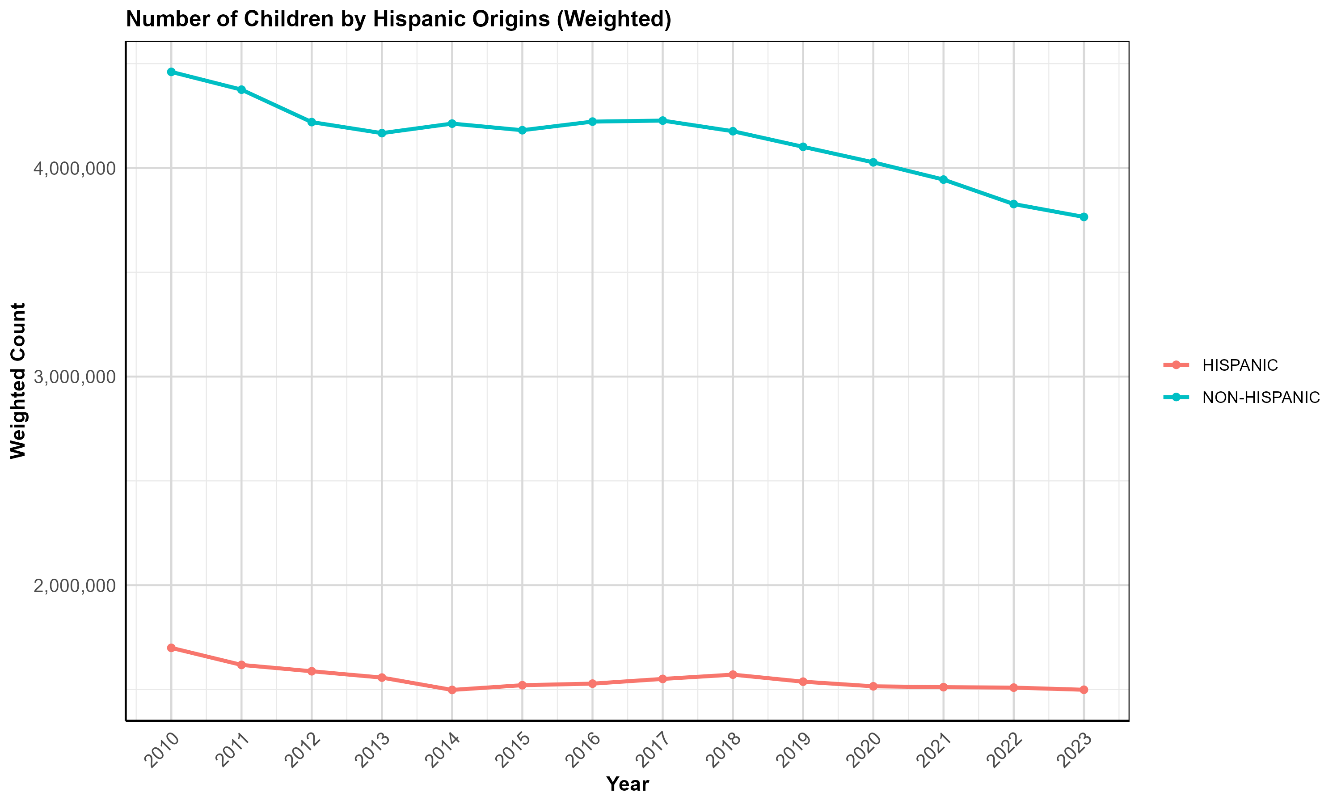


Figure S6: Weighted Hispanic to Non-Hispanic Origins.


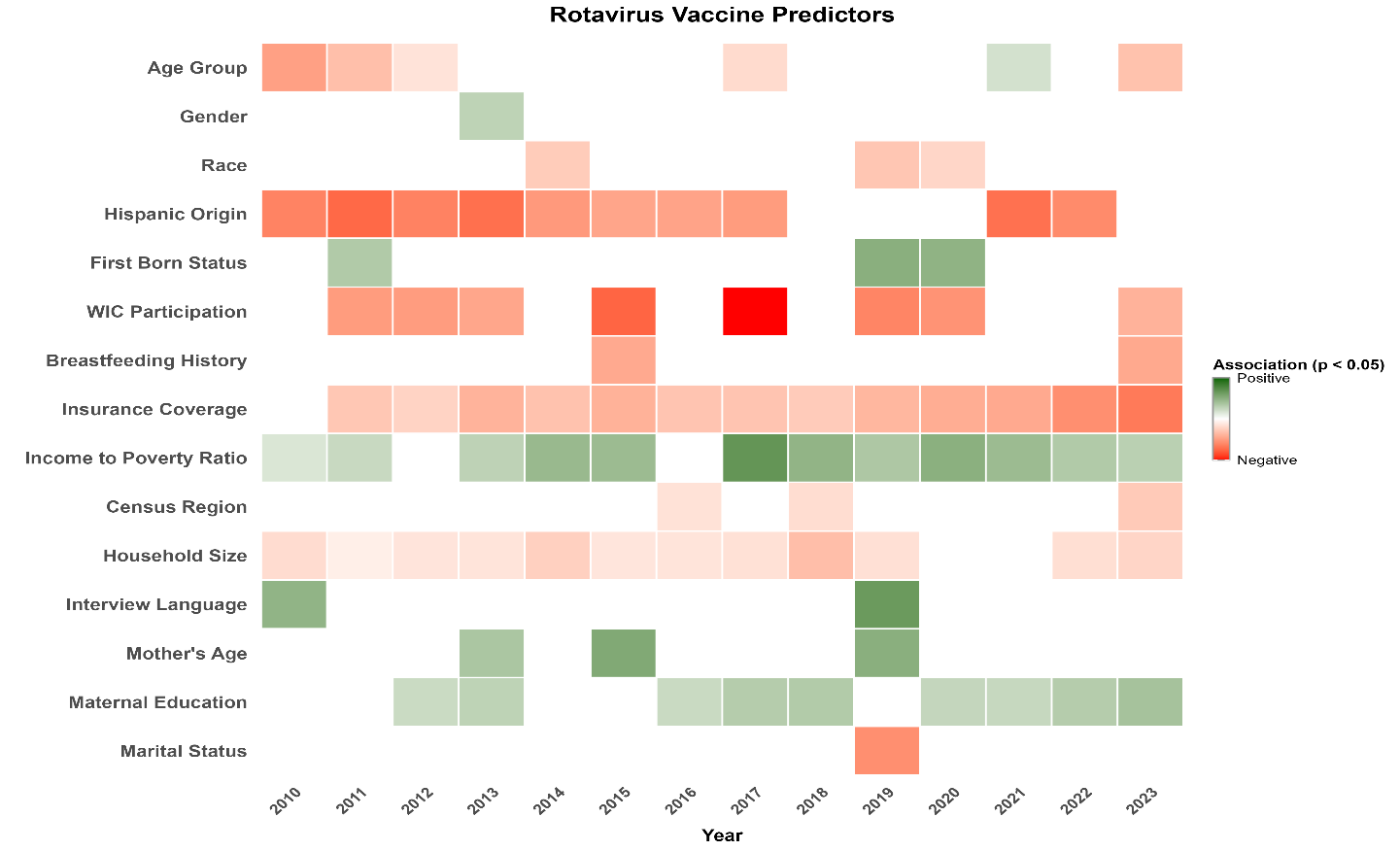


Figure S7: Heatmap of the positive and negative associations of SDoH on Rotavirus Vaccine across 2010-2023.


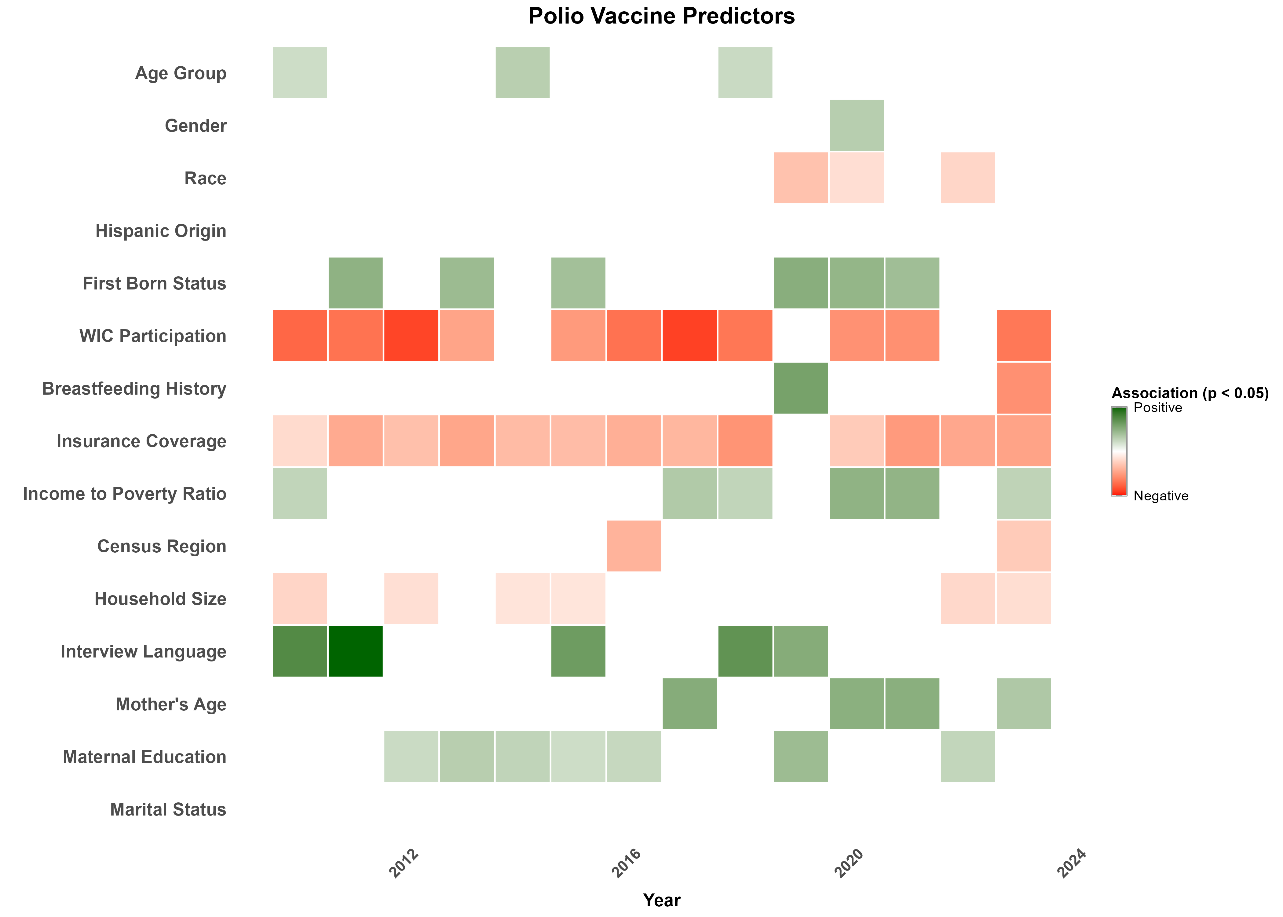


Figure S8: Heatmap of the positive and negative associations of SDoH on Polio Vaccine across 2010-2023.


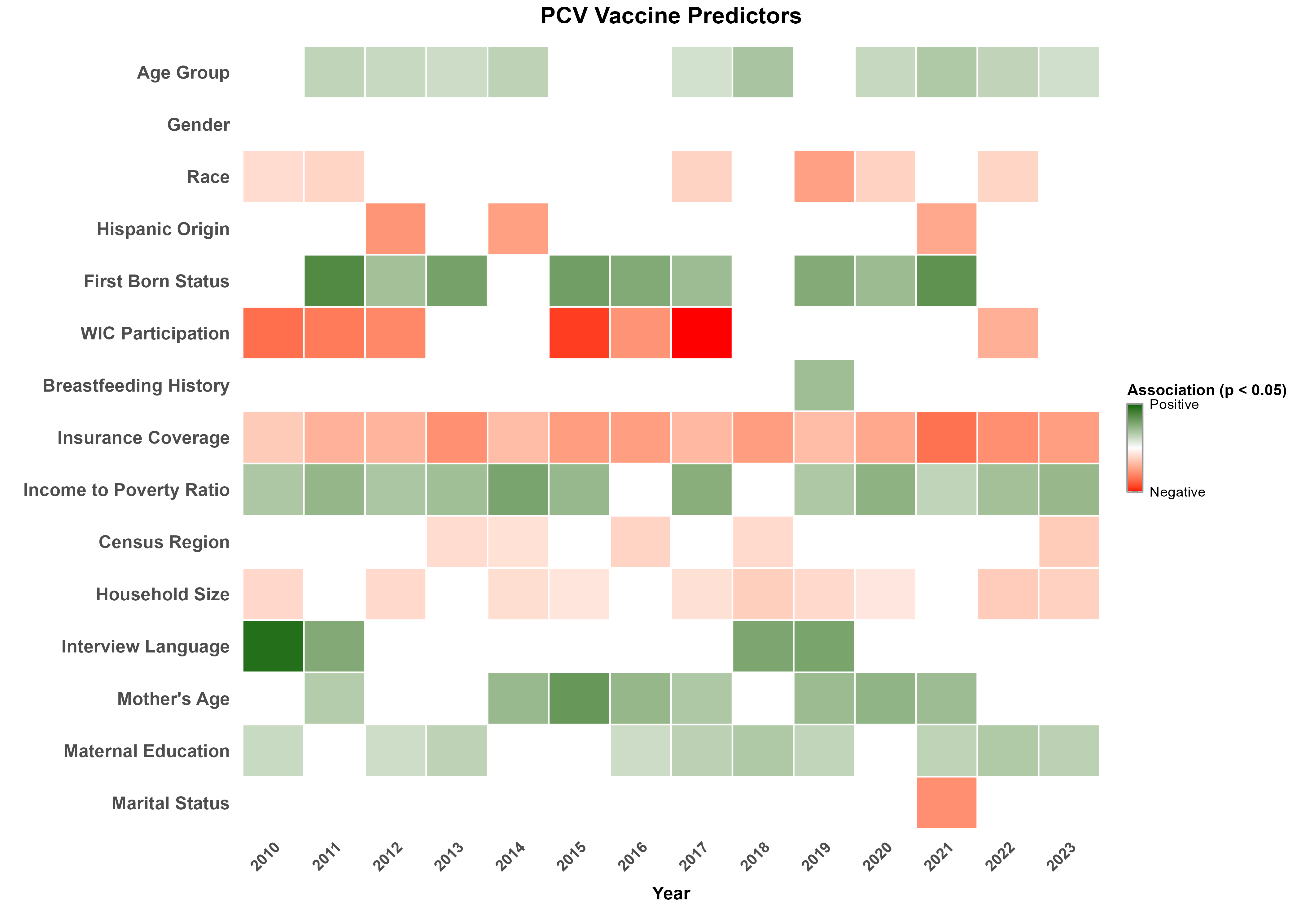


Figure S9: Heatmap of the positive and negative associations of SDoH on PCV Vaccine across 2010-2023.


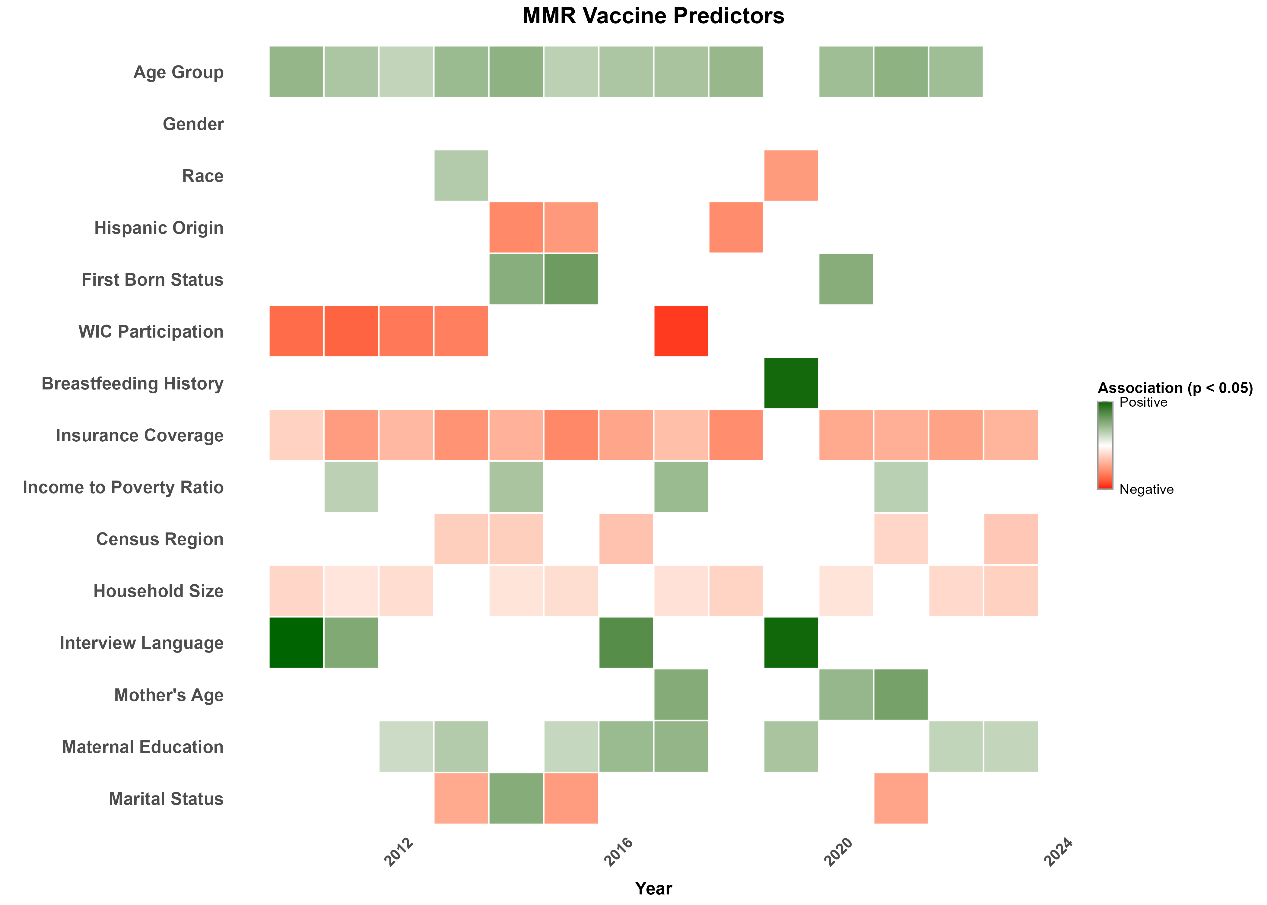


Figure S10: Heatmap of the positive and negative associations of SDoH on MMR Vaccine across 2010-2023.


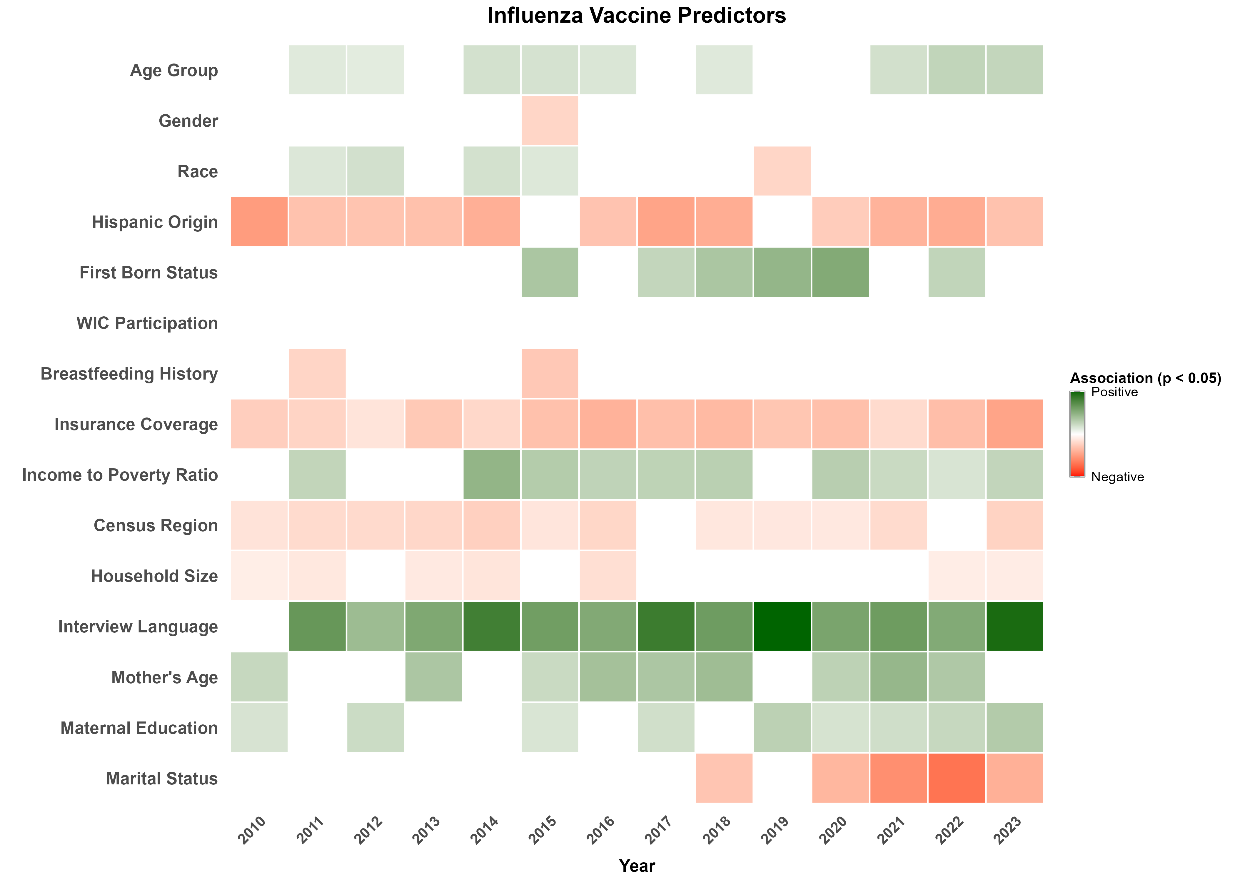


Figure S11: Heatmap of the positive and negative associations of SDoH on Influenza Vaccine across 2010-2023.


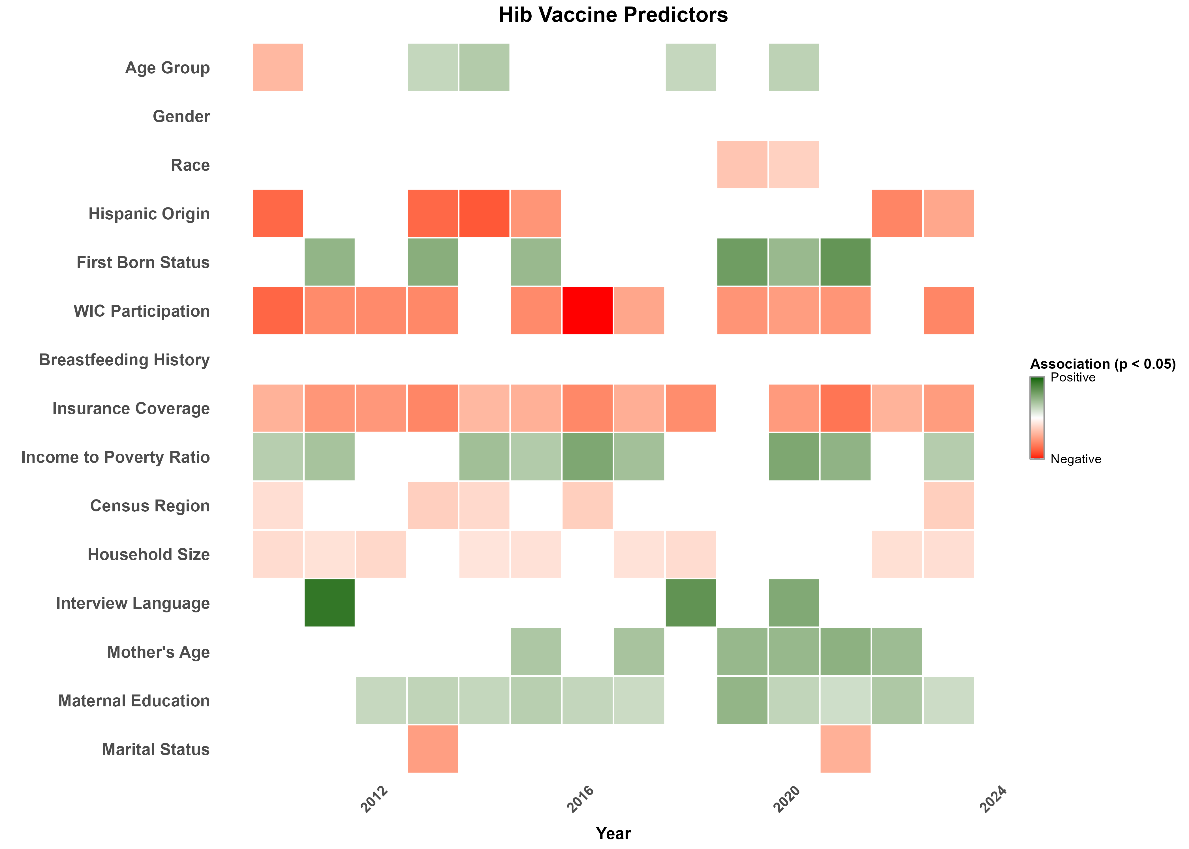


Figure S12: Heatmap of the positive and negative associations of SDoH on H1b Vaccine across 2010-2023.


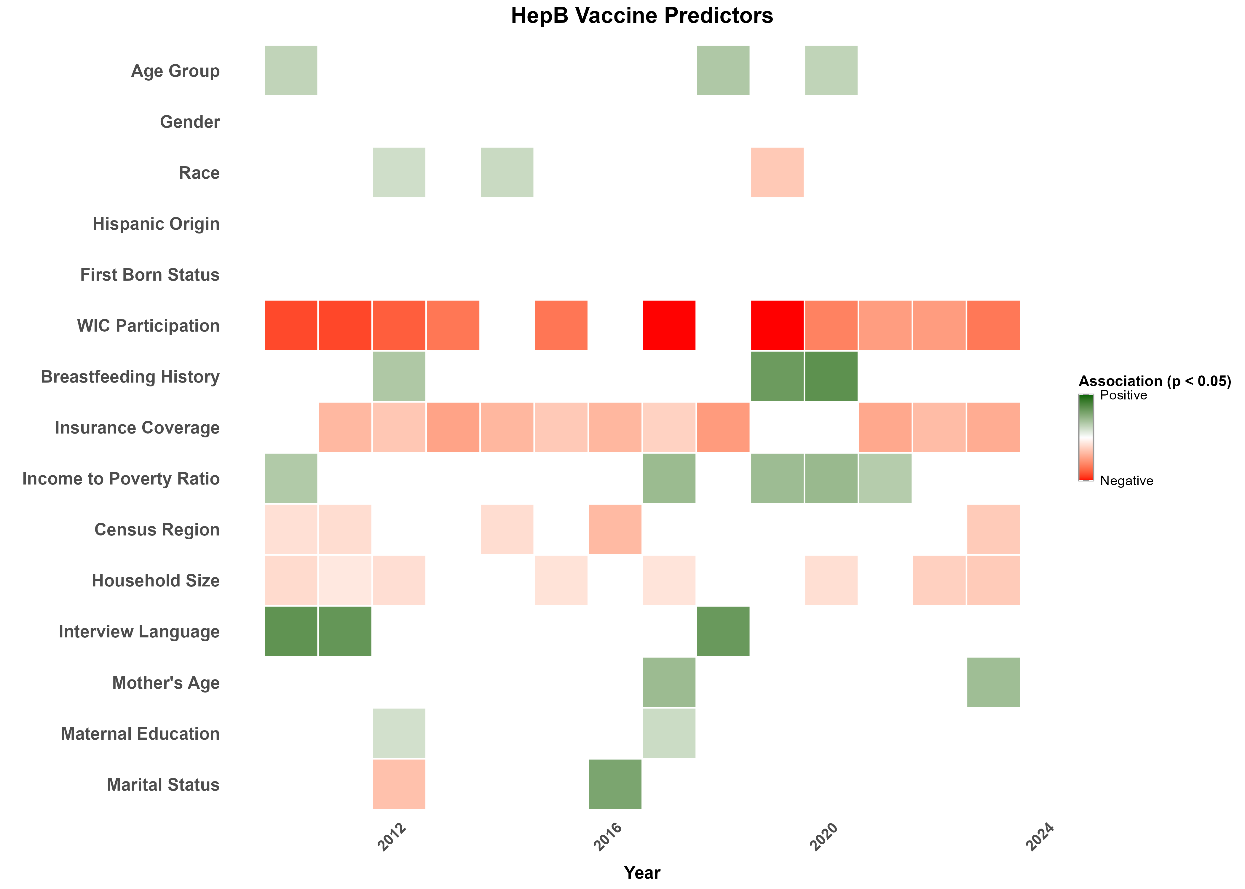


Figure S13: Heatmap of the positive and negative associations of SDoH on Hepatitis B Vaccine across 2010-2023


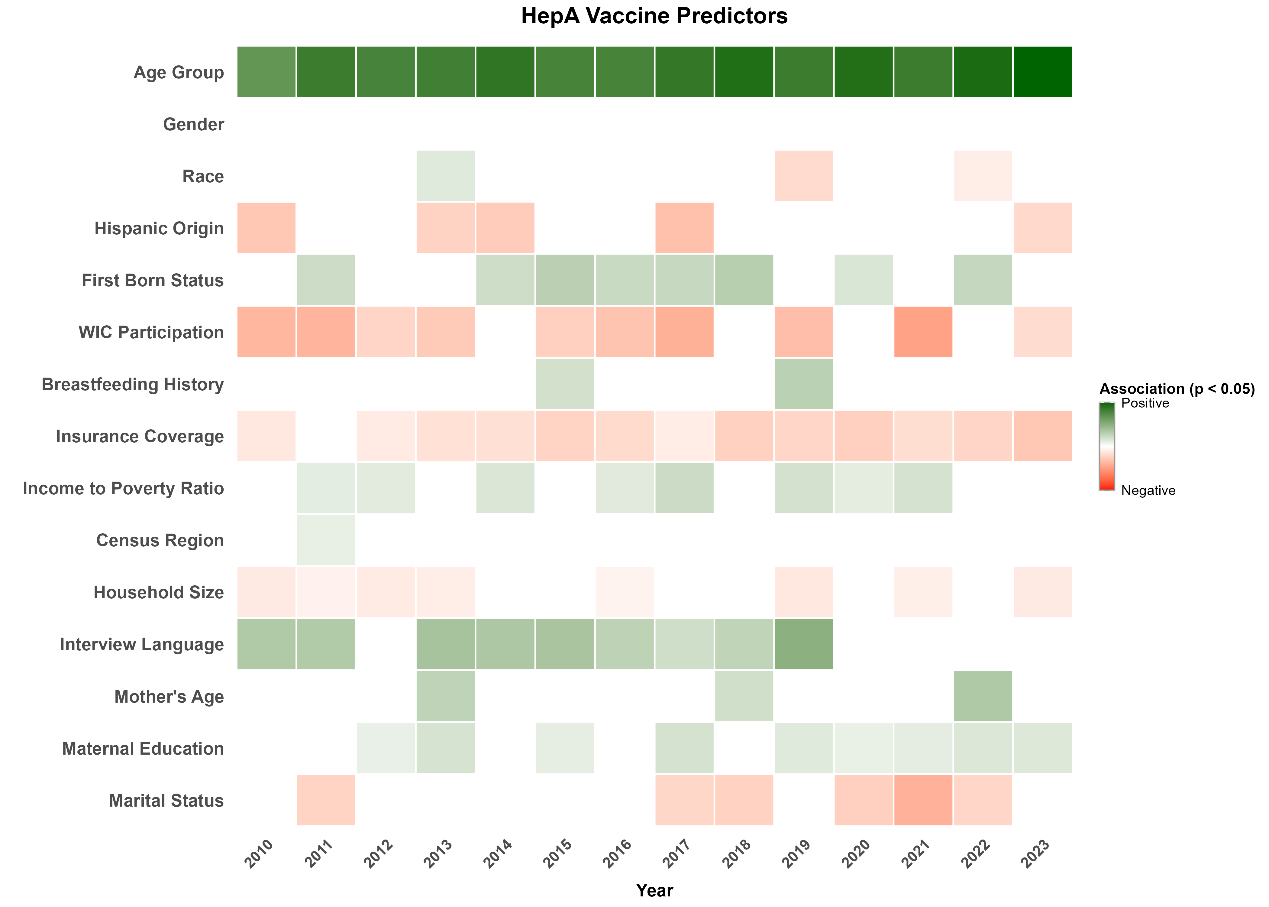


Figure S14: Heatmap of the positive and negative associations of SDoH on Hepatitis A Vaccine across 2010-2023.


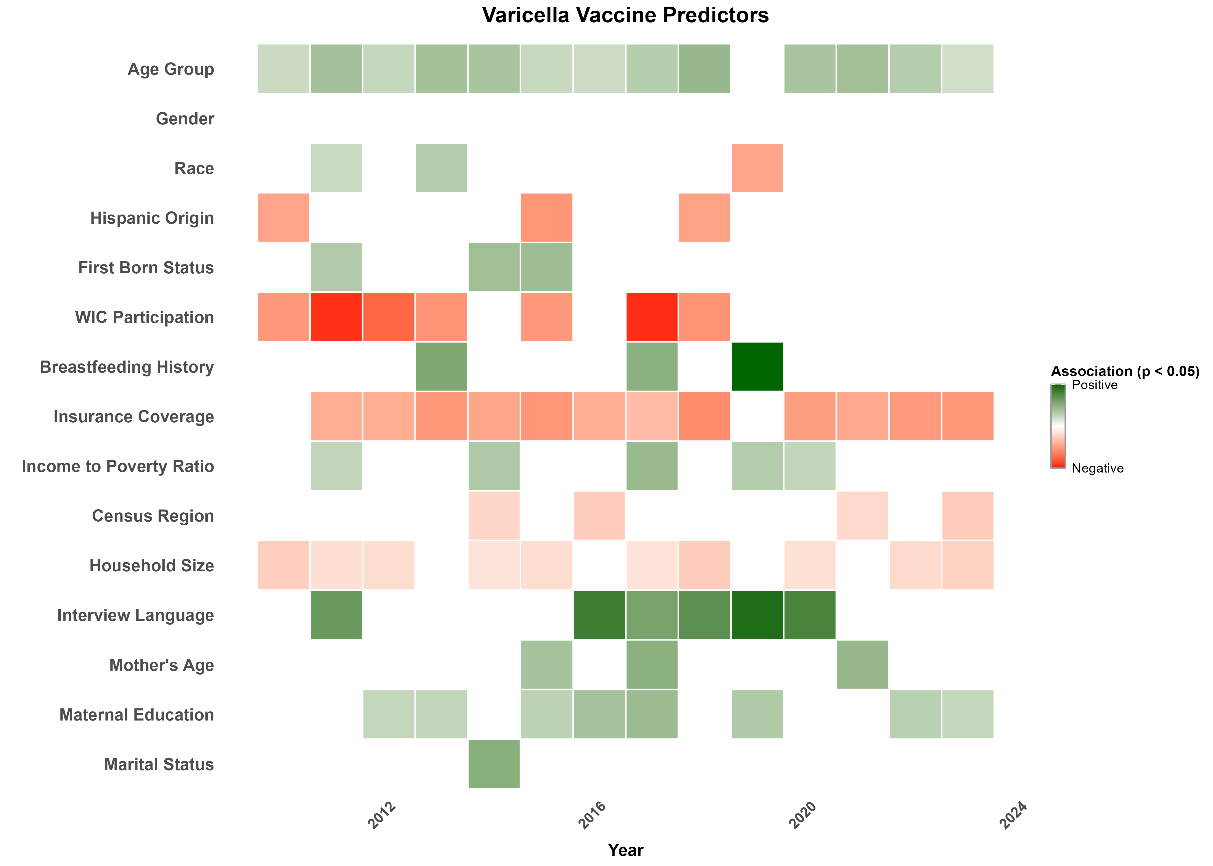


Figure S15: Heatmap of the positive and negative associations of SDoH on Varicella Vaccine across 2010-2023.


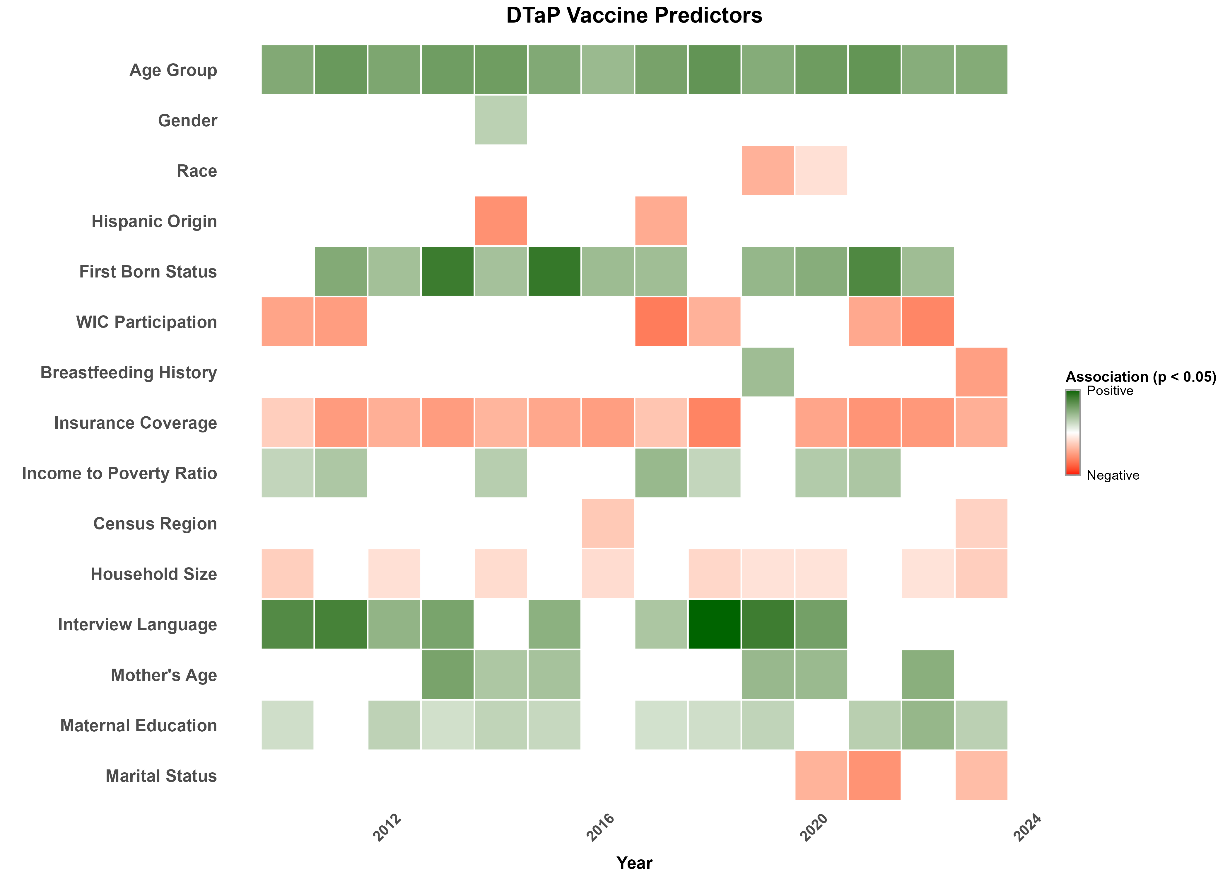


Figure S16: Heatmap of the positive and negative associations of SDoH on DTaP Vaccine across 2010-2023.


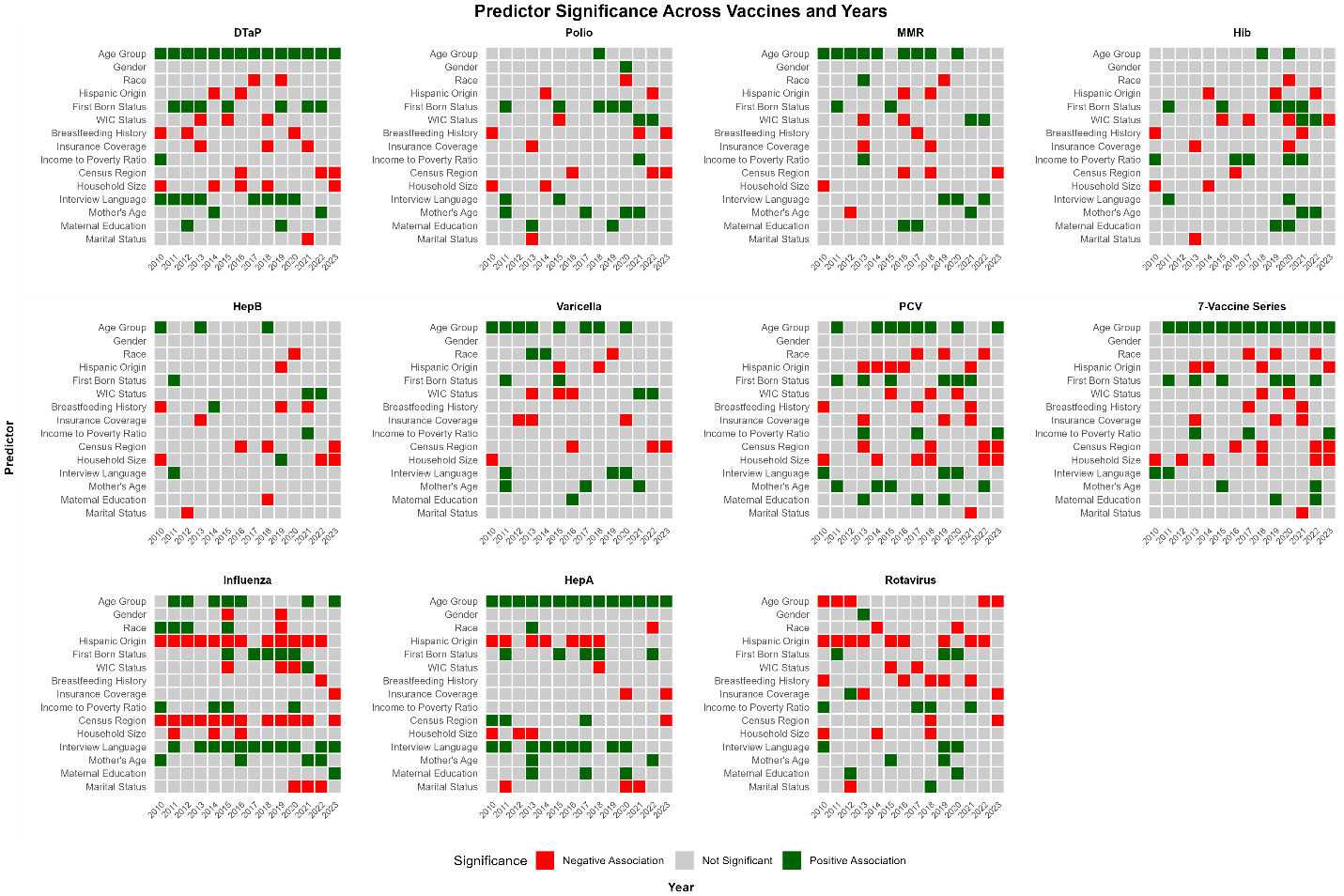


Figure S17: Heatmap of the positive and negative associations of SDoH on all 10 Vaccines.


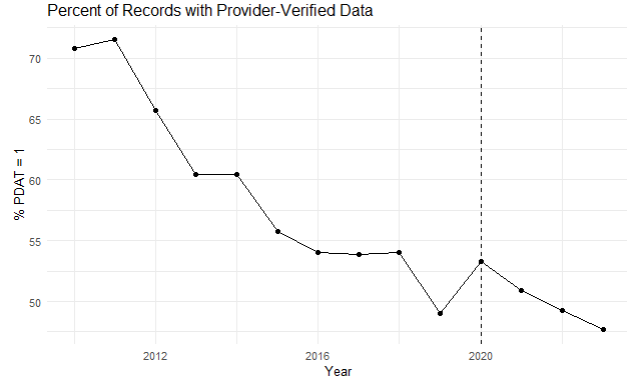


Figure S18. Percent of records with provider-verified data.

**S.4. SUBGROUP ANALYSES**

Subgroup interaction analyses were conducted to evaluate whether associations between key social determinants varied jointly across selected predictor pairs for completion of the combined 7-vaccine series. Wald tests were used to assess statistical evidence of interaction within each survey year. Interaction between insurance status and income-to-poverty ratio was observed intermittently across the study period, with statistically significant interaction effects in multiple pre-pandemic and late-period years. Similarly, interaction between Hispanic origin and language of interview demonstrated significant effects in several years prior to 2021, suggesting that language-related disparities may differentially affect Hispanic households across time.

In contrast, interaction between age group and maternal education showed limited and inconsistent statistical evidence across survey years. Interaction between census region and interview language was observed in selected years, particularly before and during the early pandemic period, indicating that language-related disparities may vary geographically. Overall, interaction effects were not uniformly persistent across all years, suggesting temporal variability in how structural and demographic factors jointly influenced vaccination completion.


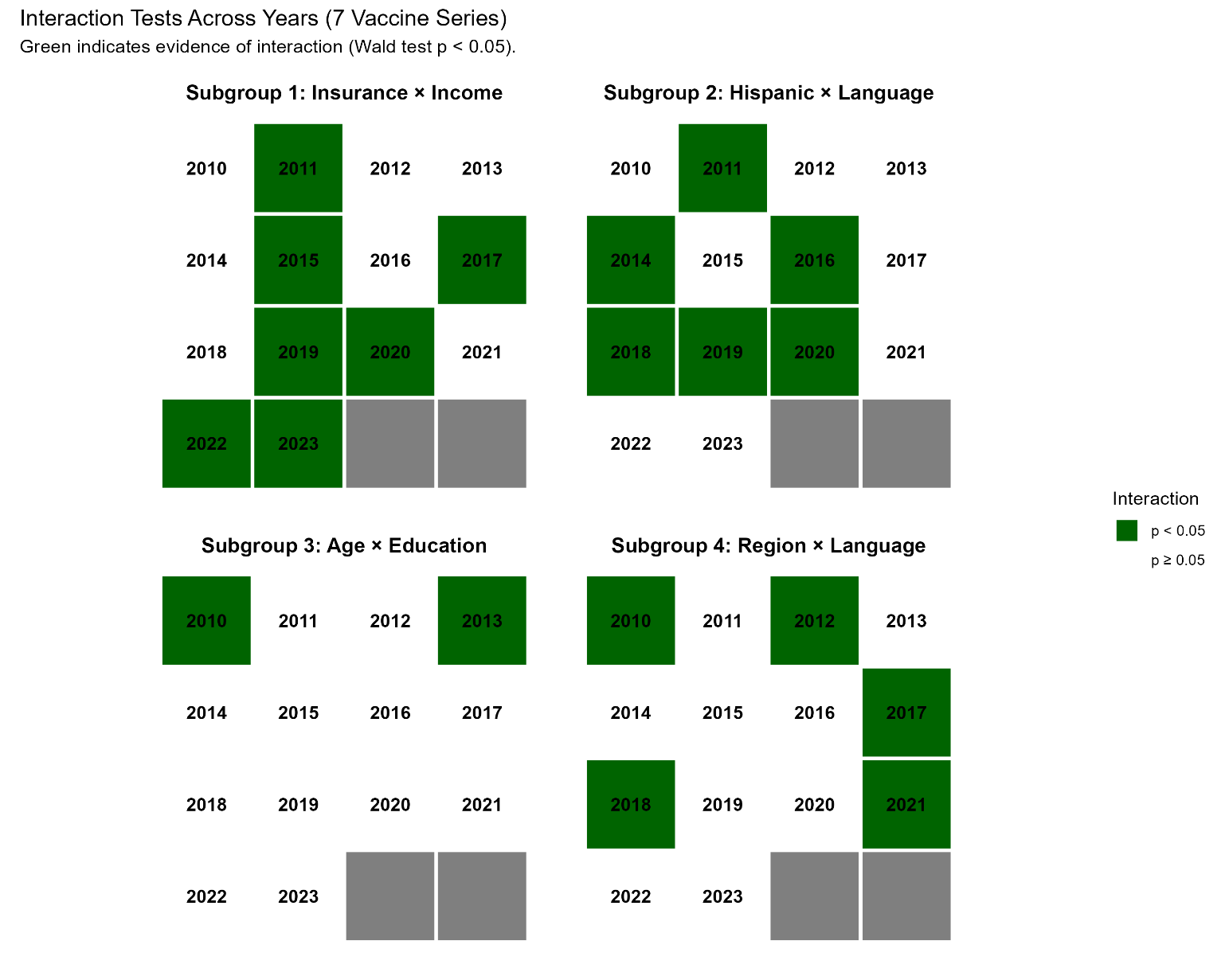


*Figure S19. Year-specific interaction tests for selected predictor pairs (Combined 7-Vaccine Series), 2010–2023*

**S.5. SENSITIVITY ANALYSES**

To evaluate robustness, two sensitivity analyses were conducted. First, models were re-estimated using the alternative measure of current WIC participation (Figure S19). This variable exhibited up to 60% missingness across survey years. Because survey-weighted logistic regression relies on complete case analysis, observations with missing WIC data were excluded, resulting in a substantially reduced analytic sample size. This reduction was associated with wider confidence intervals and instability in selected estimates, most notably for insurance coverage. The observed variability suggests that missingness in the current WIC measure may not have been random and likely influenced specific associations.

Second, we estimated a model excluding WIC status entirely (Figure S20). Results from this specification were highly consistent with the primary model presented in the main manuscript (Figure 7), which used the cumulative WIC measure. Associations for age group, maternal education, income-to-poverty ratio, region, language of interview, and other core predictors remained stable in both direction and magnitude. Apart from changes in insurance-related estimates when WIC was included, the broader pattern of social determinant associations was unchanged. These findings support the validity and robustness of the primary analytic approach.


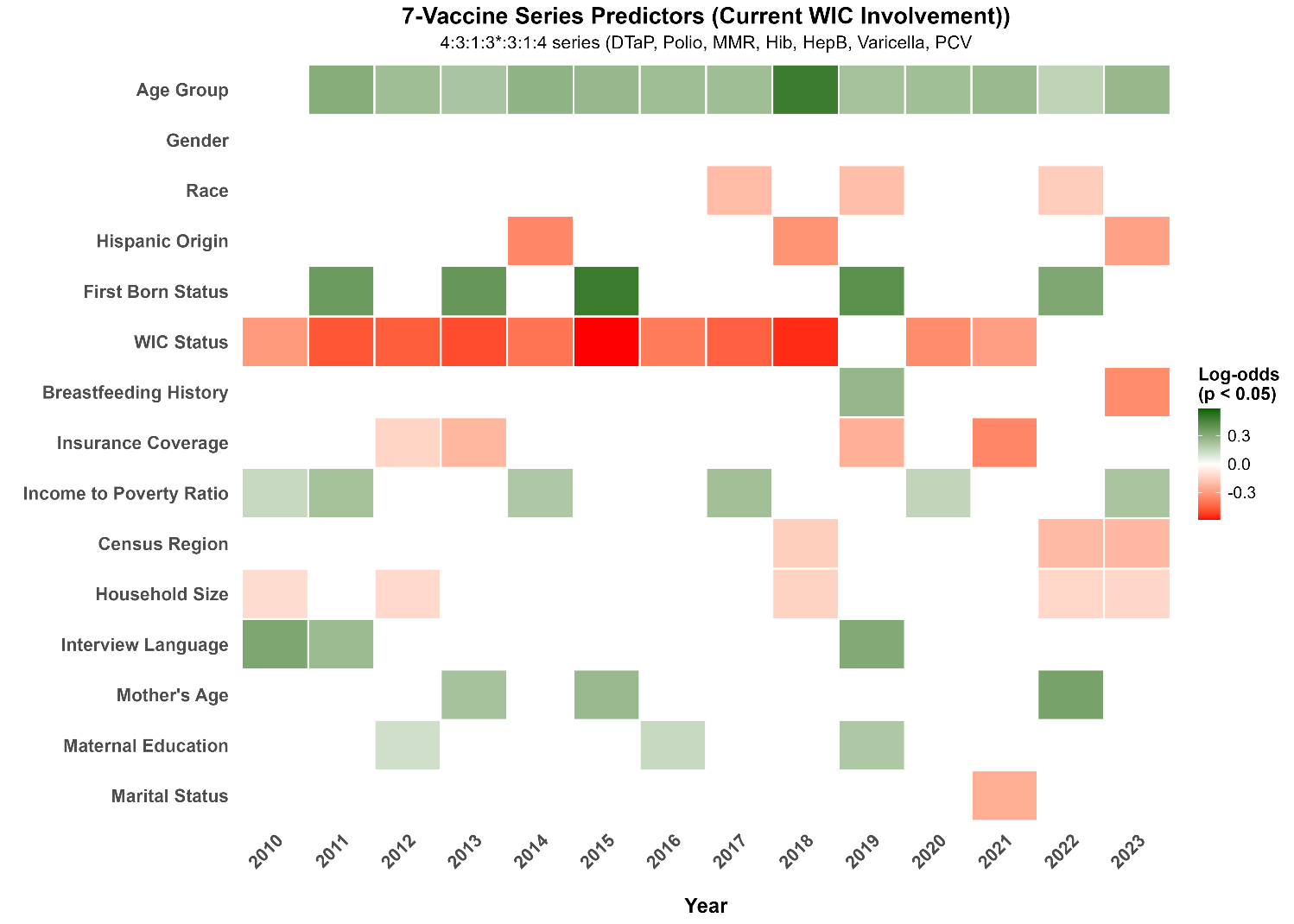


*Figure S20. Year-specific associations for the Combined 7-Vaccine Series using current WIC participation.*


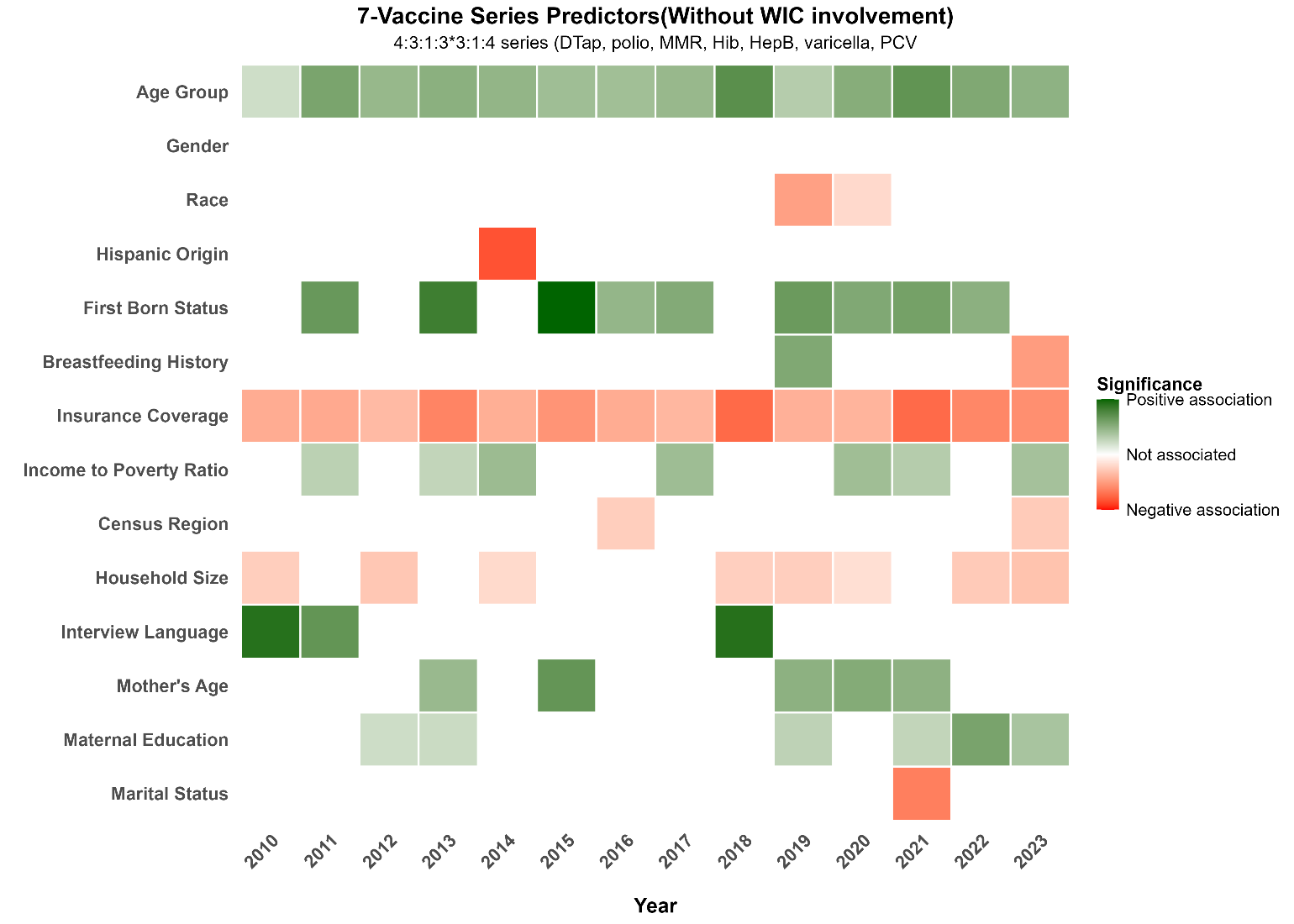


*Figure S21. Year-specific associations for the Combined 7-Vaccine Series excluding WIC status.*

# **SURVEY- WEIGHTED LOGISTIC REGRESSION MODEL RESULTS TABLES**

## **DTaP Vaccine Results 2010**

| **Predictor** | **Odds Ratio** | **95% CI** | **SE** | **P value** |
| --- | --- | --- | --- | --- |
| (Intercept) | 4.38 | (1.21, 15.83) | 0.655 | .024 |
| Age Group | 1.43 | (1.29, 1.59) | 0.054 | <.001 |
| Household size | 0.85 | (0.79, 0.90) | 0.034 | <.001 |
| Breastfeeding History | 0.87 | (0.72, 1.06) | 0.099 | .168 |
| WIC benefits | 0.73 | (0.55, 0.97) | 0.144 | .029 |
| Maternal Education | 1.15 | (1.04, 1.27) | 0.052 | .008 |
| Firstborn | 1.03 | (0.86, 1.24) | 0.094 | .751 |
| Hispanic Ethnicity | 0.97 | (0.75, 1.25) | 0.130 | .807 |
| Income | 1.19 | (1.03, 1.38) | 0.075 | .022 |
| Language | 1.64 | (1.22, 2.21) | 0.152 | .001 |
| Maternal Age | 1.01 | (0.85, 1.22) | 0.092 | .880 |
| Marital Status | 1.17 | (0.95, 1.45) | 0.109 | .144 |
| Racial Identity | 0.94 | (0.84, 1.06) | 0.060 | .311 |
| Sex | 1.09 | (0.92, 1.28) | 0.086 | .338 |
| Insurance Status | 0.84 | (0.75, 0.94) | 0.057 | .003 |
| Region | 0.91 | (0.82, 1.00) | 0.049 | .051 |

*Survey-weighted logistic regression estimates of factors associated with DTaP Vaccine, NIS-Child 2010*.

## **DTaP Vaccine Results 2011**

| Predictor | Odds Ratio | 95% CI | SE | P value |
| --- | --- | --- | --- | --- |
| (Intercept) | 1.49 | (0.45, 4.94) | 0.613 | .518 |
| Age Group | 1.54 | (1.39, 1.70) | 0.050 | <.001 |
| Household size | 0.94 | (0.88, 1.00) | 0.034 | .064 |
| Breastfeeding History | 0.88 | (0.72, 1.08) | 0.105 | .220 |
| WIC benefits | 0.72 | (0.54, 0.94) | 0.141 | .018 |
| Maternal Education | 1.03 | (0.93, 1.15) | 0.054 | .552 |
| Firstborn | 1.43 | (1.17, 1.74) | 0.101 | <.001 |
| Hispanic Ethnicity | 1.06 | (0.83, 1.35) | 0.124 | .628 |
| Income | 1.26 | (1.09, 1.46) | 0.075 | .002 |
| Language | 1.70 | (1.27, 2.28) | 0.149 | <.001 |
| Maternal Age | 1.15 | (0.97, 1.36) | 0.087 | .120 |
| Marital Status | 0.98 | (0.79, 1.22) | 0.110 | .855 |
| Racial Identity | 0.98 | (0.87, 1.11) | 0.064 | .793 |
| Sex | 1.02 | (0.87, 1.20) | 0.082 | .813 |
| Insurance Status | 0.71 | (0.63, 0.80) | 0.062 | <.001 |
| Region | 1.01 | (0.94, 1.10) | 0.041 | .720 |

*Survey-weighted logistic regression estimates of factors associated with DTaP Vaccine, NIS-Child 2011*.

## **DTaP Vaccine Results 2012**

| Predictor | Odds Ratio | 95% CI | SE | P value |
| --- | --- | --- | --- | --- |
| (Intercept) | 2.23 | (0.70, 7.13) | 0.592 | .175 |
| Age Group | 1.45 | (1.30, 1.62) | 0.056 | <.001 |
| Household size | 0.90 | (0.84, 0.96) | 0.033 | <.001 |
| Breastfeeding History | 1.07 | (0.88, 1.31) | 0.101 | .481 |
| WIC benefits | 0.77 | (0.58, 1.03) | 0.144 | .076 |
| Maternal Education | 1.20 | (1.09, 1.33) | 0.051 | <.001 |
| Firstborn | 1.30 | (1.04, 1.64) | 0.117 | .024 |
| Hispanic Ethnicity | 0.91 | (0.69, 1.20) | 0.140 | .501 |
| Income | 1.14 | (0.99, 1.32) | 0.073 | .069 |
| Language | 1.37 | (1.01, 1.85) | 0.153 | .041 |
| Maternal Age | 1.09 | (0.91, 1.30) | 0.090 | .335 |
| Marital Status | 0.93 | (0.75, 1.16) | 0.112 | .530 |
| Racial Identity | 1.08 | (0.97, 1.21) | 0.055 | .149 |
| Sex | 0.91 | (0.77, 1.07) | 0.086 | .257 |
| Insurance Status | 0.76 | (0.69, 0.84) | 0.050 | <.001 |
| Region | 0.97 | (0.89, 1.06) | 0.046 | .533 |

*Survey-weighted logistic regression estimates of factors associated with DTaP Vaccine, NIS-Child 2012*.

## **DTaP Vaccine Results 2013**

| Predictor | Odds Ratio | 95% CI | SE | P value |
| --- | --- | --- | --- | --- |
| (Intercept) | 0.58 | (0.14, 2.34) | 0.716 | .440 |
| Age Group | 1.51 | (1.35, 1.70) | 0.060 | <.001 |
| Household size | 0.96 | (0.88, 1.04) | 0.042 | .328 |
| Breastfeeding History | 1.09 | (0.86, 1.37) | 0.118 | .468 |
| WIC benefits | 0.92 | (0.68, 1.24) | 0.154 | .571 |
| Maternal Education | 1.14 | (1.01, 1.28) | 0.060 | .030 |
| Firstborn | 1.74 | (1.37, 2.21) | 0.121 | <.001 |
| Hispanic Ethnicity | 0.78 | (0.60, 1.03) | 0.139 | .081 |
| Income | 1.13 | (0.96, 1.33) | 0.083 | .131 |
| Language | 1.47 | (1.04, 2.08) | 0.177 | .030 |
| Maternal Age | 1.47 | (1.19, 1.82) | 0.108 | <.001 |
| Marital Status | 0.95 | (0.75, 1.20) | 0.119 | .646 |
| Racial Identity | 1.00 | (0.88, 1.13) | 0.064 | .975 |
| Sex | 1.14 | (0.95, 1.38) | 0.096 | .164 |
| Insurance Status | 0.71 | (0.63, 0.81) | 0.063 | <.001 |
| Region | 0.92 | (0.83, 1.01) | 0.049 | .072 |

*Survey-weighted logistic regression estimates of factors associated with DTaP Vaccine, NIS-Child 2013*.

## **DTaP Vaccine Results 2014**

| Predictor | Odds Ratio | 95% CI | SE | P value |
| --- | --- | --- | --- | --- |
| (Intercept) | 1.70 | (0.39, 7.51) | 0.758 | .483 |
| Age Group | 1.51 | (1.34, 1.71) | 0.062 | <.001 |
| Household size | 0.88 | (0.82, 0.95) | 0.038 | .001 |
| Breastfeeding History | 0.93 | (0.73, 1.17) | 0.119 | .535 |
| WIC benefits | 0.91 | (0.69, 1.21) | 0.145 | .521 |
| Maternal Education | 1.20 | (1.07, 1.34) | 0.059 | .002 |
| Firstborn | 1.29 | (1.03, 1.62) | 0.116 | .026 |
| Hispanic Ethnicity | 0.69 | (0.51, 0.93) | 0.155 | .015 |
| Income | 1.23 | (1.05, 1.44) | 0.081 | .012 |
| Language | 1.37 | (0.99, 1.89) | 0.165 | .056 |
| Maternal Age | 1.26 | (1.01, 1.59) | 0.115 | .042 |
| Marital Status | 1.10 | (0.86, 1.41) | 0.125 | .444 |
| Racial Identity | 0.93 | (0.82, 1.06) | 0.065 | .268 |
| Sex | 1.21 | (1.00, 1.46) | 0.096 | .045 |
| Insurance Status | 0.77 | (0.68, 0.89) | 0.070 | <.001 |
| Region | 0.94 | (0.86, 1.04) | 0.049 | .227 |

*Survey-weighted logistic regression estimates of factors associated with DTaP Vaccine, NIS-Child 2014*.

## **DTaP Vaccine Results 2015**

| Predictor | Odds Ratio | 95% CI | SE | P value |
| --- | --- | --- | --- | --- |
| (Intercept) | 1.22 | (0.25, 6.07) | 0.818 | .806 |
| Age Group | 1.44 | (1.28, 1.61) | 0.059 | <.001 |
| Household size | 0.97 | (0.90, 1.04) | 0.037 | .417 |
| Breastfeeding History | 1.01 | (0.82, 1.25) | 0.108 | .891 |
| WIC benefits | 0.76 | (0.53, 1.10) | 0.185 | .145 |
| Maternal Education | 1.18 | (1.05, 1.32) | 0.058 | .005 |
| Firstborn | 1.78 | (1.42, 2.22) | 0.115 | <.001 |
| Hispanic Ethnicity | 0.84 | (0.65, 1.09) | 0.134 | .198 |
| Income | 1.16 | (0.98, 1.38) | 0.086 | .078 |
| Language | 1.39 | (1.07, 1.80) | 0.132 | .013 |
| Maternal Age | 1.29 | (1.04, 1.60) | 0.110 | .020 |
| Marital Status | 0.82 | (0.64, 1.05) | 0.126 | .123 |
| Racial Identity | 1.03 | (0.92, 1.16) | 0.059 | .575 |
| Sex | 1.09 | (0.91, 1.31) | 0.093 | .349 |
| Insurance Status | 0.74 | (0.65, 0.84) | 0.062 | <.001 |
| Region | 0.92 | (0.83, 1.02) | 0.050 | .101 |

*Survey-weighted logistic regression estimates of factors associated with DTaP Vaccine, NIS-Child 2015*.

## **DTaP Vaccine Results 2016**

| Predictor | Odds Ratio | 95% CI | SE | P value |
| --- | --- | --- | --- | --- |
| (Intercept) | 4.07 | (1.11, 14.95) | 0.664 | .035 |
| Age Group | 1.34 | (1.19, 1.50) | 0.057 | <.001 |
| Household size | 0.89 | (0.82, 0.96) | 0.039 | .002 |
| Breastfeeding History | 0.97 | (0.76, 1.24) | 0.125 | .805 |
| WIC benefits | 0.91 | (0.63, 1.31) | 0.187 | .597 |
| Maternal Education | 1.13 | (0.99, 1.29) | 0.067 | .071 |
| Firstborn | 1.33 | (1.04, 1.69) | 0.125 | .024 |
| Hispanic Ethnicity | 0.86 | (0.64, 1.15) | 0.148 | .297 |
| Income | 1.12 | (0.94, 1.34) | 0.089 | .188 |
| Language | 1.33 | (0.98, 1.80) | 0.154 | .064 |
| Maternal Age | 1.19 | (0.95, 1.49) | 0.116 | .133 |
| Marital Status | 1.04 | (0.80, 1.34) | 0.133 | .784 |
| Racial Identity | 1.02 | (0.89, 1.16) | 0.067 | .814 |
| Sex | 1.06 | (0.87, 1.29) | 0.100 | .570 |
| Insurance Status | 0.72 | (0.61, 0.84) | 0.079 | <.001 |
| Region | 0.83 | (0.75, 0.92) | 0.052 | <.001 |

*Survey-weighted logistic regression estimates of factors associated with DTaP Vaccine, NIS-Child 2016*.

## **DTaP Vaccine Results 2017**

| Predictor | Odds Ratio | 95% CI | SE | P value |
| --- | --- | --- | --- | --- |
| (Intercept) | 4.80 | (1.27, 18.08) | 0.677 | .020 |
| Age Group | 1.48 | (1.32, 1.66) | 0.058 | <.001 |
| Household size | 0.97 | (0.90, 1.04) | 0.037 | .373 |
| Breastfeeding History | 1.00 | (0.80, 1.26) | 0.117 | .998 |
| WIC benefits | 0.65 | (0.47, 0.89) | 0.162 | .007 |
| Maternal Education | 1.13 | (1.01, 1.27) | 0.058 | .030 |
| Firstborn | 1.31 | (1.06, 1.63) | 0.111 | .014 |
| Hispanic Ethnicity | 0.75 | (0.59, 0.95) | 0.122 | .019 |
| Income | 1.34 | (1.15, 1.56) | 0.077 | <.001 |
| Language | 1.27 | (1.00, 1.61) | 0.121 | .050 |
| Maternal Age | 1.17 | (0.97, 1.41) | 0.095 | .098 |
| Marital Status | 0.77 | (0.59, 1.01) | 0.137 | .058 |
| Racial Identity | 0.92 | (0.82, 1.05) | 0.063 | .213 |
| Sex | 0.91 | (0.76, 1.08) | 0.090 | .278 |
| Insurance Status | 0.82 | (0.72, 0.93) | 0.066 | .002 |
| Region | 0.92 | (0.83, 1.02) | 0.051 | .095 |

*Survey-weighted logistic regression estimates of factors associated with DTaP Vaccine, NIS-Child 2017*.

## **DTaP Vaccine Results 2018**

| Predictor | Odds Ratio | 95% CI | SE | P value |
| --- | --- | --- | --- | --- |
| (Intercept) | 2.05 | (0.45, 9.34) | 0.774 | .354 |
| Age Group | 1.57 | (1.38, 1.78) | 0.064 | <.001 |
| Household size | 0.87 | (0.79, 0.96) | 0.050 | .006 |
| Breastfeeding History | 1.00 | (0.78, 1.28) | 0.128 | .985 |
| WIC benefits | 0.77 | (0.59, 0.99) | 0.132 | .043 |
| Maternal Education | 1.15 | (1.00, 1.31) | 0.068 | .043 |
| Firstborn | 1.28 | (0.97, 1.70) | 0.144 | .082 |
| Hispanic Ethnicity | 1.12 | (0.81, 1.54) | 0.163 | .490 |
| Income | 1.19 | (1.01, 1.39) | 0.079 | .032 |
| Language | 1.94 | (1.35, 2.80) | 0.186 | <.001 |
| Maternal Age | 1.21 | (0.98, 1.50) | 0.111 | .083 |
| Marital Status | 0.80 | (0.62, 1.03) | 0.129 | .085 |
| Racial Identity | 0.95 | (0.84, 1.07) | 0.063 | .401 |
| Sex | 1.07 | (0.88, 1.30) | 0.101 | .521 |
| Insurance Status | 0.66 | (0.59, 0.74) | 0.057 | <.001 |
| Region | 0.94 | (0.85, 1.05) | 0.054 | .289 |

*Survey-weighted logistic regression estimates of factors associated with DTaP Vaccine, NIS-Child 2018*.

## **DTaP Vaccine Results 2019**

| Predictor | Odds Ratio | 95% CI | SE | P value |
| --- | --- | --- | --- | --- |
| (Intercept) | 1.34 | (0.32, 5.58) | 0.726 | .684 |
| Age Group | 1.42 | (1.25, 1.61) | 0.065 | <.001 |
| Household size | 0.90 | (0.83, 0.98) | 0.043 | .019 |
| Breastfeeding History | 1.32 | (1.02, 1.69) | 0.129 | .033 |
| WIC benefits | 0.83 | (0.63, 1.11) | 0.146 | .212 |
| Maternal Education | 1.20 | (1.04, 1.37) | 0.071 | .012 |
| Firstborn | 1.35 | (1.02, 1.79) | 0.143 | .034 |
| Hispanic Ethnicity | 1.09 | (0.79, 1.49) | 0.160 | .604 |
| Income | 1.15 | (0.99, 1.33) | 0.075 | .060 |
| Language | 1.74 | (1.26, 2.38) | 0.162 | <.001 |
| Maternal Age | 1.34 | (1.07, 1.68) | 0.114 | .010 |
| Marital Status | 0.84 | (0.64, 1.10) | 0.138 | .212 |
| Racial Identity | 0.77 | (0.66, 0.89) | 0.077 | <.001 |
| Sex | 0.83 | (0.67, 1.02) | 0.106 | .075 |
| Insurance Status | 0.88 | (0.76, 1.01) | 0.072 | .078 |
| Region | 0.93 | (0.83, 1.05) | 0.061 | .231 |

*Survey-weighted logistic regression estimates of factors associated with DTaP Vaccine, NIS-Child 2019*.

## **DTaP Vaccine Results 2020**

| Predictor | Odds Ratio | 95% CI | SE | P value |
| --- | --- | --- | --- | --- |
| (Intercept) | 1.76 | (0.51, 6.04) | 0.629 | .367 |
| Age Group | 1.52 | (1.38, 1.68) | 0.050 | <.001 |
| Household size | 0.91 | (0.85, 0.97) | 0.036 | .006 |
| Breastfeeding History | 0.93 | (0.75, 1.16) | 0.112 | .508 |
| WIC benefits | 0.82 | (0.65, 1.03) | 0.118 | .094 |
| Maternal Education | 1.10 | (0.99, 1.22) | 0.052 | .067 |
| Firstborn | 1.41 | (1.15, 1.74) | 0.106 | .001 |
| Hispanic Ethnicity | 1.06 | (0.86, 1.31) | 0.107 | .556 |
| Income | 1.24 | (1.10, 1.41) | 0.064 | <.001 |
| Language | 1.49 | (1.10, 2.02) | 0.154 | .010 |
| Maternal Age | 1.34 | (1.10, 1.63) | 0.101 | .004 |
| Marital Status | 0.77 | (0.63, 0.94) | 0.100 | .009 |
| Racial Identity | 0.90 | (0.82, 0.99) | 0.049 | .025 |
| Sex | 1.00 | (0.85, 1.17) | 0.080 | .985 |
| Insurance Status | 0.73 | (0.65, 0.83) | 0.059 | <.001 |
| Region | 1.04 | (0.96, 1.12) | 0.040 | .356 |

*Survey-weighted logistic regression estimates of factors associated with DTaP Vaccine, NIS-Child 2020*.

## **DTaP Vaccine Results 2021**

| Predictor | Odds Ratio | 95% CI | SE | P value |
| --- | --- | --- | --- | --- |
| (Intercept) | 2.43 | (0.61, 9.69) | 0.706 | .209 |
| Age Group | 1.57 | (1.40, 1.76) | 0.058 | <.001 |
| Household size | 0.97 | (0.90, 1.05) | 0.039 | .407 |
| Breastfeeding History | 1.02 | (0.78, 1.35) | 0.140 | .867 |
| WIC benefits | 0.74 | (0.58, 0.95) | 0.124 | .016 |
| Maternal Education | 1.22 | (1.09, 1.37) | 0.058 | <.001 |
| Firstborn | 1.66 | (1.32, 2.08) | 0.116 | <.001 |
| Hispanic Ethnicity | 0.79 | (0.62, 1.00) | 0.122 | .053 |
| Income | 1.27 | (1.11, 1.45) | 0.070 | <.001 |
| Language | 1.45 | (0.95, 2.22) | 0.217 | .086 |
| Maternal Age | 1.22 | (0.99, 1.50) | 0.105 | .058 |
| Marital Status | 0.69 | (0.56, 0.86) | 0.110 | <.001 |
| Racial Identity | 0.96 | (0.86, 1.07) | 0.057 | .482 |
| Sex | 0.97 | (0.81, 1.16) | 0.091 | .765 |
| Insurance Status | 0.70 | (0.60, 0.81) | 0.077 | <.001 |
| Region | 0.92 | (0.84, 1.01) | 0.047 | .066 |

*Survey-weighted logistic regression estimates of factors associated with DTaP Vaccine, NIS-Child 2021*.

## **DTaP Vaccine Results 2022**

| Predictor | Odds Ratio | 95% CI | SE | P value |
| --- | --- | --- | --- | --- |
| (Intercept) | 3.52 | (0.84, 14.69) | 0.728 | .084 |
| Age Group | 1.41 | (1.26, 1.58) | 0.059 | <.001 |
| Household size | 0.91 | (0.84, 0.98) | 0.040 | .016 |
| Breastfeeding History | 0.97 | (0.75, 1.26) | 0.134 | .815 |
| WIC benefits | 0.66 | (0.51, 0.86) | 0.133 | .002 |
| Maternal Education | 1.35 | (1.20, 1.52) | 0.060 | <.001 |
| Firstborn | 1.32 | (1.04, 1.67) | 0.120 | .022 |
| Hispanic Ethnicity | 0.86 | (0.66, 1.10) | 0.129 | .226 |
| Income | 1.13 | (0.96, 1.34) | 0.085 | .148 |
| Language | 1.36 | (0.96, 1.93) | 0.180 | .088 |
| Maternal Age | 1.40 | (1.13, 1.73) | 0.108 | .002 |
| Marital Status | 0.88 | (0.69, 1.11) | 0.119 | .268 |
| Racial Identity | 0.92 | (0.81, 1.03) | 0.060 | .142 |
| Sex | 0.94 | (0.78, 1.13) | 0.094 | .490 |
| Insurance Status | 0.70 | (0.61, 0.81) | 0.073 | <.001 |
| Region | 0.94 | (0.86, 1.02) | 0.046 | .149 |

*Survey-weighted logistic regression estimates of factors associated with DTaP Vaccine, NIS-Child 2022*.

## **DTaP Vaccine Results 2023**

| Predictor | Odds Ratio | 95% CI | SE | P value |
| --- | --- | --- | --- | --- |
| (Intercept) | 12.40 | (3.82, 40.21) | 0.600 | <.001 |
| Age Group | 1.43 | (1.28, 1.59) | 0.054 | <.001 |
| Household size | 0.84 | (0.78, 0.92) | 0.041 | <.001 |
| Breastfeeding History | 0.72 | (0.56, 0.92) | 0.125 | .009 |
| WIC benefits | 0.81 | (0.66, 1.00) | 0.107 | .052 |
| Maternal Education | 1.22 | (1.09, 1.35) | 0.054 | <.001 |
| Firstborn | 1.08 | (0.88, 1.33) | 0.107 | .475 |
| Hispanic Ethnicity | 0.88 | (0.70, 1.10) | 0.114 | .257 |
| Income | 1.14 | (1.00, 1.31) | 0.071 | .056 |
| Language | 1.13 | (0.84, 1.51) | 0.149 | .429 |
| Maternal Age | 1.17 | (0.96, 1.44) | 0.104 | .123 |
| Marital Status | 0.79 | (0.65, 0.98) | 0.106 | .030 |
| Racial Identity | 0.98 | (0.88, 1.08) | 0.052 | .662 |
| Sex | 1.01 | (0.85, 1.19) | 0.087 | .953 |
| Insurance Status | 0.76 | (0.67, 0.87) | 0.068 | <.001 |
| Region | 0.86 | (0.78, 0.94) | 0.046 | <.001 |

*Survey-weighted logistic regression estimates of factors associated with DTaP Vaccine, NIS-Child 2023*.

## **HepA Vaccine Results 2010**

| Predictor | Odds Ratio | 95% CI | SE | P value |
| --- | --- | --- | --- | --- |
| (Intercept) | 0.72 | (0.27, 1.90) | 0.494 | .510 |
| Age Group | 1.94 | (1.80, 2.09) | 0.038 | <.001 |
| Household size | 0.90 | (0.85, 0.95) | 0.027 | <.001 |
| Breastfeeding History | 0.96 | (0.83, 1.10) | 0.073 | .547 |
| WIC benefits | 0.69 | (0.56, 0.86) | 0.108 | <.001 |
| Maternal Education | 1.06 | (0.98, 1.14) | 0.039 | .162 |
| Firstborn | 1.09 | (0.95, 1.25) | 0.070 | .207 |
| Hispanic Ethnicity | 0.75 | (0.64, 0.89) | 0.086 | .001 |
| Income | 1.05 | (0.95, 1.16) | 0.051 | .376 |
| Language | 1.40 | (1.13, 1.73) | 0.109 | .002 |
| Maternal Age | 0.98 | (0.86, 1.13) | 0.070 | .801 |
| Marital Status | 0.91 | (0.77, 1.08) | 0.086 | .263 |
| Racial Identity | 1.04 | (0.95, 1.13) | 0.044 | .378 |
| Sex | 0.97 | (0.86, 1.09) | 0.059 | .575 |
| Insurance Status | 0.89 | (0.81, 0.98) | 0.049 | .019 |
| Region | 1.05 | (0.99, 1.12) | 0.030 | .080 |

*Survey-weighted logistic regression estimates of factors associated with HepA vaccine coverage, NIS-Child 2010*.

## **HepA Vaccine Results 2011**

| Predictor | Odds Ratio | 95% CI | SE | P value |
| --- | --- | --- | --- | --- |
| (Intercept) | 0.25 | (0.11, 0.60) | 0.445 | .002 |
| Age Group | 2.27 | (2.12, 2.44) | 0.036 | <.001 |
| Household size | 0.94 | (0.89, 0.99) | 0.026 | .013 |
| Breastfeeding History | 0.91 | (0.79, 1.05) | 0.074 | .202 |
| WIC benefits | 0.68 | (0.57, 0.83) | 0.096 | <.001 |
| Maternal Education | 1.01 | (0.94, 1.09) | 0.038 | .815 |
| Firstborn | 1.24 | (1.07, 1.42) | 0.072 | .003 |
| Hispanic Ethnicity | 0.86 | (0.73, 1.01) | 0.083 | .069 |
| Income | 1.12 | (1.01, 1.24) | 0.052 | .032 |
| Language | 1.39 | (1.17, 1.65) | 0.089 | <.001 |
| Maternal Age | 1.09 | (0.96, 1.23) | 0.064 | .197 |
| Marital Status | 0.80 | (0.69, 0.94) | 0.080 | .006 |
| Racial Identity | 1.04 | (0.95, 1.14) | 0.045 | .361 |
| Sex | 1.02 | (0.91, 1.14) | 0.058 | .721 |
| Insurance Status | 0.92 | (0.84, 1.00) | 0.045 | .061 |
| Region | 1.10 | (1.04, 1.17) | 0.030 | .001 |

*Survey-weighted logistic regression estimates of factors associated with HepA vaccine coverage, NIS-Child 2011*.

## **HepA Vaccine Results 2012**

| Predictor | Odds Ratio | 95% CI | SE | P value |
| --- | --- | --- | --- | --- |
| (Intercept) | 0.24 | (0.10, 0.62) | 0.477 | .003 |
| Age Group | 2.18 | (2.01, 2.36) | 0.041 | <.001 |
| Household size | 0.90 | (0.85, 0.95) | 0.029 | <.001 |
| Breastfeeding History | 1.04 | (0.88, 1.22) | 0.082 | .652 |
| WIC benefits | 0.81 | (0.67, 0.97) | 0.096 | .025 |
| Maternal Education | 1.10 | (1.00, 1.20) | 0.045 | .047 |
| Firstborn | 1.13 | (0.96, 1.34) | 0.086 | .141 |
| Hispanic Ethnicity | 0.85 | (0.71, 1.02) | 0.092 | .080 |
| Income | 1.13 | (1.02, 1.25) | 0.052 | .020 |
| Language | 1.16 | (0.95, 1.42) | 0.102 | .138 |
| Maternal Age | 0.98 | (0.86, 1.13) | 0.071 | .809 |
| Marital Status | 1.01 | (0.86, 1.20) | 0.086 | .866 |
| Racial Identity | 1.02 | (0.93, 1.12) | 0.048 | .666 |
| Sex | 1.12 | (0.98, 1.27) | 0.065 | .087 |
| Insurance Status | 0.90 | (0.82, 0.98) | 0.045 | .020 |
| Region | 1.05 | (0.98, 1.13) | 0.035 | .140 |

*Survey-weighted logistic regression estimates of factors associated with HepA vaccine coverage, NIS-Child 2012*.

## **HepA Vaccine Results 2013**

| Predictor | Odds Ratio | 95% CI | SE | P value |
| --- | --- | --- | --- | --- |
| (Intercept) | 0.18 | (0.06, 0.54) | 0.558 | .002 |
| Age Group | 2.23 | (2.04, 2.43) | 0.045 | <.001 |
| Household size | 0.91 | (0.85, 0.98) | 0.034 | .008 |
| Breastfeeding History | 1.05 | (0.87, 1.27) | 0.097 | .616 |
| WIC benefits | 0.77 | (0.61, 0.96) | 0.113 | .018 |
| Maternal Education | 1.19 | (1.09, 1.30) | 0.046 | <.001 |
| Firstborn | 1.17 | (0.97, 1.40) | 0.093 | .092 |
| Hispanic Ethnicity | 0.80 | (0.65, 0.99) | 0.108 | .037 |
| Income | 0.96 | (0.86, 1.08) | 0.060 | .528 |
| Language | 1.45 | (1.13, 1.86) | 0.128 | .004 |
| Maternal Age | 1.31 | (1.12, 1.53) | 0.079 | <.001 |
| Marital Status | 0.84 | (0.69, 1.01) | 0.097 | .066 |
| Racial Identity | 1.14 | (1.03, 1.26) | 0.051 | .010 |
| Sex | 0.95 | (0.82, 1.09) | 0.072 | .457 |
| Insurance Status | 0.86 | (0.78, 0.95) | 0.050 | .002 |
| Region | 1.02 | (0.95, 1.10) | 0.036 | .546 |

*Survey-weighted logistic regression estimates of factors associated with HepA vaccine coverage, NIS-Child 2013*.

## **HepA Vaccine Results 2014**

| Predictor | Odds Ratio | 95% CI | SE | P value |
| --- | --- | --- | --- | --- |
| (Intercept) | 0.17 | (0.06, 0.48) | 0.540 | <.001 |
| Age Group | 2.39 | (2.18, 2.61) | 0.045 | <.001 |
| Household size | 0.96 | (0.90, 1.02) | 0.034 | .205 |
| Breastfeeding History | 0.90 | (0.73, 1.10) | 0.104 | .297 |
| WIC benefits | 0.87 | (0.70, 1.08) | 0.112 | .198 |
| Maternal Education | 1.07 | (0.98, 1.17) | 0.046 | .114 |
| Firstborn | 1.23 | (1.03, 1.47) | 0.091 | .024 |
| Hispanic Ethnicity | 0.77 | (0.63, 0.95) | 0.106 | .014 |
| Income | 1.16 | (1.03, 1.31) | 0.061 | .014 |
| Language | 1.41 | (1.11, 1.79) | 0.122 | .005 |
| Maternal Age | 1.10 | (0.94, 1.30) | 0.084 | .240 |
| Marital Status | 1.07 | (0.88, 1.30) | 0.101 | .519 |
| Racial Identity | 1.03 | (0.93, 1.13) | 0.051 | .610 |
| Sex | 1.07 | (0.93, 1.24) | 0.073 | .361 |
| Insurance Status | 0.85 | (0.77, 0.95) | 0.054 | .003 |
| Region | 0.99 | (0.92, 1.06) | 0.037 | .755 |

*Survey-weighted logistic regression estimates of factors associated with HepA vaccine coverage, NIS-Child 2014*.

## **HepA Vaccine Results 2015**

| Predictor | Odds Ratio | 95% CI | SE | P value |
| --- | --- | --- | --- | --- |
| (Intercept) | 0.20 | (0.07, 0.55) | 0.527 | .002 |
| Age Group | 2.18 | (2.01, 2.37) | 0.043 | <.001 |
| Household size | 0.97 | (0.91, 1.03) | 0.032 | .315 |
| Breastfeeding History | 1.21 | (1.01, 1.45) | 0.093 | .040 |
| WIC benefits | 0.78 | (0.62, 0.98) | 0.116 | .035 |
| Maternal Education | 1.11 | (1.02, 1.21) | 0.045 | .021 |
| Firstborn | 1.33 | (1.13, 1.57) | 0.084 | <.001 |
| Hispanic Ethnicity | 0.87 | (0.71, 1.07) | 0.102 | .183 |
| Income | 1.10 | (0.98, 1.24) | 0.060 | .094 |
| Language | 1.44 | (1.17, 1.77) | 0.105 | <.001 |
| Maternal Age | 1.13 | (0.97, 1.33) | 0.081 | .125 |
| Marital Status | 0.87 | (0.72, 1.05) | 0.097 | .143 |
| Racial Identity | 1.00 | (0.91, 1.10) | 0.047 | .990 |
| Sex | 0.99 | (0.87, 1.13) | 0.069 | .893 |
| Insurance Status | 0.80 | (0.73, 0.88) | 0.047 | <.001 |
| Region | 0.99 | (0.92, 1.06) | 0.035 | .678 |

*Survey-weighted logistic regression estimates of factors associated with HepA vaccine coverage, NIS-Child 2015*.

**HepA Vaccine Results 2016**

| Predictor | Odds Ratio | 95% CI | SE | P value |
| --- | --- | --- | --- | --- |
| (Intercept) | 0.29 | (0.11, 0.78) | 0.510 | .015 |
| Age Group | 2.17 | (1.99, 2.37) | 0.045 | <.001 |
| Household size | 0.94 | (0.89, 1.00) | 0.030 | .042 |
| Breastfeeding History | 0.89 | (0.74, 1.07) | 0.094 | .221 |
| WIC benefits | 0.74 | (0.59, 0.93) | 0.118 | .011 |
| Maternal Education | 1.10 | (1.00, 1.21) | 0.049 | .055 |
| Firstborn | 1.25 | (1.06, 1.48) | 0.084 | .007 |
| Hispanic Ethnicity | 0.84 | (0.69, 1.03) | 0.103 | .093 |
| Income | 1.13 | (1.01, 1.28) | 0.061 | .038 |
| Language | 1.32 | (1.07, 1.61) | 0.104 | .008 |
| Maternal Age | 1.13 | (0.96, 1.33) | 0.085 | .152 |
| Marital Status | 0.95 | (0.78, 1.16) | 0.102 | .613 |
| Racial Identity | 1.05 | (0.96, 1.16) | 0.049 | .295 |
| Sex | 1.08 | (0.94, 1.24) | 0.071 | .282 |
| Insurance Status | 0.83 | (0.73, 0.95) | 0.067 | .006 |
| Region | 1.04 | (0.97, 1.12) | 0.036 | .248 |

*Survey-weighted logistic regression estimates of factors associated with HepA vaccine coverage, NIS-Child 2016*.

## **HepA Vaccine Results 2017**

| Predictor | OR | 95% CI | SE | p |
| --- | --- | --- | --- | --- |
| (Intercept) | 0.24 | (0.09, 0.63) | 0.490 | .004 |
| Age Group | 2.35 | (2.16, 2.56) | 0.043 | <.001 |
| Household size | 0.97 | (0.91, 1.03) | 0.032 | .274 |
| Breastfeeding History | 1.09 | (0.89, 1.33) | 0.104 | .417 |
| WIC benefits | 0.67 | (0.53, 0.86) | 0.124 | .001 |
| Maternal Education | 1.19 | (1.09, 1.31) | 0.047 | <.001 |
| Firstborn | 1.27 | (1.07, 1.50) | 0.086 | .005 |
| Hispanic Ethnicity | 0.73 | (0.60, 0.87) | 0.094 | <.001 |
| Income | 1.24 | (1.10, 1.41) | 0.064 | <.001 |
| Language | 1.22 | (1.02, 1.47) | 0.093 | .031 |
| Maternal Age | 1.06 | (0.91, 1.25) | 0.081 | .454 |
| Marital Status | 0.81 | (0.68, 0.98) | 0.095 | .029 |
| Racial Identity | 1.03 | (0.94, 1.14) | 0.049 | .511 |
| Sex | 0.98 | (0.86, 1.13) | 0.071 | .816 |
| Insurance Status | 0.91 | (0.83, 1.00) | 0.047 | .045 |
| Region | 1.06 | (0.98, 1.14) | 0.038 | .159 |

*Survey-weighted logistic regression estimates of factors associated with HepA vaccine coverage, NIS-Child 2017*.

## **HepA Vaccine Results 2018**

| Predictor | Odds Ratio | 95% CI | SE | P value |
| --- | --- | --- | --- | --- |
| (Intercept) | 0.24 | (0.08, 0.71) | 0.564 | .011 |
| Age Group | 2.50 | (2.27, 2.76) | 0.050 | <.001 |
| Household size | 0.97 | (0.90, 1.06) | 0.042 | .500 |
| Breastfeeding History | 1.15 | (0.92, 1.42) | 0.111 | .222 |
| WIC benefits | 0.94 | (0.73, 1.20) | 0.125 | .603 |
| Maternal Education | 1.09 | (0.98, 1.22) | 0.058 | .125 |
| Firstborn | 1.35 | (1.12, 1.63) | 0.096 | .002 |
| Hispanic Ethnicity | 0.87 | (0.70, 1.10) | 0.116 | .247 |
| Income | 1.01 | (0.89, 1.14) | 0.064 | .917 |
| Language | 1.30 | (1.00, 1.70) | 0.135 | .048 |
| Maternal Age | 1.22 | (1.01, 1.46) | 0.094 | .036 |
| Marital Status | 0.79 | (0.64, 0.99) | 0.112 | .040 |
| Racial Identity | 0.89 | (0.80, 1.00) | 0.059 | .055 |
| Sex | 1.02 | (0.87, 1.19) | 0.080 | .850 |
| Insurance Status | 0.79 | (0.72, 0.87) | 0.051 | <.001 |
| Region | 0.99 | (0.91, 1.07) | 0.044 | .757 |

*Survey-weighted logistic regression estimates of factors associated with HepA vaccine coverage, NIS-Child 2018*.

**HepA Vaccine Results 2019**

| Predictor | Odds Ratio | 95% CI | SE | P value |
| --- | --- | --- | --- | --- |
| (Intercept) | 0.35 | (0.11, 1.10) | 0.581 | .072 |
| Age Group | 2.27 | (2.06, 2.51) | 0.051 | <.001 |
| Household size | 0.89 | (0.83, 0.95) | 0.036 | <.001 |
| Breastfeeding History | 1.33 | (1.07, 1.65) | 0.111 | .010 |
| WIC benefits | 0.72 | (0.57, 0.91) | 0.119 | .005 |
| Maternal Education | 1.14 | (1.02, 1.26) | 0.053 | .017 |
| Firstborn | 1.13 | (0.93, 1.38) | 0.101 | .218 |
| Hispanic Ethnicity | 0.97 | (0.78, 1.21) | 0.112 | .793 |
| Income | 1.20 | (1.06, 1.36) | 0.063 | .004 |
| Language | 1.63 | (1.27, 2.10) | 0.129 | <.001 |
| Maternal Age | 1.06 | (0.89, 1.27) | 0.091 | .521 |
| Marital Status | 0.88 | (0.71, 1.09) | 0.109 | .259 |
| Racial Identity | 0.83 | (0.74, 0.93) | 0.058 | .002 |
| Sex | 0.94 | (0.80, 1.10) | 0.079 | .432 |
| Insurance Status | 0.81 | (0.73, 0.91) | 0.056 | <.001 |
| Region | 1.03 | (0.94, 1.12) | 0.045 | .553 |

*Survey-weighted logistic regression estimates of factors associated with HepA vaccine coverage, NIS-Child 2019*.

**HepA Vaccine Results 2020**

| Predictor | Odds Ratio | 95% CI | SE | P value |
| --- | --- | --- | --- | --- |
| (Intercept) | 0.41 | (0.17, 1.04) | 0.470 | .061 |
| Age Group | 2.48 | (2.30, 2.68) | 0.040 | <.001 |
| Household size | 0.95 | (0.90, 1.01) | 0.028 | .092 |
| Breastfeeding History | 0.94 | (0.79, 1.13) | 0.092 | .538 |
| WIC benefits | 0.85 | (0.70, 1.03) | 0.098 | .093 |
| Maternal Education | 1.10 | (1.01, 1.19) | 0.042 | .026 |
| Firstborn | 1.17 | (1.00, 1.37) | 0.078 | .044 |
| Hispanic Ethnicity | 0.89 | (0.76, 1.06) | 0.085 | .185 |
| Income | 1.12 | (1.01, 1.24) | 0.052 | .035 |
| Language | 1.21 | (0.96, 1.52) | 0.118 | .109 |
| Maternal Age | 1.09 | (0.93, 1.27) | 0.078 | .287 |
| Marital Status | 0.78 | (0.67, 0.92) | 0.081 | .003 |
| Racial Identity | 1.00 | (0.92, 1.08) | 0.039 | .965 |
| Sex | 1.04 | (0.92, 1.17) | 0.062 | .571 |
| Insurance Status | 0.78 | (0.71, 0.87) | 0.052 | <.001 |
| Region | 1.02 | (0.96, 1.08) | 0.031 | .578 |

*Survey-weighted logistic regression estimates of factors associated with HepA vaccine coverage, NIS-Child 2020*.

**HepA R Vaccine Results 2021**

| Predictor | Odds Ratio | 95% CI | SE | P value |
| --- | --- | --- | --- | --- |
| (Intercept) | 1.07 | (0.37, 3.09) | 0.543 | .905 |
| Age Group | 2.28 | (2.08, 2.50) | 0.047 | <.001 |
| Household size | 0.92 | (0.86, 0.98) | 0.033 | .010 |
| Breastfeeding History | 0.91 | (0.73, 1.13) | 0.113 | .382 |
| WIC benefits | 0.63 | (0.51, 0.77) | 0.107 | <.001 |
| Maternal Education | 1.11 | (1.01, 1.23) | 0.048 | .024 |
| Firstborn | 1.12 | (0.94, 1.33) | 0.089 | .211 |
| Hispanic Ethnicity | 0.94 | (0.78, 1.13) | 0.095 | .525 |
| Income | 1.19 | (1.06, 1.34) | 0.059 | .003 |
| Language | 1.19 | (0.90, 1.58) | 0.144 | .219 |
| Maternal Age | 1.14 | (0.96, 1.35) | 0.087 | .129 |
| Marital Status | 0.67 | (0.56, 0.81) | 0.095 | <.001 |
| Racial Identity | 0.93 | (0.85, 1.02) | 0.045 | .113 |
| SEX | 0.96 | (0.83, 1.10) | 0.071 | .540 |
| Insurance Status | 0.84 | (0.74, 0.96) | 0.067 | .010 |
| Region | 1.01 | (0.94, 1.08) | 0.035 | .788 |

*Survey-weighted logistic regression estimates of factors associated with HepA vaccine coverage, NIS-Child 2021*.

## **HepA Vaccine Results 2022**

| Predictor | Odds Ratio | 95% CI | SE | P value |
| --- | --- | --- | --- | --- |
| (Intercept) | 0.22 | (0.07, 0.66) | 0.566 | .007 |
| Age Group | 2.54 | (2.32, 2.77) | 0.045 | <.001 |
| Household size | 0.95 | (0.89, 1.01) | 0.033 | .110 |
| Breastfeeding History | 0.95 | (0.77, 1.17) | 0.106 | .657 |
| WIC benefits | 0.84 | (0.69, 1.02) | 0.100 | .078 |
| Maternal Education | 1.16 | (1.06, 1.27) | 0.047 | .002 |
| Firstborn | 1.28 | (1.08, 1.51) | 0.086 | .004 |
| Hispanic Ethnicity | 0.92 | (0.77, 1.10) | 0.093 | .362 |
| Income | 1.11 | (0.98, 1.25) | 0.062 | .101 |
| Language | 1.25 | (0.96, 1.63) | 0.136 | .104 |
| Maternal Age | 1.40 | (1.18, 1.67) | 0.089 | <.001 |
| Marital Status | 0.81 | (0.68, 0.97) | 0.094 | .026 |
| Racial Identity | 0.92 | (0.84, 1.00) | 0.044 | .048 |
| Sex | 0.93 | (0.81, 1.06) | 0.069 | .292 |
| Insurance Status | 0.81 | (0.72, 0.91) | 0.060 | <.001 |
| Region | 1.06 | (0.99, 1.13) | 0.034 | .082 |

*Survey-weighted logistic regression estimates of factors associated with HepA vaccine coverage, NIS-Child 2022*.

## **HepA Vaccine Results 2023**

| Predictor | Odds Ratio | 95% CI | SE | P value |
| --- | --- | --- | --- | --- |
| (Intercept) | 0.76 | (0.30, 1.95) | 0.481 | .567 |
| Age Group | 2.66 | (2.45, 2.90) | 0.044 | <.001 |
| Household size | 0.90 | (0.84, 0.96) | 0.035 | .003 |
| Breastfeeding History | 0.88 | (0.72, 1.08) | 0.104 | .222 |
| WIC benefits | 0.84 | (0.70, 1.00) | 0.091 | .049 |
| Maternal Education | 1.15 | (1.06, 1.26) | 0.045 | .001 |
| Firstborn | 1.09 | (0.93, 1.29) | 0.082 | .277 |
| Hispanic Ethnicity | 0.82 | (0.69, 0.98) | 0.090 | .028 |
| Income | 1.07 | (0.96, 1.20) | 0.058 | .210 |
| Language | 0.99 | (0.78, 1.26) | 0.123 | .928 |
| Maternal Age | 1.12 | (0.95, 1.32) | 0.084 | .189 |
| Marital Status | 0.86 | (0.73, 1.02) | 0.088 | .087 |
| Racial Identity | 1.00 | (0.92, 1.09) | 0.041 | .974 |
| Sex | 1.01 | (0.89, 1.15) | 0.067 | .884 |
| Insurance Status | 0.75 | (0.69, 0.83) | 0.048 | <.001 |
| Region | 0.99 | (0.92, 1.05) | 0.033 | .676 |

*Survey-weighted logistic regression estimates of factors associated with HepA vaccine coverage, NIS-Child 2023*.

## **HepB Vaccine Results 2010**

| Predictor | Odds Ratio | 95% CI | SE | P value |
| --- | --- | --- | --- | --- |
| (Intercept) | 15.65 | (3.37, 72.60) | 0.783 | <.001 |
| Age Group | 1.21 | (1.07, 1.38) | 0.065 | .003 |
| Household size | 0.87 | (0.81, 0.95) | 0.041 | <.001 |
| Breastfeeding History | 1.19 | (0.93, 1.52) | 0.126 | .177 |
| WIC benefits | 0.54 | (0.39, 0.74) | 0.160 | <.001 |
| Maternal Education | 0.99 | (0.87, 1.13) | 0.068 | .915 |
| Firstborn | 1.07 | (0.86, 1.34) | 0.114 | .559 |
| Hispanic Ethnicity | 0.92 | (0.67, 1.26) | 0.160 | .598 |
| Income | 1.27 | (1.07, 1.51) | 0.089 | .007 |
| Language | 1.64 | (1.09, 2.45) | 0.206 | .017 |
| Maternal Age | 0.99 | (0.79, 1.26) | 0.119 | .962 |
| Marital Status | 1.09 | (0.84, 1.41) | 0.132 | .524 |
| Racial Identity | 0.94 | (0.82, 1.09) | 0.075 | .447 |
| Sex | 1.12 | (0.92, 1.38) | 0.104 | .263 |
| Insurance Status | 0.93 | (0.81, 1.07) | 0.073 | .320 |
| Region | 0.89 | (0.80, 0.99) | 0.056 | .038 |

*Survey-weighted logistic regression estimates of factors associated with HepB vaccine coverage, NIS-Child 200*.

## **HepB Vaccine Results 2011**

| Predictor | Odds Ratio | 95% CI | SE | P value |
| --- | --- | --- | --- | --- |
| (Intercept) | 43.88 | (10.19, 188.87) | 0.745 | <.001 |
| Age Group | 1.08 | (0.97, 1.20) | 0.053 | .162 |
| Household size | 0.92 | (0.85, 0.99) | 0.038 | .026 |
| Breastfeeding History | 1.08 | (0.84, 1.41) | 0.133 | .542 |
| WIC benefits | 0.54 | (0.38, 0.75) | 0.172 | <.001 |
| Maternal Education | 0.96 | (0.84, 1.09) | 0.065 | .501 |
| Firstborn | 1.14 | (0.92, 1.43) | 0.112 | .228 |
| Hispanic Ethnicity | 1.02 | (0.76, 1.36) | 0.148 | .900 |
| Income | 1.10 | (0.90, 1.33) | 0.100 | .352 |
| Language | 1.61 | (1.13, 2.30) | 0.182 | .008 |
| Maternal Age | 0.89 | (0.72, 1.10) | 0.107 | .293 |
| Marital Status | 0.82 | (0.62, 1.07) | 0.139 | .146 |
| Racial Identity | 1.14 | (0.99, 1.31) | 0.073 | .074 |
| Sex | 1.02 | (0.86, 1.22) | 0.090 | .805 |
| Insurance Status | 0.77 | (0.68, 0.88) | 0.066 | <.001 |
| Region | 0.88 | (0.80, 0.97) | 0.052 | .014 |

*Survey-weighted logistic regression estimates of factors associated with HepB vaccine coverage, NIS-Child 2011*.

## **HepB Vaccine Results 2012**

| Predictor | Odds Ratio | 95% CI | SE | P value |
| --- | --- | --- | --- | --- |
| (Intercept) | 22.33 | (6.09, 81.91) | 0.663 | <.001 |
| Age Group | 1.06 | (0.94, 1.19) | 0.059 | .339 |
| Household size | 0.88 | (0.82, 0.95) | 0.038 | .001 |
| Breastfeeding History | 1.28 | (1.00, 1.64) | 0.125 | .049 |
| WIC benefits | 0.57 | (0.42, 0.78) | 0.160 | <.001 |
| Maternal Education | 1.15 | (1.02, 1.29) | 0.061 | .025 |
| Firstborn | 0.99 | (0.79, 1.25) | 0.118 | .951 |
| Hispanic Ethnicity | 0.90 | (0.67, 1.22) | 0.155 | .502 |
| Income | 1.01 | (0.87, 1.19) | 0.081 | .862 |
| Language | 1.08 | (0.77, 1.50) | 0.169 | .653 |
| Maternal Age | 1.04 | (0.83, 1.29) | 0.111 | .753 |
| Marital Status | 0.79 | (0.63, 0.99) | 0.114 | .041 |
| Racial Identity | 1.16 | (1.03, 1.31) | 0.061 | .017 |
| Sex | 1.06 | (0.88, 1.28) | 0.096 | .549 |
| Insurance Status | 0.81 | (0.72, 0.91) | 0.059 | <.001 |
| Region | 1.01 | (0.91, 1.11) | 0.050 | .908 |

*Survey-weighted logistic regression estimates of factors associated with HepB vaccine coverage, NIS-Child 2012*.

## **HepB Vaccine Results 2013**

| Predictor | Odds Ratio | 95% CI | SE | P value |
| --- | --- | --- | --- | --- |
| (Intercept) | 17.79 | (3.36, 94.33) | 0.851 | <.001 |
| Age Group | 1.14 | (0.99, 1.31) | 0.070 | .067 |
| Household size | 0.96 | (0.86, 1.08) | 0.057 | .503 |
| Breastfeeding History | 1.08 | (0.80, 1.45) | 0.153 | .628 |
| WIC benefits | 0.61 | (0.45, 0.83) | 0.155 | .002 |
| Maternal Education | 1.10 | (0.96, 1.27) | 0.070 | .158 |
| Firstborn | 1.30 | (0.97, 1.72) | 0.145 | .074 |
| Hispanic Ethnicity | 0.91 | (0.66, 1.26) | 0.165 | .567 |
| Income | 1.10 | (0.90, 1.33) | 0.099 | .345 |
| Language | 1.10 | (0.72, 1.69) | 0.218 | .662 |
| Maternal Age | 1.09 | (0.80, 1.49) | 0.158 | .578 |
| Marital Status | 0.88 | (0.64, 1.19) | 0.157 | .402 |
| Racial Identity | 1.15 | (0.96, 1.37) | 0.089 | .119 |
| Sex | 0.90 | (0.71, 1.14) | 0.120 | .401 |
| Insurance Status | 0.71 | (0.61, 0.83) | 0.079 | <.001 |
| Region | 0.91 | (0.81, 1.02) | 0.060 | .113 |

*Survey-weighted logistic regression estimates of factors associated with HepB vaccine coverage, NIS-Child 2013*.

## **HepB Vaccine Results 2014**

| Predictor | Odds Ratio | 95% CI | SE | P value |
| --- | --- | --- | --- | --- |
| (Intercept) | 19.37 | (2.20, 170.79) | 1.111 | .008 |
| Age Group | 1.06 | (0.91, 1.23) | 0.077 | .446 |
| Household size | 0.97 | (0.87, 1.07) | 0.051 | .507 |
| Breastfeeding History | 1.08 | (0.79, 1.50) | 0.164 | .623 |
| WIC benefits | 0.76 | (0.54, 1.09) | 0.180 | .133 |
| Maternal Education | 1.06 | (0.91, 1.24) | 0.078 | .449 |
| Firstborn | 0.92 | (0.69, 1.24) | 0.150 | .599 |
| Hispanic Ethnicity | 0.84 | (0.55, 1.29) | 0.217 | .421 |
| Income | 1.18 | (0.95, 1.46) | 0.109 | .131 |
| Language | 1.25 | (0.74, 2.11) | 0.265 | .398 |
| Maternal Age | 0.89 | (0.65, 1.23) | 0.161 | .487 |
| Marital Status | 1.31 | (0.93, 1.82) | 0.171 | .119 |
| Racial Identity | 1.18 | (1.01, 1.38) | 0.082 | .042 |
| Sex | 1.02 | (0.81, 1.28) | 0.119 | .887 |
| Insurance Status | 0.76 | (0.64, 0.92) | 0.092 | .004 |
| Region | 0.88 | (0.79, 0.99) | 0.059 | .033 |

*Survey-weighted logistic regression estimates of factors associated with HepB vaccine coverage, NIS-Child 2014*.

## **HepB Vaccine Results 2015**

| Predictor | Odds Ratio | 95% CI | SE | P value |
| --- | --- | --- | --- | --- |
| (Intercept) | 34.47 | (4.13, 287.35) | 1.082 | .001 |
| Age Group | 1.04 | (0.89, 1.20) | 0.075 | .642 |
| Household size | 0.90 | (0.82, 0.99) | 0.047 | .025 |
| Breastfeeding History | 1.14 | (0.84, 1.55) | 0.155 | .387 |
| WIC benefits | 0.61 | (0.44, 0.86) | 0.173 | .004 |
| Maternal Education | 1.13 | (0.96, 1.32) | 0.080 | .136 |
| Firstborn | 1.06 | (0.80, 1.41) | 0.145 | .696 |
| Hispanic Ethnicity | 0.90 | (0.64, 1.25) | 0.169 | .517 |
| Income | 1.01 | (0.84, 1.21) | 0.093 | .956 |
| Language | 1.36 | (0.90, 2.06) | 0.211 | .144 |
| Maternal Age | 1.10 | (0.84, 1.45) | 0.140 | .492 |
| Marital Status | 0.73 | (0.50, 1.08) | 0.196 | .115 |
| Racial Identity | 1.13 | (0.96, 1.33) | 0.082 | .141 |
| Sex | 0.94 | (0.75, 1.19) | 0.118 | .602 |
| Insurance Status | 0.82 | (0.71, 0.95) | 0.074 | .007 |
| Region | 0.95 | (0.84, 1.07) | 0.063 | .418 |

*Survey-weighted logistic regression estimates of factors associated with HepB vaccine coverage, NIS-Child 2015*.

## **HepB Vaccine Results 2016**

| Predictor | Odds Ratio | 95% CI | SE | P value |
| --- | --- | --- | --- | --- |
| (Intercept) | 10.17 | (1.44, 72.05) | 0.999 | .020 |
| Age Group | 1.06 | (0.91, 1.24) | 0.079 | .424 |
| Household size | 0.98 | (0.89, 1.08) | 0.050 | .713 |
| Breastfeeding History | 1.19 | (0.83, 1.68) | 0.179 | .342 |
| WIC benefits | 0.64 | (0.40, 1.01) | 0.237 | .055 |
| Maternal Education | 1.20 | (0.99, 1.46) | 0.099 | .064 |
| Firstborn | 0.88 | (0.67, 1.16) | 0.138 | .373 |
| Hispanic Ethnicity | 1.22 | (0.86, 1.74) | 0.181 | .269 |
| Income | 1.13 | (0.87, 1.47) | 0.134 | .354 |
| Language | 1.35 | (0.86, 2.11) | 0.230 | .194 |
| Maternal Age | 0.92 | (0.67, 1.25) | 0.158 | .596 |
| Marital Status | 1.50 | (1.00, 2.25) | 0.206 | .048 |
| Racial Identity | 0.94 | (0.78, 1.14) | 0.098 | .521 |
| Sex | 0.96 | (0.74, 1.25) | 0.136 | .768 |
| Insurance Status | 0.76 | (0.62, 0.94) | 0.105 | .010 |
| Region | 0.77 | (0.68, 0.88) | 0.066 | <.001 |

*Survey-weighted logistic regression estimates of factors associated with HepB vaccine coverage, NIS-Child 2016.*

## **HepB Vaccine Results 2017**

| Predictor | Odds Ratio | 95% CI | SE | P value |
| --- | --- | --- | --- | --- |
| (Intercept) | 11.11 | (2.20, 56.08) | 0.826 | .004 |
| Age Group | 1.05 | (0.90, 1.22) | 0.075 | .526 |
| Household size | 0.90 | (0.82, 1.00) | 0.050 | .044 |
| Breastfeeding History | 1.03 | (0.73, 1.46) | 0.177 | .866 |
| WIC benefits | 0.49 | (0.33, 0.74) | 0.207 | <.001 |
| Maternal Education | 1.17 | (1.01, 1.36) | 0.076 | .039 |
| Firstborn | 1.02 | (0.77, 1.36) | 0.146 | .869 |
| Hispanic Ethnicity | 1.00 | (0.73, 1.37) | 0.162 | .997 |
| Income | 1.36 | (1.11, 1.67) | 0.103 | .003 |
| Language | 1.24 | (0.88, 1.75) | 0.174 | .213 |
| Maternal Age | 1.36 | (1.06, 1.74) | 0.127 | .016 |
| Marital Status | 1.17 | (0.88, 1.56) | 0.146 | .289 |
| Racial Identity | 0.98 | (0.82, 1.16) | 0.088 | .784 |
| Sex | 1.01 | (0.79, 1.28) | 0.122 | .947 |
| Insurance Status | 0.85 | (0.72, 0.99) | 0.083 | .042 |
| Region | 0.89 | (0.79, 1.01) | 0.064 | .075 |

*Survey-weighted logistic regression estimates of factors associated with HepB vaccine coverage, NIS-Child 2017*.

## **HepB Vaccine Results 2018**

| Predictor | Odds Ratio | 95% CI | SE | P value |
| --- | --- | --- | --- | --- |
| (Intercept) | 17.87 | (2.93, 108.97) | 0.922 | .002 |
| Age Group | 1.28 | (1.10, 1.49) | 0.077 | .001 |
| Household size | 0.90 | (0.79, 1.01) | 0.062 | .078 |
| Breastfeeding History | 1.32 | (0.94, 1.84) | 0.171 | .106 |
| WIC benefits | 0.84 | (0.55, 1.30) | 0.222 | .445 |
| Maternal Education | 1.04 | (0.89, 1.22) | 0.081 | .626 |
| Firstborn | 1.27 | (0.96, 1.67) | 0.142 | .094 |
| Hispanic Ethnicity | 0.97 | (0.68, 1.40) | 0.185 | .884 |
| Income | 1.05 | (0.79, 1.38) | 0.142 | .736 |
| Language | 1.59 | (1.09, 2.31) | 0.191 | .015 |
| Maternal Age | 0.88 | (0.65, 1.19) | 0.157 | .408 |
| Marital Status | 0.77 | (0.53, 1.11) | 0.189 | .156 |
| Racial Identity | 0.95 | (0.82, 1.11) | 0.076 | .523 |
| Sex | 1.12 | (0.87, 1.43) | 0.126 | .376 |
| Insurance Status | 0.69 | (0.61, 0.79) | 0.067 | <.001 |
| Region | 0.91 | (0.79, 1.04) | 0.071 | .159 |

*Survey-weighted logistic regression estimates of factors associated with HepB vaccine coverage, NIS-Child 2018*.

## **HepB Vaccine Results 2019**

| Predictor | Odds Ratio | 95% CI | SE | P value |
| --- | --- | --- | --- | --- |
| (Intercept) | 14.83 | (2.36, 93.04) | 0.937 | .004 |
| Age Group | 1.01 | (0.87, 1.18) | 0.078 | .872 |
| Household size | 0.95 | (0.83, 1.09) | 0.068 | .465 |
| Breastfeeding History | 1.58 | (1.10, 2.26) | 0.184 | .013 |
| WIC benefits | 0.49 | (0.33, 0.73) | 0.203 | <.001 |
| Maternal Education | 1.14 | (0.93, 1.39) | 0.103 | .213 |
| Firstborn | 1.10 | (0.82, 1.48) | 0.151 | .529 |
| Hispanic Ethnicity | 0.91 | (0.62, 1.32) | 0.191 | .609 |
| Income | 1.35 | (1.10, 1.67) | 0.107 | .005 |
| Language | 1.43 | (0.89, 2.31) | 0.243 | .139 |
| Maternal Age | 1.16 | (0.88, 1.52) | 0.140 | .300 |
| Marital Status | 0.78 | (0.51, 1.20) | 0.218 | .263 |
| Racial Identity | 0.82 | (0.68, 0.98) | 0.096 | .034 |
| Sex | 1.09 | (0.83, 1.43) | 0.140 | .545 |
| Insurance Status | 0.84 | (0.71, 1.00) | 0.088 | .056 |
| Region | 0.92 | (0.79, 1.08) | 0.081 | .311 |

*Survey-weighted logistic regression estimates of factors associated with HepB vaccine coverage, NIS-Child 2019*.

## **HepB Vaccine Results 2020**

| Predictor | Odds Ratio | 95% CI | SE | P value |
| --- | --- | --- | --- | --- |
| (Intercept) | 6.54 | (0.91, 47.15) | 1.008 | .062 |
| Age Group | 1.21 | (1.05, 1.41) | 0.076 | .010 |
| Household size | 0.89 | (0.80, 0.98) | 0.051 | .018 |
| Breastfeeding History | 1.65 | (1.21, 2.27) | 0.161 | .002 |
| WIC benefits | 0.64 | (0.43, 0.95) | 0.203 | .026 |
| Maternal Education | 1.08 | (0.93, 1.25) | 0.075 | .291 |
| Firstborn | 1.08 | (0.80, 1.47) | 0.155 | .601 |
| Hispanic Ethnicity | 1.01 | (0.74, 1.40) | 0.164 | .930 |
| Income | 1.37 | (1.13, 1.66) | 0.098 | .001 |
| Language | 0.93 | (0.64, 1.35) | 0.191 | .703 |
| Maternal Age | 1.16 | (0.89, 1.51) | 0.134 | .273 |
| Marital Status | 1.11 | (0.83, 1.48) | 0.147 | .473 |
| Racial Identity | 0.94 | (0.82, 1.07) | 0.068 | .339 |
| Sex | 1.09 | (0.87, 1.38) | 0.118 | .452 |
| Insurance Status | 0.87 | (0.74, 1.03) | 0.083 | .098 |
| Region | 0.99 | (0.88, 1.12) | 0.061 | .906 |

*Survey-weighted logistic regression estimates of factors associated with HepB vaccine coverage, NIS-Child 2020*.

## **HepB Vaccine Results 2021**

| Predictor | Odds Ratio | 95% CI | SE | P value |
| --- | --- | --- | --- | --- |
| (Intercept) | 22.65 | (4.25, 120.72) | 0.854 | <.001 |
| Age Group | 1.03 | (0.89, 1.21) | 0.078 | .670 |
| Household size | 0.97 | (0.88, 1.07) | 0.051 | .548 |
| Breastfeeding History | 1.05 | (0.73, 1.52) | 0.186 | .778 |
| WIC benefits | 0.70 | (0.51, 0.95) | 0.155 | .021 |
| Maternal Education | 1.11 | (0.95, 1.29) | 0.079 | .207 |
| Firstborn | 1.18 | (0.88, 1.57) | 0.148 | .272 |
| Hispanic Ethnicity | 0.86 | (0.63, 1.18) | 0.161 | .362 |
| Income | 1.26 | (1.05, 1.51) | 0.093 | .014 |
| Language | 0.95 | (0.56, 1.61) | 0.270 | .835 |
| Maternal Age | 1.25 | (0.96, 1.63) | 0.136 | .100 |
| Marital Status | 1.01 | (0.75, 1.35) | 0.149 | .947 |
| Racial Identity | 0.96 | (0.83, 1.11) | 0.076 | .577 |
| Sex | 0.94 | (0.74, 1.20) | 0.125 | .621 |
| Insurance Status | 0.73 | (0.58, 0.91) | 0.114 | .005 |
| Region | 0.92 | (0.80, 1.04) | 0.066 | .176 |

*Survey-weighted logistic regression estimates of factors associated with HepB vaccine coverage, NIS-Child 2021*.

## **HepB Vaccine Results 2022**

| Predictor | Odds Ratio | 95% CI | SE | P value |
| --- | --- | --- | --- | --- |
| (Intercept) | 80.03 | (10.14, 631.53) | 1.054 | <.001 |
| Age Group | 1.13 | (1.00, 1.28) | 0.065 | .056 |
| Household size | 0.84 | (0.76, 0.93) | 0.054 | .001 |
| Breastfeeding History | 1.10 | (0.73, 1.64) | 0.205 | .650 |
| WIC benefits | 0.69 | (0.49, 0.99) | 0.182 | .045 |
| Maternal Education | 1.16 | (1.00, 1.35) | 0.076 | .050 |
| Firstborn | 0.88 | (0.65, 1.20) | 0.156 | .423 |
| Hispanic Ethnicity | 0.79 | (0.58, 1.07) | 0.159 | .130 |
| Income | 1.11 | (0.86, 1.43) | 0.131 | .440 |
| Language | 0.87 | (0.61, 1.25) | 0.183 | .464 |
| Maternal Age | 0.97 | (0.72, 1.32) | 0.155 | .868 |
| Marital Status | 0.93 | (0.65, 1.34) | 0.182 | .710 |
| Racial Identity | 0.91 | (0.79, 1.06) | 0.073 | .222 |
| Sex | 1.00 | (0.79, 1.27) | 0.122 | .988 |
| Insurance Status | 0.78 | (0.65, 0.93) | 0.089 | .005 |
| Region | 1.00 | (0.89, 1.13) | 0.060 | .968 |

*Survey-weighted logistic regression estimates of factors associated with HepB vaccine coverage, NIS-Child 2022*.

## **HepB Vaccine Results 2023**

| Predictor | Odds Ratio | 95% CI | SE | P value |
| --- | --- | --- | --- | --- |
| (Intercept) | 122.54 | (27.45, 546.95) | 0.763 | <.001 |
| Age Group | 0.97 | (0.8, 1.12) | 0.070- | .720 |
| Household size | 0.88 | (0.79, 0.99) | 0.0561 | .023 |
| Breastfeeding History | 0.75 | (0.55, 1.01) | 0.155 | .059 |
| WIC benefits | 0.63 | (0.48, 0.83) | 0.139 | <.001 |
| Maternal Education | 1.18 | (1.03, 1.34) | 0.066 | .014 |
| Firstborn | 1.04 | (0.80, 1.36) | 0.133 | .740 |
| Hispanic Ethnicity | 0.71 | (0.54, 0.94) | 0.142 | .017 |
| Income | 1.27 | (1.05, 1.52) | 0.094 | .011 |
| Language | 0.91 | (0.65, 1.28) | 0.173 | .582 |
| Maternal Age | 1.23 | (0.96, 1.58) | 0.128 | .102 |
| Marital Status | 0.88 | (0.68, 1.13) | 0.128 | .303 |
| Racial Identity | 1.03 | (0.90, 1.18) | 0.070 | .701 |
| Sex | 0.95 | (0.75, 1.18) | 0.112 | .621 |
| Insurance Status | 0.68 | (0.58, 0.80) | 0.0770 | <.001 |
| Region | 0.83 | (0.74, 0.92) | 0.056 | <.001 |

*Survey-weighted logistic regression estimates of factors associated with HepB vaccine coverage, NIS-Child 2023*

## **Hib Vaccine Results 2010**

| Predictor | Odds Ratio | 95% CI | SE | P value |
| --- | --- | --- | --- | --- |
| (Intercept) | 174.16 | (30.48, 995.03) | 0.889 | <.001 |
| Age Group | 0.76 | (0.67, 0.86) | 0.066 | <.001 |
| Household size | 0.87 | (0.81, 0.94) | 0.040 | <.001 |
| Breastfeeding History | 1.05 | (0.83, 1.32) | 0.119 | .706 |
| WIC benefits | 0.57 | (0.41, 0.80) | 0.173 | .001 |
| Maternal Education | 1.10 | (0.97, 1.24) | 0.062 | .127 |
| Firstborn | 0.96 | (0.77, 1.20) | 0.113 | .738 |
| Hispanic Ethnicity | 0.57 | (0.41, 0.79) | 0.167 | <.001 |
| Income | 1.26 | (1.09, 1.45) | 0.074 | .002 |
| Language | 1.11 | (0.77, 1.58) | 0.182 | .580 |
| Maternal Age | 1.13 | (0.91, 1.40) | 0.111 | .279 |
| Marital Status | 0.99 | (0.76, 1.29) | 0.135 | .949 |
| Racial Identity | 0.93 | (0.81, 1.08) | 0.073 | .341 |
| Sex | 1.05 | (0.86, 1.29) | 0.102 | .618 |
| Insurance Status | 0.74 | (0.63, 0.87) | 0.080 | <.001 |
| Region | 0.88 | (0.78, 0.99) | 0.059 | .031 |

*Survey-weighted logistic regression estimates of factors associated with Hib vaccine coverage, NIS-Child 2010*.

## **Hib Vaccine Results 2011**

| Predictor | Odds Ratio | 95% CI | SE | P value |
| --- | --- | --- | --- | --- |
| (Intercept) | 17.94 | (3.38, 95.20) | 0.851 | <.001 |
| Age Group | 0.94 | (0.82, 1.07) | 0.067 | .328 |
| Household size | 0.89 | (0.82, 0.98) | 0.046 | .013 |
| Breastfeeding History | 1.11 | (0.83, 1.49) | 0.149 | .479 |
| WIC benefits | 0.64 | (0.43, 0.96) | 0.203 | .029 |
| Maternal Education | 1.03 | (0.88, 1.21) | 0.081 | .692 |
| Firstborn | 1.42 | (1.08, 1.86) | 0.137 | .011 |
| Hispanic Ethnicity | 0.82 | (0.58, 1.15) | 0.174 | .257 |
| Income | 1.33 | (1.08, 1.63) | 0.106 | .008 |
| Language | 1.92 | (1.27, 2.90) | 0.210 | .002 |
| Maternal Age | 1.15 | (0.92, 1.43) | 0.112 | .211 |
| Marital Status | 1.01 | (0.74, 1.37) | 0.155 | .954 |
| Racial Identity | 0.96 | (0.82, 1.12) | 0.080 | .610 |
| Sex | 1.16 | (0.93, 1.44) | 0.110 | .182 |
| Insurance Status | 0.67 | (0.59, 0.76) | 0.064 | <.001 |
| Region | 0.98 | (0.87, 1.11) | 0.061 | .787 |

*Survey-weighted logistic regression estimates of factors associated with Hib vaccine coverage, NIS-Child 2011*.

## **Hib Vaccine Results 2012**

| Predictor | Odds Ratio | 95% CI | SE | P value |
| --- | --- | --- | --- | --- |
| (Intercept) | 53.98 | (12.56, 232.08) | 0.744 | <.001 |
| Age Group | 1.02 | (0.88, 1.18) | 0.076 | .827 |
| Household size | 0.86 | (0.79, 0.93) | 0.044 | <.001 |
| Breastfeeding History | 1.08 | (0.81, 1.43) | 0.146 | .617 |
| WIC benefits | 0.64 | (0.45, 0.91) | 0.181 | .014 |
| Maternal Education | 1.20 | (1.05, 1.37) | 0.069 | .008 |
| Firstborn | 0.96 | (0.72, 1.28) | 0.147 | .784 |
| Hispanic Ethnicity | 0.79 | (0.54, 1.14) | 0.189 | .205 |
| Income | 1.12 | (0.92, 1.37) | 0.103 | .255 |
| Language | 1.37 | (0.90, 2.08) | 0.214 | .142 |
| Maternal Age | 1.00 | (0.76, 1.32) | 0.143 | .993 |
| Marital Status | 0.99 | (0.76, 1.29) | 0.135 | .931 |
| Racial Identity | 0.98 | (0.85, 1.12) | 0.071 | .734 |
| Sex | 1.04 | (0.82, 1.31) | 0.120 | .752 |
| Insurance Status | 0.67 | (0.59, 0.76) | 0.066 | <.001 |
| Region | 1.00 | (0.88, 1.13) | 0.064 | .983 |

*Survey-weighted logistic regression estimates of factors associated with Hib vaccine coverage, NIS-Child 2012*.

## **Hib Vaccine Results 2013**

| Predictor | Odds Ratio | 95% CI | SE | P value |
| --- | --- | --- | --- | --- |
| (Intercept) | 38.47 | (5.79, 255.68) | 0.966 | <.001 |
| Age Group | 1.21 | (1.01, 1.44) | 0.089 | .035 |
| Household size | 0.95 | (0.84, 1.08) | 0.063 | .417 |
| Breastfeeding History | 1.27 | (0.90, 1.80) | 0.178 | .179 |
| WIC benefits | 0.64 | (0.44, 0.92) | 0.192 | .018 |
| Maternal Education | 1.22 | (1.04, 1.44) | 0.084 | .016 |
| Firstborn | 1.47 | (1.03, 2.09) | 0.181 | .034 |
| Hispanic Ethnicity | 0.57 | (0.38, 0.86) | 0.205 | .007 |
| Income | 1.15 | (0.92, 1.44) | 0.115 | .235 |
| Language | 0.97 | (0.62, 1.52) | 0.228 | .907 |
| Maternal Age | 1.41 | (0.99, 2.00) | 0.180 | .059 |
| Marital Status | 0.69 | (0.50, 0.95) | 0.166 | .025 |
| Racial Identity | 0.95 | (0.78, 1.16) | 0.100 | .637 |
| Sex | 1.02 | (0.76, 1.36) | 0.147 | .911 |
| Insurance Status | 0.63 | (0.53, 0.75) | 0.090 | <.001 |
| Region | 0.83 | (0.71, 0.97) | 0.080 | .021 |

*Survey-weighted logistic regression estimates of factors associated with Hib vaccine coverage, NIS-Child 2013*.

## **Hib Vaccine Results 2014**

| Predictor | Odds Ratio | 95% CI | SE | P value |
| --- | --- | --- | --- | --- |
| (Intercept) | 17.69 | (1.82, 171.70) | 1.160 | .013 |
| Age Group | 1.28 | (1.08, 1.51) | 0.084 | .004 |
| Household size | 0.90 | (0.82, 0.99) | 0.050 | .037 |
| Breastfeeding History | 0.91 | (0.66, 1.24) | 0.160 | .535 |
| WIC benefits | 0.86 | (0.59, 1.25) | 0.191 | .421 |
| Maternal Education | 1.21 | (1.03, 1.42) | 0.084 | .024 |
| Firstborn | 1.30 | (0.93, 1.82) | 0.170 | .119 |
| Hispanic Ethnicity | 0.54 | (0.34, 0.87) | 0.236 | .010 |
| Income | 1.35 | (1.07, 1.71) | 0.120 | .012 |
| Language | 1.10 | (0.68, 1.78) | 0.244 | .695 |
| Maternal Age | 1.11 | (0.78, 1.57) | 0.178 | .567 |
| Marital Status | 1.24 | (0.88, 1.75) | 0.175 | .220 |
| Racial Identity | 1.07 | (0.92, 1.26) | 0.080 | .375 |
| Sex | 0.97 | (0.75, 1.25) | 0.130 | .802 |
| Insurance Status | 0.76 | (0.63, 0.91) | 0.095 | .004 |
| Region | 0.86 | (0.76, 0.98) | 0.065 | .023 |

*Survey-weighted logistic regression estimates of factors associated with Hib vaccine coverage, NIS-Child 2014*.

## **Hib Vaccine Results 2015**

| Predictor | Odds Ratio | 95% CI | SE | P value |
| --- | --- | --- | --- | --- |
| (Intercept) | 13.28 | (2.71, 65.06) | 0.811 | .001 |
| Age Group | 1.08 | (0.94, 1.25) | 0.071 | .266 |
| Household size | 0.89 | (0.81, 0.98) | 0.047 | .014 |
| Breastfeeding History | 0.99 | (0.75, 1.31) | 0.142 | .958 |
| WIC benefits | 0.64 | (0.45, 0.91) | 0.180 | .013 |
| Maternal Education | 1.26 | (1.09, 1.45) | 0.074 | .002 |
| Firstborn | 1.39 | (1.05, 1.84) | 0.143 | .021 |
| Hispanic Ethnicity | 0.67 | (0.49, 0.91) | 0.159 | .011 |
| Income | 1.28 | (1.04, 1.57) | 0.103 | .017 |
| Language | 1.23 | (0.89, 1.70) | 0.163 | .200 |
| Maternal Age | 1.30 | (1.02, 1.67) | 0.126 | .037 |
| Marital Status | 0.92 | (0.69, 1.23) | 0.149 | .588 |
| Racial Identity | 0.94 | (0.81, 1.09) | 0.077 | .400 |
| Sex | 1.14 | (0.91, 1.42) | 0.113 | .244 |
| Insurance Status | 0.74 | (0.64, 0.84) | 0.071 | <.001 |
| Region | 1.01 | (0.90, 1.13) | 0.058 | .885 |

*Survey-weighted logistic regression estimates of factors associated with Hib vaccine coverage, NIS-Child 2015*.

## **Hib Vaccine Results 2016**

| Predictor | Odds Ratio | 95% CI | SE | P value |
| --- | --- | --- | --- | --- |
| (Intercept) | 31.97 | (6.45, 158.43) | 0.817 | <.001 |
| Age Group | 1.11 | (0.94, 1.32) | 0.085 | .203 |
| Household size | 0.91 | (0.82, 1.01) | 0.052 | .077 |
| Breastfeeding History | 0.84 | (0.61, 1.17) | 0.166 | .311 |
| WIC benefits | 0.47 | (0.33, 0.68) | 0.184 | <.001 |
| Maternal Education | 1.21 | (1.02, 1.44) | 0.087 | .028 |
| Firstborn | 0.95 | (0.70, 1.29) | 0.154 | .754 |
| Hispanic Ethnicity | 0.89 | (0.61, 1.29) | 0.190 | .540 |
| Income | 1.52 | (1.23, 1.87) | 0.106 | <.001 |
| Language | 1.51 | (0.99, 2.28) | 0.212 | .054 |
| Maternal Age | 1.06 | (0.79, 1.42) | 0.150 | .716 |
| Marital Status | 1.35 | (0.97, 1.88) | 0.168 | .072 |
| Racial Identity | 1.02 | (0.85, 1.21) | 0.090 | .851 |
| Sex | 0.86 | (0.67, 1.10) | 0.128 | .232 |
| Insurance Status | 0.64 | (0.54, 0.75) | 0.082 | <.001 |
| Region | 0.83 | (0.73, 0.94) | 0.064 | .003 |

*Survey-weighted logistic regression estimates of factors associated with Hib vaccine coverage, NIS-Child 2016*.

## **Hib Vaccine Results 2017**

| Predictor | Odds Ratio | 95% CI | SE | P value |
| --- | --- | --- | --- | --- |
| (Intercept) | 20.10 | (2.66, 152.13) | 1.033 | .004 |
| Age Group | 1.08 | (0.94, 1.24) | 0.072 | .294 |
| Household size | 0.89 | (0.80, 0.99) | 0.055 | .038 |
| Breastfeeding History | 0.87 | (0.58, 1.29) | 0.201 | .477 |
| WIC benefits | 0.71 | (0.53, 0.95) | 0.149 | .021 |
| Maternal Education | 1.18 | (1.03, 1.36) | 0.070 | .017 |
| Firstborn | 1.06 | (0.79, 1.42) | 0.149 | .712 |
| Hispanic Ethnicity | 0.80 | (0.55, 1.15) | 0.185 | .221 |
| Income | 1.34 | (1.14, 1.59) | 0.086 | <.001 |
| Language | 1.17 | (0.84, 1.61) | 0.165 | .349 |
| Maternal Age | 1.32 | (1.02, 1.72) | 0.132 | .034 |
| Marital Status | 1.10 | (0.78, 1.54) | 0.173 | .588 |
| Racial Identity | 0.91 | (0.77, 1.09) | 0.091 | .318 |
| Sex | 0.94 | (0.74, 1.21) | 0.126 | .646 |
| Insurance Status | 0.73 | (0.64, 0.84) | 0.071 | <.001 |
| Region | 0.95 | (0.83, 1.08) | 0.067 | .405 |

*Survey-weighted logistic regression estimates of factors associated with Hib vaccine coverage, NIS-Child 2017*.

## **Hib Vaccine Results 2018**

| Predictor | Odds Ratio | 95% CI | SE | P value |
| --- | --- | --- | --- | --- |
| (Intercept) | 17.28 | (2.42, 123.36) | 1.003 | .005 |
| Age Group | 1.20 | (1.05, 1.39) | 0.072 | .009 |
| Household size | 0.87 | (0.78, 0.98) | 0.060 | .024 |
| Breastfeeding History | 1.14 | (0.83, 1.57) | 0.163 | .418 |
| WIC benefits | 0.84 | (0.52, 1.34) | 0.243 | .459 |
| Maternal Education | 1.17 | (0.95, 1.43) | 0.104 | .132 |
| Firstborn | 1.15 | (0.82, 1.62) | 0.173 | .413 |
| Hispanic Ethnicity | 0.83 | (0.56, 1.23) | 0.203 | .348 |
| Income | 1.13 | (0.89, 1.43) | 0.120 | .304 |
| Language | 1.67 | (1.07, 2.60) | 0.226 | .023 |
| Maternal Age | 1.14 | (0.82, 1.57) | 0.166 | .442 |
| Marital Status | 0.86 | (0.62, 1.18) | 0.165 | .343 |
| Racial Identity | 0.93 | (0.79, 1.09) | 0.082 | .382 |
| Sex | 1.10 | (0.85, 1.44) | 0.134 | .461 |
| Insurance Status | 0.65 | (0.55, 0.76) | 0.082 | <.001 |
| Region | 0.87 | (0.74, 1.02) | 0.079 | .077 |

*Survey-weighted logistic regression estimates of factors associated with Hib vaccine coverage, NIS-Child 2018*.

## **Hib Vaccine Results 2019**

| Predictor | Odds Ratio | 95% CI | SE | P value |
| --- | --- | --- | --- | --- |
| (Intercept) | 4.63 | (0.82, 26.23) | 0.885 | .083 |
| Age Group | 1.08 | (0.92, 1.27) | 0.082 | .356 |
| Household size | 0.97 | (0.87, 1.09) | 0.058 | .614 |
| Breastfeeding History | 1.28 | (0.93, 1.76) | 0.162 | .126 |
| WIC benefits | 0.66 | (0.45, 0.98) | 0.200 | .041 |
| Maternal Education | 1.42 | (1.16, 1.73) | 0.101 | <.001 |
| Firstborn | 1.59 | (1.19, 2.13) | 0.149 | .002 |
| Hispanic Ethnicity | 0.78 | (0.54, 1.13) | 0.190 | .195 |
| Income | 1.16 | (0.96, 1.41) | 0.098 | .119 |
| Language | 1.44 | (0.99, 2.09) | 0.190 | .054 |
| Maternal Age | 1.40 | (1.07, 1.82) | 0.135 | .013 |
| Marital Status | 0.79 | (0.53, 1.18) | 0.201 | .251 |
| Racial Identity | 0.80 | (0.67, 0.95) | 0.089 | .011 |
| Sex | 0.87 | (0.68, 1.13) | 0.131 | .296 |
| Insurance Status | 0.86 | (0.71, 1.05) | 0.100 | .129 |
| Region | 0.96 | (0.83, 1.12) | 0.076 | .599 |

*Survey-weighted logistic regression estimates of factors associated with Hib vaccine coverage, NIS-Child 2019*.

## **Hib Vaccine Results 2020**

| Predictor | Odds Ratio | 95% CI | SE | P value |
| --- | --- | --- | --- | --- |
| (Intercept) | 2.28 | (0.37, 13.85) | 0.921 | .371 |
| Age Group | 1.24 | (1.08, 1.42) | 0.071 | .003 |
| Household size | 0.98 | (0.89, 1.07) | 0.047 | .663 |
| Breastfeeding History | 1.16 | (0.88, 1.54) | 0.144 | .293 |
| WIC benefits | 0.69 | (0.50, 0.95) | 0.164 | .022 |
| Maternal Education | 1.22 | (1.06, 1.40) | 0.071 | .005 |
| Firstborn | 1.39 | (1.04, 1.87) | 0.150 | .028 |
| Hispanic Ethnicity | 0.91 | (0.67, 1.24) | 0.156 | .560 |
| Income | 1.52 | (1.27, 1.81) | 0.090 | <.001 |
| Language | 1.50 | (1.02, 2.21) | 0.197 | .039 |
| Maternal Age | 1.40 | (1.09, 1.79) | 0.126 | .008 |
| Marital Status | 0.97 | (0.74, 1.26) | 0.136 | .802 |
| Racial Identity | 0.84 | (0.73, 0.96) | 0.069 | .009 |
| Sex | 1.13 | (0.91, 1.41) | 0.111 | .254 |
| Insurance Status | 0.68 | (0.59, 0.78) | 0.072 | <.001 |
| Region | 1.03 | (0.93, 1.14) | 0.052 | .543 |

*Survey-weighted logistic regression estimates of factors associated with Hib vaccine coverage, NIS-Child 2020*.

## **Hib Vaccine Results 2021**

| Predictor | Odds Ratio | 95% CI | SE | P value |
| --- | --- | --- | --- | --- |
| (Intercept) | 7.88 | (1.00, 61.86) | 1.052 | .050 |
| Age Group | 1.13 | (0.97, 1.31) | 0.078 | .127 |
| Household size | 0.95 | (0.86, 1.05) | 0.053 | .339 |
| Breastfeeding History | 1.04 | (0.71, 1.52) | 0.192 | .835 |
| WIC benefits | 0.67 | (0.49, 0.90) | 0.153 | .008 |
| Maternal Education | 1.17 | (1.01, 1.35) | 0.076 | .041 |
| Firstborn | 1.66 | (1.21, 2.26) | 0.159 | .002 |
| Hispanic Ethnicity | 0.92 | (0.64, 1.32) | 0.186 | .651 |
| Income | 1.43 | (1.20, 1.71) | 0.091 | <.001 |
| Language | 1.38 | (0.68, 2.79) | 0.359 | .369 |
| Maternal Age | 1.45 | (1.09, 1.92) | 0.143 | .010 |
| Marital Status | 0.74 | (0.54, 1.00) | 0.154 | .048 |
| Racial Identity | 0.88 | (0.75, 1.02) | 0.077 | .087 |
| Sex | 1.21 | (0.94, 1.57) | 0.130 | .136 |
| Insurance Status | 0.60 | (0.48, 0.74) | 0.113 | <.001 |
| Region | 0.93 | (0.82, 1.07) | 0.067 | .316 |

*Survey-weighted logistic regression estimates of factors associated with Hib vaccine coverage, NIS-Child 2021*.

## **Hib Vaccine Results 2022**

| Predictor | Odds Ratio | 95% CI | SE | P value |
| --- | --- | --- | --- | --- |
| (Intercept) | 18.86 | (2.54, 139.86) | 1.022 | .004 |
| Age Group | 1.12 | (0.97, 1.30) | 0.073 | .112 |
| Household size | 0.88 | (0.81, 0.97) | 0.047 | .009 |
| Breastfeeding History | 1.04 | (0.73, 1.48) | 0.180 | .816 |
| WIC benefits | 0.80 | (0.57, 1.11) | 0.170 | .184 |
| Maternal Education | 1.30 | (1.12, 1.50) | 0.075 | <.001 |
| Firstborn | 1.19 | (0.88, 1.61) | 0.154 | .256 |
| Hispanic Ethnicity | 0.63 | (0.45, 0.88) | 0.169 | .006 |
| Income | 1.19 | (0.93, 1.53) | 0.127 | .172 |
| Language | 0.86 | (0.60, 1.23) | 0.183 | .412 |
| Maternal Age | 1.37 | (1.04, 1.80) | 0.139 | .023 |
| Marital Status | 1.10 | (0.79, 1.53) | 0.168 | .572 |
| Racial Identity | 0.87 | (0.76, 1.01) | 0.071 | .061 |
| Sex | 0.99 | (0.78, 1.25) | 0.120 | .930 |
| Insurance Status | 0.74 | (0.63, 0.88) | 0.085 | <.001 |
| Region | 0.99 | (0.88, 1.12) | 0.061 | .920 |

*Survey-weighted logistic regression estimates of factors associated with Hib vaccine coverage, NIS-Child 2022*.

## **Hib Vaccine Results 2023**

| Predictor | Odds Ratio | 95% CI | SE | P value |
| --- | --- | --- | --- | --- |
| (Intercept) | 122.54 | (27.45, 546.95) | 0.763 | <.001 |
| Age Group | 0.98 | (0.85, 1.12) | 0.071 | .721 |
| Household size | 0.88 | (0.79, 0.98) | 0.056 | .023 |
| Breastfeeding History | 0.75 | (0.55, 1.01) | 0.155 | .059 |
| WIC benefits | 0.63 | (0.48, 0.83) | 0.139 | <.001 |
| Maternal Education | 1.18 | (1.03, 1.34) | 0.066 | .014 |
| Firstborn | 1.05 | (0.81, 1.36) | 0.133 | .740 |
| Hispanic Ethnicity | 0.71 | (0.54, 0.94) | 0.142 | .017 |
| Income | 1.27 | (1.06, 1.52) | 0.094 | .011 |
| Language | 0.91 | (0.65, 1.28) | 0.173 | .583 |
| Maternal Age | 1.23 | (0.96, 1.58) | 0.128 | .102 |
| Marital Status | 0.88 | (0.68, 1.13) | 0.128 | .303 |
| Racial Identity | 1.03 | (0.90, 1.18) | 0.069 | .701 |
| Sex | 0.95 | (0.76, 1.18) | 0.112 | .622 |
| Insurance Status | 0.68 | (0.59, 0.79) | 0.077 | <.001 |
| Region | 0.83 | (0.74, 0.93) | 0.057 | <.001 |

*Survey-weighted logistic regression estimates of factors associated with Hib vaccine coverage, NIS-Child 2023*.

## **Influenza Vaccine Results 2010**

| Predictor | Odds Ratio | 95% CI | SE | P value |
| --- | --- | --- | --- | --- |
| (Intercept) | 2.13 | (0.82, 5.53) | 0.486 | .119 |
| Age Group | 1.07 | (0.99, 1.15) | 0.038 | .093 |
| Household size | 0.93 | (0.89, 0.98) | 0.026 | .009 |
| Breastfeeding History | 0.92 | (0.80, 1.07) | 0.073 | .287 |
| WIC benefits | 1.04 | (0.85, 1.28) | 0.103 | .681 |
| Maternal Education | 1.14 | (1.05, 1.23) | 0.039 | .001 |
| Firstborn | 1.03 | (0.90, 1.18) | 0.070 | .695 |
| Hispanic Ethnicity | 0.68 | (0.57, 0.82) | 0.093 | <.001 |
| Income | 1.10 | (0.99, 1.22) | 0.052 | .066 |
| Language | 1.23 | (1.00, 1.53) | 0.109 | .052 |
| Maternal Age | 1.20 | (1.05, 1.37) | 0.067 | .007 |
| Marital Status | 1.07 | (0.91, 1.27) | 0.086 | .422 |
| Racial Identity | 1.09 | (1.00, 1.20) | 0.048 | .060 |
| Sex | 0.97 | (0.86, 1.10) | 0.062 | .676 |
| Insurance Status | 0.83 | (0.75, 0.91) | 0.047 | <.001 |
| Region | 0.90 | (0.84, 0.95) | 0.032 | <.001 |

*Survey-weighted logistic regression estimates of factors associated with Influenza vaccine coverage, NIS-Child 2010*.

## **Influenza Vaccine Results 2011**

| Predictor | Odds Ratio | 95% CI | SE | P value |
| --- | --- | --- | --- | --- |
| (Intercept) | 3.95 | (1.52, 10.28) | 0.488 | .005 |
| Age Group | 1.10 | (1.02, 1.19) | 0.038 | .010 |
| Household size | 0.91 | (0.87, 0.96) | 0.027 | <.001 |
| Breastfeeding History | 0.85 | (0.73, 0.99) | 0.077 | .033 |
| WIC benefits | 1.06 | (0.86, 1.29) | 0.103 | .594 |
| Maternal Education | 1.02 | (0.94, 1.11) | 0.041 | .570 |
| Firstborn | 0.95 | (0.81, 1.11) | 0.079 | .513 |
| Hispanic Ethnicity | 0.79 | (0.66, 0.95) | 0.092 | .011 |
| Income | 1.22 | (1.09, 1.36) | 0.055 | <.001 |
| Language | 1.64 | (1.30, 2.06) | 0.117 | <.001 |
| Maternal Age | 0.99 | (0.86, 1.13) | 0.070 | .861 |
| Marital Status | 0.89 | (0.76, 1.05) | 0.082 | .154 |
| Racial Identity | 1.12 | (1.02, 1.22) | 0.046 | .018 |
| Sex | 0.97 | (0.86, 1.09) | 0.061 | .621 |
| Insurance Status | 0.85 | (0.77, 0.93) | 0.046 | <.001 |
| Region | 0.87 | (0.82, 0.92) | 0.030 | <.001 |

*Survey-weighted logistic regression estimates of factors associated with Influenza vaccine coverage, NIS-Child 2011*.

## **Influenza Vaccine Results 2012**

| Predictor | Odds Ratio | 95% CI | SE | P value |
| --- | --- | --- | --- | --- |
| (Intercept) | 0.91 | (0.34, 2.46) | 0.506 | .852 |
| Age Group | 1.09 | (1.01, 1.18) | 0.041 | .030 |
| Household size | 0.94 | (0.89, 1.00) | 0.029 | .051 |
| Breastfeeding History | 1.06 | (0.90, 1.25) | 0.083 | .481 |
| WIC benefits | 1.07 | (0.87, 1.32) | 0.108 | .531 |
| Maternal Education | 1.18 | (1.08, 1.28) | 0.043 | <.001 |
| Firstborn | 1.18 | (1.00, 1.40) | 0.087 | .051 |
| Hispanic Ethnicity | 0.79 | (0.65, 0.97) | 0.104 | .027 |
| Income | 1.12 | (1.00, 1.25) | 0.057 | .053 |
| Language | 1.37 | (1.09, 1.72) | 0.116 | .006 |
| Maternal Age | 1.12 | (0.97, 1.29) | 0.074 | .135 |
| Marital Status | 1.02 | (0.86, 1.21) | 0.086 | .780 |
| Racial Identity | 1.15 | (1.06, 1.26) | 0.045 | .001 |
| Sex | 0.99 | (0.86, 1.13) | 0.068 | .853 |
| Insurance Status | 0.90 | (0.82, 0.98) | 0.045 | .019 |
| Region | 0.87 | (0.81, 0.93) | 0.035 | <.001 |

*Survey-weighted logistic regression estimates of factors associated with Influenza vaccine coverage, NIS-Child 2012*.

## **Influenza Vaccine Results 2013**

| Predictor | Odds Ratio | 95% CI | SE | P value |
| --- | --- | --- | --- | --- |
| (Intercept) | 2.41 | (0.76, 7.66) | 0.590 | .136 |
| Age Group | 1.08 | (0.98, 1.18) | 0.047 | .125 |
| Household size | 0.92 | (0.86, 0.98) | 0.033 | .009 |
| Breastfeeding History | 1.00 | (0.82, 1.22) | 0.102 | .988 |
| WIC benefits | 0.99 | (0.78, 1.24) | 0.118 | .904 |
| Maternal Education | 1.08 | (0.99, 1.19) | 0.048 | .099 |
| Firstborn | 1.14 | (0.95, 1.37) | 0.094 | .168 |
| Hispanic Ethnicity | 0.79 | (0.62, 0.99) | 0.120 | .045 |
| Income | 1.08 | (0.96, 1.22) | 0.060 | .183 |
| Language | 1.51 | (1.14, 2.00) | 0.143 | .004 |
| Maternal Age | 1.30 | (1.10, 1.55) | 0.088 | .003 |
| Marital Status | 0.88 | (0.72, 1.07) | 0.101 | .196 |
| Racial Identity | 1.09 | (0.98, 1.22) | 0.056 | .112 |
| Sex | 0.94 | (0.81, 1.09) | 0.077 | .416 |
| Insurance Status | 0.81 | (0.73, 0.89) | 0.050 | <.001 |
| Region | 0.86 | (0.79, 0.92) | 0.038 | <.001 |

*Survey-weighted logistic regression estimates of factors associated with Influenza vaccine coverage, NIS-Child 2013*.

## **Influenza Vaccine Results 2014**

| Predictor | OR | 95% CI | SE | p |
| --- | --- | --- | --- | --- |
| (Intercept) | 1.56 | (0.50, 4.88) | 0.583 | .447 |
| Age Group | 1.15 | (1.05, 1.26) | 0.047 | .003 |
| Household size | 0.90 | (0.84, 0.97) | 0.034 | .003 |
| Breastfeeding History | 0.96 | (0.79, 1.16) | 0.098 | .686 |
| WIC benefits | 0.94 | (0.75, 1.19) | 0.120 | .635 |
| Maternal Education | 1.04 | (0.94, 1.15) | 0.050 | .436 |
| Firstborn | 0.95 | (0.79, 1.15) | 0.096 | .624 |
| Hispanic Ethnicity | 0.73 | (0.58, 0.94) | 0.124 | .013 |
| Income | 1.42 | (1.24, 1.62) | 0.067 | <.001 |
| Language | 1.84 | (1.39, 2.43) | 0.143 | <.001 |
| Maternal Age | 1.14 | (0.96, 1.36) | 0.091 | .144 |
| Marital Status | 1.06 | (0.86, 1.29) | 0.103 | .594 |
| Racial Identity | 1.15 | (1.03, 1.28) | 0.056 | .013 |
| Sex | 1.12 | (0.96, 1.31) | 0.080 | .147 |
| Insurance Status | 0.86 | (0.77, 0.96) | 0.058 | .008 |
| Region | 0.83 | (0.77, 0.90) | 0.041 | <.001 |

*Survey-weighted logistic regression estimates of factors associated with Influenza vaccine coverage, NIS-Child 2014*.

## **Influenza Vaccine Results 2015**

| Predictor | OR | 95% CI | SE | p |
| --- | --- | --- | --- | --- |
| (Intercept) | 1.27 | (0.38, 4.21) | 0.611 | .696 |
| Age Group | 1.14 | (1.05, 1.25) | 0.045 | .003 |
| Household size | 0.98 | (0.92, 1.04) | 0.032 | .511 |
| Breastfeeding History | 0.81 | (0.67, 0.97) | 0.094 | .024 |
| WIC benefits | 0.82 | (0.63, 1.06) | 0.131 | .123 |
| Maternal Education | 1.13 | (1.02, 1.24) | 0.050 | .016 |
| Firstborn | 1.31 | (1.09, 1.57) | 0.091 | .003 |
| Hispanic Ethnicity | 0.87 | (0.69, 1.08) | 0.114 | .209 |
| Income | 1.27 | (1.13, 1.43) | 0.060 | <.001 |
| Language | 1.58 | (1.24, 2.02) | 0.124 | <.001 |
| Maternal Age | 1.19 | (1.01, 1.40) | 0.084 | .039 |
| Marital Status | 1.04 | (0.86, 1.27) | 0.100 | .688 |
| Racial Identity | 1.11 | (1.00, 1.24) | 0.053 | .047 |
| Sex | 0.85 | (0.74, 0.98) | 0.073 | .027 |
| Insurance Status | 0.79 | (0.71, 0.87) | 0.050 | <.001 |
| Region | 0.90 | (0.84, 0.97) | 0.037 | .007 |

*Survey-weighted logistic regression estimates of factors associated with Influenza vaccine coverage, NIS-Child 2015*.

## **Influenza Vaccine Results 2016**

| Predictor | Odds Ratio | 95% CI | SE | P value |
| --- | --- | --- | --- | --- |
| (Intercept) | 2.78 | (0.90, 8.57) | 0.574 | .075 |
| Age Group | 1.12 | (1.03, 1.22) | 0.044 | .009 |
| Household size | 0.88 | (0.83, 0.94) | 0.031 | <.001 |
| Breastfeeding History | 0.91 | (0.75, 1.10) | 0.099 | .332 |
| WIC benefits | 0.92 | (0.72, 1.17) | 0.124 | .504 |
| Maternal Education | 1.05 | (0.95, 1.16) | 0.050 | .352 |
| Firstborn | 1.18 | (0.98, 1.42) | 0.094 | .083 |
| Hispanic Ethnicity | 0.79 | (0.64, 0.98) | 0.108 | .032 |
| Income | 1.23 | (1.08, 1.39) | 0.066 | .002 |
| Language | 1.50 | (1.18, 1.90) | 0.123 | .001 |
| Maternal Age | 1.34 | (1.12, 1.59) | 0.090 | .001 |
| Marital Status | 1.03 | (0.85, 1.26) | 0.101 | .750 |
| Racial Identity | 1.07 | (0.96, 1.19) | 0.055 | .219 |
| Sex | 0.95 | (0.82, 1.11) | 0.076 | .541 |
| Insurance Status | 0.74 | (0.65, 0.85) | 0.071 | <.001 |
| Region | 0.85 | (0.79, 0.92) | 0.038 | <.001 |

*Survey-weighted logistic regression estimates of factors associated with Influenza vaccine coverage, NIS-Child 2016*.

## **Influenza Vaccine Results 2017**

| Predictor | Odds Ratio | 95% CI | SE | P value |
| --- | --- | --- | --- | --- |
| (Intercept) | 1.35 | (0.46, 3.90) | 0.543 | .585 |
| Age Group | 1.07 | (0.99, 1.17) | 0.043 | .103 |
| Household size | 0.95 | (0.88, 1.01) | 0.035 | .104 |
| Breastfeeding History | 1.01 | (0.83, 1.23) | 0.101 | .906 |
| WIC benefits | 0.90 | (0.71, 1.14) | 0.119 | .381 |
| Maternal Education | 1.16 | (1.06, 1.27) | 0.045 | <.001 |
| Firstborn | 1.21 | (1.01, 1.45) | 0.091 | .038 |
| Hispanic Ethnicity | 0.70 | (0.58, 0.86) | 0.101 | <.001 |
| Income | 1.23 | (1.09, 1.39) | 0.060 | <.001 |
| Language | 1.87 | (1.49, 2.34) | 0.116 | <.001 |
| Maternal Age | 1.30 | (1.11, 1.54) | 0.084 | .002 |
| Marital Status | 0.89 | (0.74, 1.07) | 0.094 | .213 |
| Racial Identity | 1.05 | (0.95, 1.17) | 0.052 | .320 |
| Sex | 0.98 | (0.84, 1.13) | 0.074 | .745 |
| Insurance Status | 0.78 | (0.71, 0.86) | 0.047 | <.001 |
| Region | 0.93 | (0.86, 1.01) | 0.040 | .075 |

*Survey-weighted logistic regression estimates of factors associated with Influenza vaccine coverage, NIS-Child 2017.*

## **Influenza Vaccine Results 2018**

| Predictor | Odds Ratio | 95% CI | SE | P value |
| --- | --- | --- | --- | --- |
| (Intercept) | 1.91 | (0.59, 6.22) | 0.601 | .280 |
| Age Group | 1.11 | (1.00, 1.23) | 0.052 | .048 |
| Household size | 0.94 | (0.87, 1.01) | 0.039 | .089 |
| Breastfeeding History | 0.93 | (0.75, 1.16) | 0.113 | .528 |
| WIC benefits | 1.00 | (0.81, 1.23) | 0.109 | .983 |
| Maternal Education | 1.08 | (0.97, 1.20) | 0.054 | .174 |
| Firstborn | 1.31 | (1.08, 1.58) | 0.097 | .006 |
| Hispanic Ethnicity | 0.73 | (0.59, 0.90) | 0.110 | .004 |
| Income | 1.25 | (1.10, 1.41) | 0.062 | <.001 |
| Language | 1.60 | (1.22, 2.09) | 0.138 | <.001 |
| Maternal Age | 1.36 | (1.14, 1.63) | 0.093 | <.001 |
| Marital Status | 0.80 | (0.65, 0.98) | 0.105 | .034 |
| Racial Identity | 1.01 | (0.90, 1.13) | 0.058 | .845 |
| Sex | 1.04 | (0.89, 1.21) | 0.080 | .635 |
| Insurance Status | 0.77 | (0.69, 0.84) | 0.050 | <.001 |
| Region | 0.91 | (0.84, 0.99) | 0.043 | .027 |

*Survey-weighted logistic regression estimates of factors associated with Influenza vaccine coverage, NIS-Child 2018*.

**Influenza Vaccine Results 2019**

| Predictor | Odds Ratio | 95% CI | SE | P value |
| --- | --- | --- | --- | --- |
| (Intercept) | 1.53 | (0.47, 5.00) | 0.605 | .485 |
| Age Group | 1.01 | (0.91, 1.11) | 0.052 | .905 |
| Household size | 0.96 | (0.89, 1.03) | 0.035 | .237 |
| Breastfeeding History | 0.93 | (0.75, 1.16) | 0.112 | .532 |
| WIC benefits | 1.10 | (0.86, 1.42) | 0.128 | .450 |
| Maternal Education | 1.24 | (1.11, 1.39) | 0.057 | <.001 |
| Firstborn | 1.41 | (1.16, 1.72) | 0.102 | <.001 |
| Hispanic Ethnicity | 0.80 | (0.63, 1.01) | 0.122 | .064 |
| Income | 1.13 | (1.00, 1.29) | 0.065 | .053 |
| Language | 2.10 | (1.54, 2.87) | 0.158 | <.001 |
| Maternal Age | 1.15 | (0.94, 1.40) | 0.102 | .181 |
| Marital Status | 0.89 | (0.72, 1.09) | 0.106 | .260 |
| Racial Identity | 0.85 | (0.76, 0.95) | 0.056 | .004 |
| Sex | 0.87 | (0.74, 1.03) | 0.084 | .098 |
| Insurance Status | 0.80 | (0.72, 0.90) | 0.057 | <.001 |
| Region | 0.91 | (0.83, 1.00) | 0.045 | .042 |

*Survey-weighted logistic regression estimates of factors associated with Influenza vaccine coverage, NIS-Child 2019.*

## **Influenza Vaccine Results 2020**

| Predictor | Odds Ratio | 95% CI | SE | P value |
| --- | --- | --- | --- | --- |
| (Intercept) | 1.52 | (0.56, 4.14) | 0.512 | .415 |
| Age Group | 1.03 | (0.95, 1.12) | 0.042 | .511 |
| Household size | 0.98 | (0.92, 1.04) | 0.031 | .482 |
| Breastfeeding History | 1.06 | (0.87, 1.29) | 0.100 | .577 |
| WIC benefits | 0.99 | (0.81, 1.21) | 0.101 | .944 |
| Maternal Education | 1.14 | (1.04, 1.25) | 0.048 | .006 |
| Firstborn | 1.49 | (1.26, 1.77) | 0.086 | <.001 |
| Hispanic Ethnicity | 0.82 | (0.68, 0.99) | 0.095 | .036 |
| Income | 1.25 | (1.13, 1.40) | 0.054 | <.001 |
| Language | 1.54 | (1.17, 2.01) | 0.138 | .002 |
| Maternal Age | 1.23 | (1.05, 1.45) | 0.084 | .012 |
| Marital Status | 0.76 | (0.64, 0.90) | 0.087 | .001 |
| Racial Identity | 1.02 | (0.94, 1.11) | 0.042 | .680 |
| Sex | 0.98 | (0.86, 1.13) | 0.070 | .810 |
| Insurance Status | 0.78 | (0.70, 0.87) | 0.055 | <.001 |
| Region | 0.91 | (0.86, 0.98) | 0.033 | .007 |

*Survey-weighted logistic regression estimates of factors associated with Influenza vaccine coverage, NIS-Child 2020.*

## **Influenza Vaccine Results 2021**

| Predictor | Odds Ratio | 95% CI | SE | P value |
| --- | --- | --- | --- | --- |
| (Intercept) | 1.90 | (0.56, 6.41) | 0.620 | .299 |
| Age Group | 1.15 | (1.05, 1.27) | 0.050 | .004 |
| Household size | 0.97 | (0.91, 1.04) | 0.035 | .423 |
| Breastfeeding History | 1.06 | (0.83, 1.34) | 0.122 | .652 |
| WIC benefits | 0.97 | (0.78, 1.21) | 0.111 | .785 |
| Maternal Education | 1.17 | (1.05, 1.30) | 0.055 | .005 |
| Firstborn | 1.16 | (0.96, 1.40) | 0.096 | .118 |
| Hispanic Ethnicity | 0.75 | (0.60, 0.93) | 0.113 | .010 |
| Income | 1.19 | (1.05, 1.35) | 0.065 | .007 |
| Language | 1.60 | (1.08, 2.37) | 0.200 | .019 |
| Maternal Age | 1.40 | (1.17, 1.68) | 0.092 | <.001 |
| Marital Status | 0.65 | (0.54, 0.79) | 0.100 | <.001 |
| Racial Identity | 0.98 | (0.89, 1.08) | 0.050 | .690 |
| Sex | 1.11 | (0.95, 1.29) | 0.080 | .203 |
| Insurance Status | 0.87 | (0.76, 0.99) | 0.068 | .039 |
| Region | 0.87 | (0.80, 0.94) | 0.039 | <.001 |

*Survey-weighted logistic regression estimates of factors associated with Influenza vaccine coverage, NIS-Child 2021*.

## **Influenza Vaccine Results 2022**

| Predictor | Odds Ratio | 95% CI | SE | P value |
| --- | --- | --- | --- | --- |
| (Intercept) | 2.90 | (0.93, 9.02) | 0.578 | .065 |
| Age Group | 1.22 | (1.11, 1.33) | 0.046 | <.001 |
| Household size | 0.93 | (0.87, 0.99) | 0.032 | .027 |
| Breastfeeding History | 0.82 | (0.67, 1.01) | 0.103 | .061 |
| WIC benefits | 1.04 | (0.85, 1.28) | 0.104 | .676 |
| Maternal Education | 1.20 | (1.09, 1.31) | 0.047 | <.001 |
| Firstborn | 1.22 | (1.02, 1.45) | 0.090 | .029 |
| Hispanic Ethnicity | 0.73 | (0.59, 0.89) | 0.103 | .002 |
| Income | 1.13 | (1.01, 1.27) | 0.059 | .035 |
| Language | 1.49 | (1.13, 1.98) | 0.144 | .005 |
| Maternal Age | 1.29 | (1.09, 1.54) | 0.089 | .004 |
| Marital Status | 0.59 | (0.49, 0.72) | 0.094 | <.001 |
| Racial Identity | 1.02 | (0.93, 1.11) | 0.045 | .656 |
| Sex | 0.92 | (0.79, 1.06) | 0.074 | .250 |
| Insurance Status | 0.78 | (0.69, 0.87) | 0.060 | <.001 |
| Region | 0.99 | (0.92, 1.06) | 0.035 | .705 |

*Survey-weighted logistic regression estimates of factors associated with Influenza vaccine coverage, NIS-Child 2022*.

## **Influenza Vaccine Results 2023**

| Predictor | Odds Ratio | 95% CI | SE | P value |
| --- | --- | --- | --- | --- |
| (Intercept) | 1.11 | (0.38, 3.21) | 0.543 | .854 |
| Age Group | 1.21 | (1.11, 1.32) | 0.044 | <.001 |
| Household size | 0.93 | (0.87, 0.99) | 0.034 | .032 |
| Breastfeeding History | 0.99 | (0.80, 1.23) | 0.109 | .962 |
| WIC benefits | 1.05 | (0.86, 1.28) | 0.101 | .606 |
| Maternal Education | 1.28 | (1.16, 1.40) | 0.048 | <.001 |
| Firstborn | 1.12 | (0.94, 1.33) | 0.088 | .211 |
| Hispanic Ethnicity | 0.79 | (0.65, 0.96) | 0.100 | .018 |
| Income | 1.22 | (1.09, 1.36) | 0.057 | <.001 |
| Language | 2.03 | (1.51, 2.72) | 0.149 | <.001 |
| Maternal Age | 1.19 | (1.00, 1.41) | 0.089 | .051 |
| Marital Status | 0.74 | (0.62, 0.88) | 0.090 | <.001 |
| Racial Identity | 1.03 | (0.95, 1.12) | 0.044 | .484 |
| Sex | 1.11 | (0.96, 1.27) | 0.072 | .158 |
| Insurance Status | 0.70 | (0.64, 0.78) | 0.052 | <.001 |
| Region | 0.84 | (0.78, 0.90) | 0.036 | <.001 |

*Survey-weighted logistic regression estimates of factors associated with Influenza vaccine coverage, NIS-Child 2023.*

## **MMR Vaccine Results 2010**

| Predictor | Odds Ratio | 95% CI | SE | P value |
| --- | --- | --- | --- | --- |
| (Intercept) | 8.53 | (1.78, 40.89) | 0.800 | .007 |
| Age Group | 1.38 | (1.21, 1.58) | 0.067 | <.001 |
| Household size | 0.86 | (0.79, 0.93) | 0.041 | <.001 |
| Breastfeeding History | 1.16 | (0.91, 1.48) | 0.123 | .232 |
| WIC benefits | 0.60 | (0.44, 0.81) | 0.158 | .001 |
| Maternal Education | 1.08 | (0.94, 1.23) | 0.067 | .271 |
| Firstborn | 1.14 | (0.90, 1.44) | 0.119 | .265 |
| Hispanic Ethnicity | 0.85 | (0.62, 1.16) | 0.161 | .299 |
| Income | 1.08 | (0.91, 1.28) | 0.088 | .391 |
| Language | 2.02 | (1.30, 3.15) | 0.227 | .002 |
| Maternal Age | 1.19 | (0.97, 1.47) | 0.105 | .095 |
| Marital Status | 1.13 | (0.87, 1.46) | 0.132 | .368 |
| Racial Identity | 0.98 | (0.84, 1.15) | 0.080 | .806 |
| Sex | 0.87 | (0.71, 1.07) | 0.103 | .180 |
| Insurance Status | 0.85 | (0.73, 0.98) | 0.073 | .023 |
| Region | 0.96 | (0.85, 1.08) | 0.059 | .460 |

*Survey-weighted logistic regression estimates of factors associated with MMR vaccine coverage, NIS-Child 2010*.

## **MMR Vaccine Results 2011**

| Predictor | Odds Ratio | 95% CI | SE | P value |
| --- | --- | --- | --- | --- |
| (Intercept) | 17.43 | (3.87, 78.41) | 0.767 | <.001 |
| Age Group | 1.29 | (1.14, 1.45) | 0.063 | <.001 |
| Household size | 0.91 | (0.84, 0.98) | 0.039 | .015 |
| Breastfeeding History | 1.05 | (0.81, 1.37) | 0.134 | .715 |
| WIC benefits | 0.58 | (0.41, 0.83) | 0.177 | .002 |
| Maternal Education | 0.95 | (0.85, 1.08) | 0.062 | .451 |
| Firstborn | 1.27 | (0.99, 1.62) | 0.126 | .062 |
| Hispanic Ethnicity | 0.80 | (0.56, 1.13) | 0.177 | .203 |
| Income | 1.22 | (1.03, 1.46) | 0.090 | .025 |
| Language | 1.47 | (1.02, 2.11) | 0.185 | .038 |
| Maternal Age | 1.09 | (0.89, 1.33) | 0.103 | .411 |
| Marital Status | 1.03 | (0.77, 1.38) | 0.149 | .859 |
| Racial Identity | 1.08 | (0.91, 1.29) | 0.090 | .388 |
| Sex | 1.10 | (0.90, 1.34) | 0.101 | .359 |
| Insurance Status | 0.70 | (0.61, 0.80) | 0.067 | <.001 |
| Region | 0.97 | (0.87, 1.07) | 0.055 | .521 |

*Survey-weighted logistic regression estimates of factors associated with MMR vaccine coverage, NIS-Child 2011*.

## **MMR Vaccine Results 2012**

| Predictor | Odds Ratio | 95% CI | SE | P value |
| --- | --- | --- | --- | --- |
| (Intercept) | 54.32 | (11.94, 247.09) | 0.773 | <.001 |
| Age Group | 1.20 | (1.04, 1.39) | 0.073 | .011 |
| Household size | 0.88 | (0.81, 0.96) | 0.044 | .005 |
| Breastfeeding History | 1.23 | (0.93, 1.62) | 0.141 | .145 |
| WIC benefits | 0.62 | (0.45, 0.86) | 0.166 | .004 |
| Maternal Education | 1.17 | (1.02, 1.34) | 0.069 | .024 |
| Firstborn | 1.15 | (0.87, 1.52) | 0.140 | .314 |
| Hispanic Ethnicity | 0.74 | (0.52, 1.07) | 0.186 | .111 |
| Income | 1.06 | (0.88, 1.27) | 0.093 | .561 |
| Language | 1.14 | (0.76, 1.72) | 0.207 | .520 |
| Maternal Age | 0.81 | (0.62, 1.04) | 0.132 | .102 |
| Marital Status | 0.79 | (0.61, 1.03) | 0.134 | .078 |
| Racial Identity | 1.00 | (0.85, 1.17) | 0.083 | .957 |
| Sex | 1.11 | (0.89, 1.38) | 0.110 | .351 |
| Insurance Status | 0.77 | (0.69, 0.87) | 0.061 | <.001 |
| Region | 0.96 | (0.85, 1.08) | 0.060 | .460 |

*Survey-weighted logistic regression estimates of factors associated with MMR vaccine coverage, NIS-Child 2012.*

## **MMR Vaccine Results 2013**

| Predictor | Odds Ratio | 95% CI | SE | P value |
| --- | --- | --- | --- | --- |
| (Intercept) | 9.00 | (1.33, 60.82) | 0.975 | .024 |
| Age Group | 1.36 | (1.16, 1.60) | 0.082 | <.001 |
| Household size | 0.92 | (0.81, 1.04) | 0.063 | .163 |
| Breastfeeding History | 1.30 | (0.94, 1.82) | 0.170 | .117 |
| WIC benefits | 0.63 | (0.44, 0.90) | 0.180 | .011 |
| Maternal Education | 1.26 | (1.08, 1.46) | 0.076 | .003 |
| Firstborn | 1.25 | (0.89, 1.74) | 0.169 | .194 |
| Hispanic Ethnicity | 0.76 | (0.51, 1.14) | 0.206 | .190 |
| Income | 0.99 | (0.82, 1.21) | 0.101 | .958 |
| Language | 1.92 | (0.98, 3.74) | 0.341 | .056 |
| Maternal Age | 1.29 | (0.94, 1.77) | 0.161 | .111 |
| Marital Status | 0.73 | (0.54, 0.99) | 0.157 | .046 |
| Racial Identity | 1.26 | (1.05, 1.52) | 0.094 | .015 |
| Sex | 0.98 | (0.75, 1.28) | 0.135 | .897 |
| Insurance Status | 0.68 | (0.57, 0.80) | 0.084 | <.001 |
| Region | 0.84 | (0.73, 0.96) | 0.072 | .014 |

*Survey-weighted logistic regression estimates of factors associated with MMR vaccine coverage, NIS-Child 2013*.

## **MMR Vaccine Results 2014**

| Predictor | Odds Ratio | 95% CI | SE | P value |
| --- | --- | --- | --- | --- |
| (Intercept) | 3.07 | (0.43, 21.85) | 1.002 | .263 |
| Age Group | 1.41 | (1.20, 1.66) | 0.082 | <.001 |
| Household size | 0.91 | (0.82, 1.00) | 0.049 | .042 |
| Breastfeeding History | 1.34 | (0.99, 1.81) | 0.153 | .057 |
| WIC benefits | 0.96 | (0.64, 1.46) | 0.212 | .857 |
| Maternal Education | 1.09 | (0.93, 1.29) | 0.084 | .296 |
| Firstborn | 1.43 | (1.08, 1.90) | 0.146 | .014 |
| Hispanic Ethnicity | 0.66 | (0.43, 1.00) | 0.215 | .049 |
| Income | 1.30 | (1.04, 1.63) | 0.115 | .023 |
| Language | 1.56 | (0.96, 2.54) | 0.249 | .075 |
| Maternal Age | 1.10 | (0.81, 1.49) | 0.156 | .538 |
| Marital Status | 1.45 | (1.04, 2.02) | 0.170 | .029 |
| Racial Identity | 1.13 | (0.93, 1.37) | 0.098 | .205 |
| Sex | 1.04 | (0.81, 1.34) | 0.128 | .733 |
| Insurance Status | 0.75 | (0.63, 0.90) | 0.089 | .001 |
| Region | 0.84 | (0.74, 0.95) | 0.065 | .006 |

*Survey-weighted logistic regression estimates of factors associated with MMR vaccine coverage, NIS-Child 2014*.

## **MMR Vaccine Results 2015**

| Predictor | Odds Ratio | 95% CI | SE | P value |
| --- | --- | --- | --- | --- |
| (Intercept) | 18.93 | (3.85, 93.19) | 0.813 | <.001 |
| Age Group | 1.22 | (1.07, 1.41) | 0.071 | .004 |
| Household size | 0.88 | (0.80, 0.97) | 0.048 | .011 |
| Breastfeeding History | 1.13 | (0.83, 1.53) | 0.155 | .428 |
| WIC benefits | 0.80 | (0.56, 1.14) | 0.184 | .219 |
| Maternal Education | 1.19 | (1.02, 1.39) | 0.079 | .028 |
| Firstborn | 1.56 | (1.15, 2.11) | 0.154 | .004 |
| Hispanic Ethnicity | 0.69 | (0.50, 0.95) | 0.163 | .024 |
| Income | 1.01 | (0.85, 1.22) | 0.093 | .881 |
| Language | 1.20 | (0.83, 1.72) | 0.185 | .335 |
| Maternal Age | 1.18 | (0.88, 1.57) | 0.146 | .265 |
| Marital Status | 0.70 | (0.50, 0.96) | 0.165 | .028 |
| Racial Identity | 1.09 | (0.91, 1.30) | 0.092 | .361 |
| Sex | 1.13 | (0.89, 1.45) | 0.125 | .320 |
| Insurance Status | 0.65 | (0.56, 0.77) | 0.082 | <.001 |
| Region | 0.99 | (0.87, 1.13) | 0.068 | .884 |

*Survey-weighted logistic regression estimates of factors associated with MMR vaccine coverage, NIS-Child 2015*.

## **MMR Vaccine Results 2016**

| Predictor | Odds Ratio | 95% CI | SE | P value |
| --- | --- | --- | --- | --- |
| (Intercept) | 9.64 | (1.43, 65.20) | 0.975 | .020 |
| Age Group | 1.29 | (1.10, 1.50) | 0.078 | .001 |
| Household size | 0.94 | (0.85, 1.04) | 0.051 | .249 |
| Breastfeeding History | 0.98 | (0.70, 1.36) | 0.171 | .885 |
| WIC benefits | 0.64 | (0.40, 1.03) | 0.241 | .064 |
| Maternal Education | 1.36 | (1.13, 1.63) | 0.094 | .001 |
| Firstborn | 0.95 | (0.71, 1.26) | 0.145 | .711 |
| Hispanic Ethnicity | 0.81 | (0.55, 1.20) | 0.199 | .288 |
| Income | 1.21 | (0.93, 1.57) | 0.135 | .162 |
| Language | 1.67 | (1.05, 2.68) | 0.240 | .032 |
| Maternal Age | 1.09 | (0.84, 1.43) | 0.135 | .511 |
| Marital Status | 1.18 | (0.85, 1.64) | 0.166 | .313 |
| Racial Identity | 1.03 | (0.86, 1.23) | 0.094 | .769 |
| Sex | 1.06 | (0.82, 1.37) | 0.130 | .662 |
| Insurance Status | 0.72 | (0.59, 0.88) | 0.101 | .001 |
| Region | 0.80 | (0.70, 0.92) | 0.069 | .001 |

*Survey-weighted logistic regression estimates of factors associated with MMR vaccine coverage, NIS-Child 2016*.

## **MMR Vaccine Results 2017**

| Predictor | Odds Ratio | 95% CI | SE | P value |
| --- | --- | --- | --- | --- |
| (Intercept) | 3.94 | (0.87, 17.93) | 0.773 | .076 |
| Age Group | 1.30 | (1.12, 1.52) | 0.078 | <.001 |
| Household size | 0.90 | (0.82, 0.98) | 0.045 | .016 |
| Breastfeeding History | 1.33 | (0.95, 1.85) | 0.170 | .098 |
| WIC benefits | 0.53 | (0.34, 0.83) | 0.230 | .005 |
| Maternal Education | 1.39 | (1.19, 1.62) | 0.079 | <.001 |
| Firstborn | 1.13 | (0.86, 1.49) | 0.142 | .386 |
| Hispanic Ethnicity | 0.78 | (0.55, 1.11) | 0.180 | .161 |
| Income | 1.36 | (1.10, 1.68) | 0.108 | .005 |
| Language | 1.37 | (0.96, 1.94) | 0.180 | .082 |
| Maternal Age | 1.45 | (1.15, 1.84) | 0.121 | .002 |
| Marital Status | 1.12 | (0.85, 1.47) | 0.142 | .437 |
| Racial Identity | 0.96 | (0.80, 1.15) | 0.092 | .681 |
| Sex | 1.07 | (0.84, 1.36) | 0.124 | .582 |
| Insurance Status | 0.79 | (0.66, 0.95) | 0.094 | .013 |
| Region | 0.94 | (0.83, 1.06) | 0.063 | .337 |

*Survey-weighted logistic regression estimates of factors associated with MMR vaccine coverage, NIS-Child 2017*.

## **MMR Vaccine Results 2018**

| Predictor | Odds Ratio | 95% CI | SE | P value |
| --- | --- | --- | --- | --- |
| (Intercept) | 32.36 | (4.13, 253.46) | 1.050 | <.001 |
| Age Group | 1.37 | (1.20, 1.57) | 0.069 | <.001 |
| Household size | 0.85 | (0.75, 0.98) | 0.069 | .021 |
| Breastfeeding History | 1.12 | (0.81, 1.55) | 0.166 | .482 |
| WIC benefits | 0.87 | (0.54, 1.42) | 0.248 | .584 |
| Maternal Education | 1.04 | (0.86, 1.25) | 0.096 | .690 |
| Firstborn | 1.12 | (0.78, 1.60) | 0.182 | .534 |
| Hispanic Ethnicity | 0.66 | (0.45, 0.97) | 0.197 | .036 |
| Income | 1.10 | (0.91, 1.33) | 0.098 | .321 |
| Language | 1.43 | (0.93, 2.22) | 0.223 | .105 |
| Maternal Age | 1.08 | (0.80, 1.46) | 0.154 | .622 |
| Marital Status | 0.80 | (0.57, 1.13) | 0.174 | .210 |
| Racial Identity | 1.10 | (0.92, 1.32) | 0.093 | .291 |
| Sex | 1.16 | (0.89, 1.53) | 0.139 | .276 |
| Insurance Status | 0.66 | (0.58, 0.76) | 0.068 | <.001 |
| Region | 0.89 | (0.75, 1.06) | 0.086 | .185 |

*Survey-weighted logistic regression estimates of factors associated with MMR vaccine coverage, NIS-Child 2018*.

**MMR Vaccine Results 2019**

| Predictor | Odds Ratio | 95% CI | SE | P value |
| --- | --- | --- | --- | --- |
| (Intercept) | 2.48 | (0.33, 18.77) | 1.032 | .378 |
| Age Group | 1.16 | (0.98, 1.38) | 0.089 | .093 |
| Household size | 0.93 | (0.81, 1.07) | 0.069 | .311 |
| Breastfeeding History | 1.97 | (1.36, 2.87) | 0.190 | <.001 |
| WIC benefits | 0.73 | (0.48, 1.11) | 0.211 | .142 |
| Maternal Education | 1.30 | (1.06, 1.60) | 0.106 | .014 |
| Firstborn | 1.04 | (0.70, 1.55) | 0.204 | .846 |
| Hispanic Ethnicity | 1.17 | (0.75, 1.84) | 0.231 | .489 |
| Income | 1.24 | (0.99, 1.55) | 0.113 | .057 |
| Language | 1.98 | (1.23, 3.21) | 0.245 | .005 |
| Maternal Age | 1.22 | (0.88, 1.69) | 0.166 | .236 |
| Marital Status | 0.97 | (0.67, 1.41) | 0.190 | .865 |
| Racial Identity | 0.70 | (0.56, 0.86) | 0.109 | <.001 |
| Sex | 0.88 | (0.65, 1.17) | 0.149 | .376 |
| Insurance Status | 0.88 | (0.71, 1.08) | 0.108 | .228 |
| Region | 0.90 | (0.77, 1.06) | 0.080 | .200 |

*Survey-weighted logistic regression estimates of factors associated with MMR vaccine coverage, NIS-Child 2019*.

## **MMR Vaccine Results 2020**

| Predictor | Odds Ratio | 95% CI | SE | P value |
| --- | --- | --- | --- | --- |
| (Intercept) | 5.73 | (0.83, 39.70) | 0.987 | .077 |
| Age Group | 1.33 | (1.15, 1.55) | 0.077 | <.001 |
| Household size | 0.91 | (0.83, 0.99) | 0.047 | .033 |
| Breastfeeding History | 1.02 | (0.76, 1.36) | 0.149 | .918 |
| WIC benefits | 0.93 | (0.67, 1.29) | 0.169 | .653 |
| Maternal Education | 1.07 | (0.92, 1.25) | 0.077 | .354 |
| Firstborn | 1.44 | (1.04, 1.99) | 0.164 | .027 |
| Hispanic Ethnicity | 0.99 | (0.71, 1.39) | 0.172 | .952 |
| Income | 1.18 | (0.98, 1.43) | 0.097 | .088 |
| Language | 1.39 | (0.83, 2.34) | 0.264 | .209 |
| Maternal Age | 1.38 | (1.05, 1.80) | 0.139 | .021 |
| Marital Status | 0.94 | (0.71, 1.24) | 0.142 | .640 |
| Racial Identity | 0.90 | (0.79, 1.03) | 0.068 | .122 |
| Sex | 1.04 | (0.83, 1.31) | 0.116 | .730 |
| Insurance Status | 0.73 | (0.63, 0.85) | 0.074 | <.001 |
| Region | 0.95 | (0.86, 1.06) | 0.055 | .393 |

*Survey-weighted logistic regression estimates of factors associated with MMR vaccine coverage, NIS-Child 2020*.

## **MMR Vaccine Results 2021**

| Predictor | Odds Ratio | 95% CI | SE | P value |
| --- | --- | --- | --- | --- |
| (Intercept) | 13.91 | (1.71, 113.17) | 1.070 | .014 |
| Age Group | 1.41 | (1.20, 1.65) | 0.082 | <.001 |
| Household size | 0.90 | (0.80, 1.00) | 0.058 | .058 |
| Breastfeeding History | 0.99 | (0.65, 1.51) | 0.216 | .961 |
| WIC benefits | 0.77 | (0.55, 1.08) | 0.170 | .128 |
| Maternal Education | 1.17 | (0.99, 1.39) | 0.085 | .058 |
| Firstborn | 1.00 | (0.72, 1.37) | 0.163 | .988 |
| Hispanic Ethnicity | 0.91 | (0.63, 1.31) | 0.186 | .617 |
| Income | 1.23 | (1.01, 1.49) | 0.099 | .035 |
| Language | 1.21 | (0.62, 2.38) | 0.345 | .579 |
| Maternal Age | 1.52 | (1.13, 2.05) | 0.153 | .006 |
| Marital Status | 0.72 | (0.52, 0.99) | 0.163 | .043 |
| Racial Identity | 0.97 | (0.83, 1.13) | 0.077 | .677 |
| Sex | 1.05 | (0.81, 1.36) | 0.132 | .721 |
| Insurance Status | 0.75 | (0.62, 0.90) | 0.098 | .003 |
| Region | 0.86 | (0.76, 0.98) | 0.066 | .022 |

*Survey-weighted logistic regression estimates of factors associated with MMR vaccine coverage, NIS-Child 2021.*

## **MMR Vaccine Results 2022**

| Predictor | Odds Ratio | 95% CI | SE | P value |
| --- | --- | --- | --- | --- |
| (Intercept) | 24.01 | (2.49, 231.93) | 1.157 | .006 |
| Age Group | 1.34 | (1.14, 1.57) | 0.081 | <.001 |
| Household size | 0.87 | (0.78, 0.96) | 0.052 | .007 |
| Breastfeeding History | 0.85 | (0.58, 1.24) | 0.193 | .387 |
| WIC benefits | 0.72 | (0.49, 1.05) | 0.194 | .092 |
| Maternal Education | 1.20 | (1.03, 1.41) | 0.080 | .020 |
| Firstborn | 1.05 | (0.76, 1.45) | 0.164 | .768 |
| Hispanic Ethnicity | 0.77 | (0.54, 1.08) | 0.175 | .130 |
| Income | 1.10 | (0.84, 1.44) | 0.137 | .489 |
| Language | 1.45 | (0.90, 2.36) | 0.248 | .130 |
| Maternal Age | 1.16 | (0.86, 1.57) | 0.153 | .323 |
| Marital Status | 0.94 | (0.65, 1.34) | 0.184 | .721 |
| Racial Identity | 0.93 | (0.80, 1.08) | 0.078 | .336 |
| Sex | 1.08 | (0.85, 1.39) | 0.126 | .528 |
| Insurance Status | 0.72 | (0.60, 0.85) | 0.091 | <.001 |
| Region | 1.00 | (0.88, 1.13) | 0.064 | .957 |

*Survey-weighted logistic regression estimates of factors associated with MMR vaccine coverage, NIS-Child 2022*.

## **MMR Vaccine Results 2023**

| Predictor | Odds Ratio | 95% CI | SE | P value |
| --- | --- | --- | --- | --- |
| (Intercept) | 77.49 | (14.87, 403.85) | 0.842 | <.001 |
| Age Group | 1.12 | (0.97, 1.31) | 0.076 | .124 |
| Household size | 0.84 | (0.74, 0.96) | 0.065 | .009 |
| Breastfeeding History | 0.76 | (0.54, 1.07) | 0.174 | .116 |
| WIC benefits | 0.83 | (0.62, 1.11) | 0.151 | .213 |
| Maternal Education | 1.20 | (1.04, 1.37) | 0.069 | .010 |
| Firstborn | 0.82 | (0.62, 1.08) | 0.140 | .152 |
| Hispanic Ethnicity | 0.83 | (0.60, 1.14) | 0.163 | .251 |
| Income | 1.07 | (0.87, 1.31) | 0.105 | .541 |
| Language | 1.34 | (0.78, 2.30) | 0.277 | .295 |
| Maternal Age | 1.12 | (0.85, 1.48) | 0.143 | .421 |
| Marital Status | 0.86 | (0.66, 1.12) | 0.136 | .258 |
| Racial Identity | 1.01 | (0.88, 1.16) | 0.071 | .875 |
| Sex | 0.94 | (0.75, 1.19) | 0.117 | .628 |
| Insurance Status | 0.76 | (0.66, 0.87) | 0.069 | <.001 |
| Region | 0.82 | (0.72, 0.93) | 0.064 | .002 |

*Survey-weighted logistic regression estimates of factors associated with MMR vaccine coverage, NIS-Child 2023*.

## **PCV Results 2010**

| Predictor | Odds Ratio | 95% CI | SE | P value |
| --- | --- | --- | --- | --- |
| (Intercept) | 5.58 | (1.65, 18.82) | 0.621 | .006 |
| Age Group | 1.08 | (0.98, 1.19) | 0.048 | .101 |
| Household size | 0.88 | (0.83, 0.94) | 0.031 | <.001 |
| Breastfeeding History | 0.88 | (0.74, 1.06) | 0.094 | .195 |
| WIC benefits | 0.64 | (0.49, 0.82) | 0.132 | <.001 |
| Maternal Education | 1.15 | (1.05, 1.27) | 0.048 | .003 |
| Firstborn | 1.13 | (0.95, 1.35) | 0.090 | .168 |
| Hispanic Ethnicity | 0.95 | (0.75, 1.21) | 0.123 | .688 |
| Income | 1.25 | (1.09, 1.43) | 0.069 | .001 |
| Language | 1.78 | (1.33, 2.39) | 0.149 | <.001 |
| Maternal Age | 1.03 | (0.87, 1.21) | 0.085 | .768 |
| Marital Status | 0.95 | (0.78, 1.16) | 0.102 | .644 |
| Racial Identity | 0.89 | (0.80, 0.99) | 0.054 | .031 |
| Sex | 1.12 | (0.97, 1.31) | 0.078 | .132 |
| Insurance Status | 0.84 | (0.76, 0.94) | 0.053 | .001 |
| Region | 0.98 | (0.90, 1.07) | 0.044 | .608 |

*Survey-weighted logistic regression estimates of factors associated with PCV vaccine coverage, NIS-Child 2010*.

## **PCV Results 2011**

| Predictor | Odds Ratio | 95% CI | SE | P value |
| --- | --- | --- | --- | --- |
| (Intercept) | 2.41 | (0.77, 7.53) | 0.581 | .130 |
| Age Group | 1.18 | (1.08, 1.30) | 0.047 | <.001 |
| Household size | 0.97 | (0.91, 1.03) | 0.033 | .298 |
| Breastfeeding History | 0.91 | (0.75, 1.09) | 0.094 | .291 |
| WIC benefits | 0.66 | (0.51, 0.86) | 0.132 | .002 |
| Maternal Education | 1.07 | (0.96, 1.19) | 0.054 | .232 |
| Firstborn | 1.60 | (1.32, 1.94) | 0.099 | <.001 |
| Hispanic Ethnicity | 0.92 | (0.72, 1.17) | 0.125 | .499 |
| Income | 1.33 | (1.17, 1.52) | 0.068 | <.001 |
| Language | 1.40 | (1.07, 1.84) | 0.139 | .016 |
| Maternal Age | 1.22 | (1.05, 1.43) | 0.079 | .012 |
| Marital Status | 0.99 | (0.81, 1.22) | 0.104 | .951 |
| Racial Identity | 0.87 | (0.77, 0.98) | 0.060 | .021 |
| Sex | 1.03 | (0.88, 1.20) | 0.079 | .737 |
| Insurance Status | 0.78 | (0.70, 0.87) | 0.054 | <.001 |
| Region | 0.99 | (0.92, 1.08) | 0.041 | .888 |

*Survey-weighted logistic regression estimates of factors associated with PCV vaccine coverage, NIS-Child 2011*.

## **PCV Results 2012**

| Predictor | Odds Ratio | 95% CI | SE | P value |
| --- | --- | --- | --- | --- |
| (Intercept) | 9.09 | (2.80, 29.51) | 0.601 | <.001 |
| Age Group | 1.16 | (1.05, 1.28) | 0.051 | .004 |
| Household size | 0.88 | (0.82, 0.95) | 0.036 | <.001 |
| Breastfeeding History | 1.00 | (0.82, 1.21) | 0.098 | .990 |
| WIC benefits | 0.69 | (0.53, 0.88) | 0.127 | .003 |
| Maternal Education | 1.14 | (1.04, 1.26) | 0.050 | .008 |
| Firstborn | 1.28 | (1.03, 1.60) | 0.114 | .029 |
| Hispanic Ethnicity | 0.71 | (0.56, 0.90) | 0.118 | .004 |
| Income | 1.25 | (1.09, 1.44) | 0.071 | .001 |
| Language | 0.98 | (0.78, 1.23) | 0.117 | .847 |
| Maternal Age | 1.15 | (0.97, 1.37) | 0.088 | .109 |
| Marital Status | 0.96 | (0.78, 1.17) | 0.103 | .690 |
| Racial Identity | 0.91 | (0.82, 1.00) | 0.052 | .061 |
| Sex | 0.95 | (0.81, 1.12) | 0.083 | .521 |
| Insurance Status | 0.79 | (0.71, 0.87) | 0.049 | <.001 |
| Region | 1.02 | (0.93, 1.11) | 0.045 | .735 |

*Survey-weighted logistic regression estimates of factors associated with PCV vaccine coverage, NIS-Child 2012*.

## **PCV Results 2013**

| Predictor | Odds Ratio | 95% CI | SE | P value |
| --- | --- | --- | --- | --- |
| (Intercept) | 2.96 | (0.79, 11.11) | 0.675 | .108 |
| Age Group | 1.15 | (1.02, 1.28) | 0.057 | .017 |
| Household size | 0.96 | (0.89, 1.04) | 0.040 | .358 |
| Breastfeeding History | 0.92 | (0.74, 1.15) | 0.113 | .470 |
| WIC benefits | 0.83 | (0.63, 1.10) | 0.142 | .196 |
| Maternal Education | 1.19 | (1.06, 1.34) | 0.059 | .003 |
| Firstborn | 1.45 | (1.15, 1.82) | 0.117 | .001 |
| Hispanic Ethnicity | 0.78 | (0.59, 1.03) | 0.143 | .075 |
| Income | 1.29 | (1.10, 1.51) | 0.079 | .001 |
| Language | 1.14 | (0.85, 1.52) | 0.150 | .397 |
| Maternal Age | 1.22 | (0.99, 1.50) | 0.105 | .056 |
| Marital Status | 0.99 | (0.79, 1.24) | 0.116 | .918 |
| Racial Identity | 0.99 | (0.88, 1.13) | 0.064 | .905 |
| Sex | 1.08 | (0.89, 1.30) | 0.096 | .442 |
| Insurance Status | 0.70 | (0.62, 0.79) | 0.060 | <.001 |
| Region | 0.89 | (0.81, 0.98) | 0.049 | .019 |

*Survey-weighted logistic regression estimates of factors associated with PCV vaccine coverage, NIS-Child 2013*.

## **PCV Results 2014**

| Predictor | Odds Ratio | 95% CI | SE | P value |
| --- | --- | --- | --- | --- |
| (Intercept) | 4.18 | (0.97, 17.94) | 0.743 | .054 |
| Age Group | 1.19 | (1.06, 1.34) | 0.062 | .005 |
| Household size | 0.90 | (0.83, 0.97) | 0.038 | .004 |
| Breastfeeding History | 0.93 | (0.74, 1.18) | 0.120 | .551 |
| WIC benefits | 0.79 | (0.58, 1.07) | 0.155 | .126 |
| Maternal Education | 1.07 | (0.95, 1.21) | 0.062 | .241 |
| Firstborn | 1.07 | (0.84, 1.34) | 0.119 | .592 |
| Hispanic Ethnicity | 0.74 | (0.55, 0.99) | 0.151 | .042 |
| Income | 1.43 | (1.22, 1.69) | 0.083 | <.001 |
| Language | 1.14 | (0.85, 1.53) | 0.149 | .369 |
| Maternal Age | 1.32 | (1.05, 1.66) | 0.117 | .018 |
| Marital Status | 1.18 | (0.92, 1.52) | 0.128 | .184 |
| Racial Identity | 0.92 | (0.81, 1.05) | 0.067 | .234 |
| Sex | 1.08 | (0.90, 1.31) | 0.098 | .407 |
| Insurance Status | 0.80 | (0.70, 0.92) | 0.069 | .002 |
| Region | 0.91 | (0.82, 1.00) | 0.050 | .046 |

*Survey-weighted logistic regression estimates of factors associated with PCV vaccine coverage, NIS-Child 2014*.

## **PCV Results 2015**

| Predictor | Odds Ratio | 95% CI | SE | P value |
| --- | --- | --- | --- | --- |
| (Intercept) | 6.26 | (2.05, 19.07) | 0.568 | .001 |
| Age Group | 1.08 | (0.97, 1.20) | 0.053 | .147 |
| Household size | 0.92 | (0.86, 0.98) | 0.035 | .014 |
| Breastfeeding History | 0.97 | (0.79, 1.20) | 0.107 | .794 |
| WIC benefits | 0.57 | (0.42, 0.77) | 0.151 | <.001 |
| Maternal Education | 1.08 | (0.97, 1.20) | 0.055 | .161 |
| Firstborn | 1.47 | (1.20, 1.80) | 0.103 | <.001 |
| Hispanic Ethnicity | 0.78 | (0.61, 1.00) | 0.126 | .054 |
| Income | 1.32 | (1.13, 1.55) | 0.081 | <.001 |
| Language | 0.93 | (0.76, 1.15) | 0.107 | .518 |
| Maternal Age | 1.51 | (1.25, 1.82) | 0.097 | <.001 |
| Marital Status | 0.95 | (0.76, 1.19) | 0.113 | .651 |
| Racial Identity | 0.92 | (0.82, 1.04) | 0.062 | .201 |
| Sex | 1.01 | (0.85, 1.20) | 0.086 | .884 |
| Insurance Status | 0.73 | (0.65, 0.81) | 0.056 | <.001 |
| Region | 0.98 | (0.90, 1.07) | 0.045 | .696 |

*Survey-weighted logistic regression estimates of factors associated with PCV vaccine coverage, NIS-Child 2015*.

## **PCV Results 2016**

| Predictor | Odds Ratio | 95% CI | SE | P value |
| --- | --- | --- | --- | --- |
| (Intercept) | 6.69 | (1.76, 25.38) | 0.680 | .005 |
| Age Group | 1.11 | (1.00, 1.24) | 0.054 | .051 |
| Household size | 0.93 | (0.86, 1.01) | 0.039 | .073 |
| Breastfeeding History | 0.94 | (0.73, 1.20) | 0.127 | .607 |
| WIC benefits | 0.71 | (0.51, 0.99) | 0.172 | .044 |
| Maternal Education | 1.15 | (1.01, 1.29) | 0.062 | .030 |
| Firstborn | 1.40 | (1.12, 1.76) | 0.115 | .003 |
| Hispanic Ethnicity | 0.86 | (0.66, 1.13) | 0.139 | .293 |
| Income | 1.17 | (0.98, 1.40) | 0.091 | .088 |
| Language | 0.99 | (0.76, 1.30) | 0.138 | .948 |
| Maternal Age | 1.33 | (1.05, 1.68) | 0.119 | .016 |
| Marital Status | 0.92 | (0.71, 1.19) | 0.130 | .525 |
| Racial Identity | 0.97 | (0.85, 1.11) | 0.069 | .657 |
| Sex | 1.04 | (0.86, 1.27) | 0.098 | .660 |
| Insurance Status | 0.73 | (0.63, 0.85) | 0.079 | <.001 |
| Region | 0.87 | (0.79, 0.96) | 0.051 | .005 |

*Survey-weighted logistic regression estimates of factors associated with PCV vaccine coverage, NIS-Child 2016*

## **PCV Results 2017**

| Predictor | Odds Ratio | 95% CI | SE | P value |
| --- | --- | --- | --- | --- |
| (Intercept) | 8.22 | (2.21, 30.55) | 0.670 | .002 |
| Age Group | 1.13 | (1.01, 1.25) | 0.055 | .030 |
| Household size | 0.90 | (0.84, 0.97) | 0.038 | .008 |
| Breastfeeding History | 0.88 | (0.69, 1.13) | 0.127 | .313 |
| WIC benefits | 0.54 | (0.40, 0.72) | 0.149 | <.001 |
| Maternal Education | 1.20 | (1.07, 1.34) | 0.058 | .002 |
| Firstborn | 1.30 | (1.04, 1.63) | 0.116 | .024 |
| Hispanic Ethnicity | 0.92 | (0.69, 1.22) | 0.145 | .554 |
| Income | 1.38 | (1.19, 1.60) | 0.076 | <.001 |
| Language | 1.11 | (0.87, 1.41) | 0.124 | .405 |
| Maternal Age | 1.24 | (1.03, 1.51) | 0.098 | .026 |
| Marital Status | 0.84 | (0.64, 1.11) | 0.138 | .219 |
| Racial Identity | 0.87 | (0.77, 0.98) | 0.061 | .020 |
| Sex | 1.03 | (0.86, 1.24) | 0.093 | .739 |
| Insurance Status | 0.79 | (0.70, 0.90) | 0.061 | <.001 |
| Region | 0.93 | (0.84, 1.03) | 0.053 | .172 |

*Survey-weighted logistic regression estimates of factors associated with PCV vaccine coverage, NIS-Child 2017*.

## **PCV Results 2018**

| Predictor | Odds Ratio | 95% CI | SE | P value |
| --- | --- | --- | --- | --- |
| (Intercept) | 7.24 | (1.75, 29.96) | 0.725 | .006 |
| Age Group | 1.26 | (1.12, 1.41) | 0.058 | <.001 |
| Household size | 0.85 | (0.78, 0.93) | 0.046 | <.001 |
| Breastfeeding History | 0.93 | (0.72, 1.20) | 0.130 | .570 |
| WIC benefits | 0.87 | (0.63, 1.19) | 0.161 | .385 |
| Maternal Education | 1.24 | (1.08, 1.43) | 0.072 | .003 |
| Firstborn | 1.14 | (0.86, 1.50) | 0.140 | .360 |
| Hispanic Ethnicity | 0.90 | (0.67, 1.20) | 0.148 | .470 |
| Income | 1.15 | (0.98, 1.35) | 0.082 | .095 |
| Language | 1.43 | (1.05, 1.94) | 0.157 | .024 |
| Maternal Age | 1.20 | (0.95, 1.51) | 0.118 | .128 |
| Marital Status | 0.81 | (0.63, 1.06) | 0.132 | .120 |
| Racial Identity | 0.94 | (0.82, 1.07) | 0.066 | .333 |
| Sex | 0.97 | (0.80, 1.18) | 0.099 | .750 |
| Insurance Status | 0.73 | (0.65, 0.82) | 0.063 | <.001 |
| Region | 0.89 | (0.80, 0.99) | 0.055 | .029 |

*Survey-weighted logistic regression estimates of factors associated with PCV vaccine coverage, NIS-Child 2018*.

## **PCV Results 2019**

| Predictor | Odds Ratio | 95% CI | SE | P value |
| --- | --- | --- | --- | --- |
| (Intercept) | 3.88 | (0.91, 16.56) | 0.741 | .068 |
| Age Group | 1.06 | (0.93, 1.20) | 0.063 | .376 |
| Household size | 0.88 | (0.81, 0.96) | 0.044 | .005 |
| Breastfeeding History | 1.29 | (1.00, 1.66) | 0.129 | .047 |
| WIC benefits | 0.81 | (0.58, 1.14) | 0.170 | .227 |
| Maternal Education | 1.18 | (1.03, 1.36) | 0.072 | .020 |
| Firstborn | 1.40 | (1.07, 1.83) | 0.137 | .014 |
| Hispanic Ethnicity | 0.99 | (0.73, 1.36) | 0.161 | .969 |
| Income | 1.24 | (1.05, 1.47) | 0.085 | .011 |
| Language | 1.44 | (1.07, 1.94) | 0.152 | .017 |
| Maternal Age | 1.31 | (1.05, 1.63) | 0.112 | .017 |
| Marital Status | 0.84 | (0.63, 1.11) | 0.141 | .211 |
| Racial Identity | 0.74 | (0.64, 0.85) | 0.074 | <.001 |
| Sex | 0.91 | (0.74, 1.11) | 0.105 | .343 |
| Insurance Status | 0.80 | (0.70, 0.92) | 0.070 | .002 |
| Region | 0.93 | (0.83, 1.05) | 0.059 | .247 |

*Survey-weighted logistic regression estimates of factors associated with PCV vaccine coverage, NIS-Child 2019*.

## **PCV Results 2020**

| Predictor | Odds Ratio | 95% CI | SE | P value |
| --- | --- | --- | --- | --- |
| (Intercept) | 3.26 | (0.99, 10.74) | 0.609 | .052 |
| Age Group | 1.17 | (1.05, 1.29) | 0.053 | .004 |
| Household size | 0.93 | (0.86, 0.99) | 0.035 | .025 |
| Breastfeeding History | 0.94 | (0.76, 1.17) | 0.109 | .585 |
| WIC benefits | 0.78 | (0.61, 1.00) | 0.126 | .052 |
| Maternal Education | 1.11 | (1.00, 1.25) | 0.057 | .057 |
| Firstborn | 1.31 | (1.05, 1.62) | 0.110 | .015 |
| Hispanic Ethnicity | 1.07 | (0.86, 1.32) | 0.110 | .555 |
| Income | 1.35 | (1.19, 1.54) | 0.067 | <.001 |
| Language | 1.26 | (0.94, 1.68) | 0.146 | .116 |
| Maternal Age | 1.35 | (1.11, 1.63) | 0.099 | .003 |
| Marital Status | 0.87 | (0.71, 1.06) | 0.103 | .160 |
| Racial Identity | 0.86 | (0.78, 0.95) | 0.051 | .004 |
| Sex | 0.90 | (0.77, 1.06) | 0.081 | .198 |
| Insurance Status | 0.76 | (0.67, 0.85) | 0.059 | <.001 |
| Region | 0.99 | (0.92, 1.08) | 0.040 | .890 |

*Survey-weighted logistic regression estimates of factors associated with PCV vaccine coverage, NIS-Child 2020*.

## **PCV Results 2021**

| Predictor | Odds Ratio | 95% CI | SE | P value |
| --- | --- | --- | --- | --- |
| (Intercept) | 6.60 | (1.61, 27.06) | 0.720 | .009 |
| Age Group | 1.24 | (1.10, 1.40) | 0.060 | <.001 |
| Household size | 0.96 | (0.89, 1.04) | 0.040 | .318 |
| Breastfeeding History | 1.02 | (0.78, 1.33) | 0.137 | .893 |
| WIC benefits | 0.85 | (0.66, 1.10) | 0.129 | .220 |
| Maternal Education | 1.19 | (1.06, 1.33) | 0.059 | .004 |
| Firstborn | 1.54 | (1.21, 1.98) | 0.126 | <.001 |
| Hispanic Ethnicity | 0.76 | (0.59, 0.97) | 0.128 | .028 |
| Income | 1.18 | (1.02, 1.38) | 0.077 | .030 |
| Language | 1.14 | (0.78, 1.67) | 0.195 | .503 |
| Maternal Age | 1.30 | (1.05, 1.60) | 0.107 | .014 |
| Marital Status | 0.70 | (0.55, 0.89) | 0.123 | .004 |
| Racial Identity | 0.96 | (0.86, 1.07) | 0.058 | .458 |
| Sex | 0.90 | (0.75, 1.08) | 0.094 | .244 |
| Insurance Status | 0.64 | (0.56, 0.74) | 0.074 | <.001 |
| Region | 0.94 | (0.85, 1.04) | 0.050 | .227 |

*Survey-weighted logistic regression estimates of factors associated with PCV vaccine coverage, NIS-Child 2021*.

## **PCV Results 2022**

| Predictor | Odds Ratio | 95% CI | SE | P value |
| --- | --- | --- | --- | --- |
| (Intercept) | 10.49 | (2.55, 43.16) | 0.722 | .001 |
| Age Group | 1.18 | (1.06, 1.32) | 0.057 | .004 |
| Household size | 0.84 | (0.78, 0.92) | 0.042 | <.001 |
| Breastfeeding History | 0.95 | (0.74, 1.23) | 0.131 | .717 |
| WIC benefits | 0.77 | (0.60, 0.99) | 0.129 | .044 |
| Maternal Education | 1.24 | (1.10, 1.39) | 0.059 | <.001 |
| Firstborn | 1.20 | (0.95, 1.52) | 0.120 | .131 |
| Hispanic Ethnicity | 0.79 | (0.62, 1.00) | 0.122 | .054 |
| Income | 1.28 | (1.09, 1.51) | 0.084 | .003 |
| Language | 1.19 | (0.88, 1.62) | 0.157 | .256 |
| Maternal Age | 1.22 | (0.99, 1.50) | 0.108 | .067 |
| Marital Status | 0.82 | (0.64, 1.03) | 0.121 | .090 |
| Racial Identity | 0.87 | (0.78, 0.97) | 0.057 | .015 |
| Sex | 1.04 | (0.87, 1.25) | 0.093 | .657 |
| Insurance Status | 0.70 | (0.61, 0.80) | 0.072 | <.001 |
| Region | 0.96 | (0.87, 1.05) | 0.046 | .361 |

*Survey-weighted logistic regression estimates of factors associated with PCV vaccine coverage, NIS-Child 2022*.

## **PCV Results 2023**

| Predictor | Odds Ratio | 95% CI | SE | P value |
| --- | --- | --- | --- | --- |
| (Intercept) | 13.33 | (4.16, 42.75) | 0.595 | <.001 |
| Age Group | 1.13 | (1.02, 1.26) | 0.055 | .024 |
| Household size | 0.86 | (0.79, 0.93) | 0.041 | <.001 |
| Breastfeeding History | 0.86 | (0.67, 1.11) | 0.128 | .256 |
| WIC benefits | 0.84 | (0.68, 1.04) | 0.110 | .117 |
| Maternal Education | 1.20 | (1.08, 1.33) | 0.053 | <.001 |
| Firstborn | 0.96 | (0.77, 1.18) | 0.108 | .678 |
| Hispanic Ethnicity | 0.88 | (0.71, 1.10) | 0.110 | .262 |
| Income | 1.32 | (1.15, 1.51) | 0.069 | <.001 |
| Language | 0.95 | (0.73, 1.23) | 0.131 | .688 |
| Maternal Age | 1.21 | (0.99, 1.49) | 0.104 | .063 |
| Marital Status | 0.86 | (0.70, 1.06) | 0.105 | .150 |
| Racial Identity | 1.00 | (0.90, 1.11) | 0.053 | .994 |
| Sex | 1.01 | (0.85, 1.20) | 0.088 | .924 |
| Insurance Status | 0.73 | (0.64, 0.83) | 0.066 | <.001 |
| Region | 0.85 | (0.77, 0.93) | 0.047 | <.001 |

*Survey-weighted logistic regression estimates of factors associated with PCV vaccine coverage, NIS-Child 2023*.

## **Polio Vaccine Results 2010**

| Predictor | Odds Ratio | 95% CI | SE | P value |
| --- | --- | --- | --- | --- |
| (Intercept) | 17.08 | (3.31, 88.23) | 0.838 | <.001 |
| Age Group | 1.19 | (1.03, 1.38) | 0.074 | .017 |
| Household size | 0.84 | (0.77, 0.91) | 0.044 | <.001 |
| Breastfeeding History | 1.12 | (0.86, 1.47) | 0.137 | .396 |
| WIC benefits | 0.54 | (0.38, 0.75) | 0.171 | <.001 |
| Maternal Education | 1.08 | (0.93, 1.25) | 0.076 | .303 |
| Firstborn | 0.90 | (0.70, 1.16) | 0.128 | .417 |
| Hispanic Ethnicity | 0.92 | (0.64, 1.31) | 0.181 | .636 |
| Income | 1.24 | (1.04, 1.48) | 0.089 | .014 |
| Language | 1.85 | (1.16, 2.94) | 0.236 | .009 |
| Maternal Age | 1.17 | (0.92, 1.49) | 0.125 | .212 |
| Marital Status | 1.27 | (0.97, 1.66) | 0.136 | .083 |
| Racial Identity | 0.89 | (0.75, 1.05) | 0.084 | .164 |
| Sex | 1.12 | (0.90, 1.41) | 0.116 | .311 |
| Insurance Status | 0.85 | (0.74, 0.99) | 0.076 | .039 |
| Region | 0.88 | (0.77, 1.01) | 0.068 | .062 |

*Survey-weighted logistic regression estimates of factors associated with Polio vaccine coverage, NIS-Child 2010*.

## **Polio Vaccine Results 2011**

| Predictor | Odds Ratio | 95% CI | SE | P value |
| --- | --- | --- | --- | --- |
| (Intercept) | 6.25 | (1.18, 33.18) | 0.852 | .031 |
| Age Group | 1.04 | (0.92, 1.18) | 0.064 | .549 |
| Household size | 0.94 | (0.86, 1.03) | 0.046 | .219 |
| Breastfeeding History | 1.10 | (0.82, 1.47) | 0.150 | .534 |
| WIC benefits | 0.56 | (0.38, 0.83) | 0.202 | .004 |
| Maternal Education | 0.97 | (0.84, 1.14) | 0.078 | .737 |
| Firstborn | 1.49 | (1.15, 1.93) | 0.131 | .002 |
| Hispanic Ethnicity | 1.24 | (0.88, 1.77) | 0.179 | .222 |
| Income | 1.19 | (0.98, 1.46) | 0.102 | .085 |
| Language | 2.28 | (1.51, 3.45) | 0.212 | <.001 |
| Maternal Age | 1.22 | (0.99, 1.51) | 0.108 | .064 |
| Marital Status | 1.05 | (0.77, 1.45) | 0.163 | .751 |
| Racial Identity | 0.90 | (0.76, 1.08) | 0.090 | .253 |
| Sex | 1.13 | (0.91, 1.40) | 0.111 | .283 |
| Insurance Status | 0.70 | (0.60, 0.80) | 0.073 | <.001 |
| Region | 0.94 | (0.84, 1.06) | 0.059 | .317 |

*Survey-weighted logistic regression estimates of factors associated with Polio vaccine coverage, NIS-Child 2011*.

## **Polio Vaccine Results 2012**

| Predictor | Odds Ratio | 95% CI | SE | P value |
| --- | --- | --- | --- | --- |
| (Intercept) | 37.59 | (8.78, 161.02) | 0.742 | <.001 |
| Age Group | 1.12 | (0.97, 1.29) | 0.073 | .120 |
| Household size | 0.87 | (0.80, 0.95) | 0.043 | .001 |
| Breastfeeding History | 1.06 | (0.80, 1.41) | 0.145 | .690 |
| WIC benefits | 0.49 | (0.32, 0.73) | 0.211 | <.001 |
| Maternal Education | 1.20 | (1.05, 1.38) | 0.070 | .008 |
| Firstborn | 1.00 | (0.76, 1.33) | 0.142 | .985 |
| Hispanic Ethnicity | 0.92 | (0.67, 1.28) | 0.167 | .631 |
| Income | 1.14 | (0.93, 1.41) | 0.106 | .206 |
| Language | 1.30 | (0.89, 1.89) | 0.194 | .181 |
| Maternal Age | 0.98 | (0.75, 1.28) | 0.136 | .892 |
| Marital Status | 0.88 | (0.68, 1.14) | 0.133 | .318 |
| Racial Identity | 0.99 | (0.86, 1.14) | 0.073 | .869 |
| Sex | 1.13 | (0.90, 1.42) | 0.116 | .287 |
| Insurance Status | 0.76 | (0.67, 0.86) | 0.063 | <.001 |
| Region | 0.99 | (0.87, 1.11) | 0.062 | .824 |

*Survey-weighted logistic regression estimates of factors associated with Polio vaccine coverage, NIS-Child 2012*.

## **Polio Vaccine Results 2013**

| Predictor | Odds Ratio | 95% CI | SE | P value |
| --- | --- | --- | --- | --- |
| (Intercept) | 12.71 | (1.86, 86.61) | 0.979 | .009 |
| Age Group | 1.17 | (0.99, 1.38) | 0.086 | .070 |
| Household size | 0.94 | (0.83, 1.06) | 0.061 | .301 |
| Breastfeeding History | 1.20 | (0.84, 1.72) | 0.183 | .324 |
| WIC benefits | 0.68 | (0.48, 0.97) | 0.181 | .032 |
| Maternal Education | 1.29 | (1.09, 1.51) | 0.083 | .002 |
| Firstborn | 1.42 | (1.00, 2.02) | 0.179 | .049 |
| Hispanic Ethnicity | 0.84 | (0.57, 1.22) | 0.194 | .356 |
| Income | 1.12 | (0.90, 1.40) | 0.114 | .320 |
| Language | 1.38 | (0.81, 2.33) | 0.269 | .236 |
| Maternal Age | 1.17 | (0.82, 1.67) | 0.182 | .384 |
| Marital Status | 0.73 | (0.51, 1.04) | 0.182 | .084 |
| Racial Identity | 1.05 | (0.86, 1.29) | 0.104 | .634 |
| Sex | 1.04 | (0.78, 1.38) | 0.147 | .811 |
| Insurance Status | 0.68 | (0.57, 0.82) | 0.093 | <.001 |
| Region | 0.86 | (0.74, 1.01) | 0.079 | .058 |

*Survey-weighted logistic regression estimates of factors associated with Polio vaccine coverage, NIS-Child 2013*.

## **Polio Vaccine Results 2014**

| Predictor | Odds Ratio | 95% CI | SE | P value |
| --- | --- | --- | --- | --- |
| (Intercept) | 25.51 | (2.64, 246.93) | 1.158 | .005 |
| Age Group | 1.28 | (1.08, 1.52) | 0.087 | .004 |
| Household size | 0.89 | (0.80, 0.99) | 0.055 | .036 |
| Breastfeeding History | 0.88 | (0.63, 1.23) | 0.169 | .454 |
| WIC benefits | 0.79 | (0.54, 1.16) | 0.193 | .231 |
| Maternal Education | 1.25 | (1.06, 1.47) | 0.085 | .009 |
| Firstborn | 1.15 | (0.82, 1.62) | 0.175 | .427 |
| Hispanic Ethnicity | 0.63 | (0.39, 1.02) | 0.246 | .059 |
| Income | 1.14 | (0.90, 1.43) | 0.118 | .280 |
| Language | 1.15 | (0.67, 1.98) | 0.274 | .602 |
| Maternal Age | 1.02 | (0.70, 1.47) | 0.189 | .929 |
| Marital Status | 1.14 | (0.80, 1.63) | 0.181 | .465 |
| Racial Identity | 1.05 | (0.89, 1.24) | 0.084 | .576 |
| Sex | 1.10 | (0.85, 1.42) | 0.132 | .481 |
| Insurance Status | 0.75 | (0.62, 0.91) | 0.098 | .003 |
| Region | 0.92 | (0.80, 1.06) | 0.070 | .242 |

*Survey-weighted logistic regression estimates of factors associated with Polio vaccine coverage, NIS-Child 2014*.

## **Polio Vaccine Results 2015**

| Predictor | Odds Ratio | 95% CI | SE | P value |
| --- | --- | --- | --- | --- |
| (Intercept) | 16.55 | (3.37, 81.25) | 0.812 | <.001 |
| Age Group | 1.10 | (0.95, 1.27) | 0.075 | .221 |
| Household size | 0.89 | (0.81, 0.98) | 0.050 | .023 |
| Breastfeeding History | 0.96 | (0.72, 1.29) | 0.149 | .800 |
| WIC benefits | 0.65 | (0.46, 0.93) | 0.181 | .018 |
| Maternal Education | 1.19 | (1.02, 1.38) | 0.077 | .023 |
| Firstborn | 1.39 | (1.04, 1.85) | 0.147 | .026 |
| Hispanic Ethnicity | 0.75 | (0.55, 1.02) | 0.157 | .064 |
| Income | 1.18 | (0.97, 1.43) | 0.100 | .102 |
| Language | 1.68 | (1.12, 2.52) | 0.208 | .013 |
| Maternal Age | 1.19 | (0.92, 1.54) | 0.132 | .185 |
| Marital Status | 0.76 | (0.57, 1.02) | 0.149 | .071 |
| Racial Identity | 1.06 | (0.90, 1.25) | 0.084 | .468 |
| Sex | 1.01 | (0.80, 1.27) | 0.119 | .948 |
| Insurance Status | 0.75 | (0.65, 0.87) | 0.075 | <.001 |
| Region | 1.00 | (0.89, 1.12) | 0.060 | .970 |

*Survey-weighted logistic regression estimates of factors associated with Polio vaccine coverage, NIS-Child 2015*.

## **Polio Vaccine Results 2016**

| Predictor | Odds Ratio | 95% CI | SE | P value |
| --- | --- | --- | --- | --- |
| (Intercept) | 37.80 | (6.15, 232.31) | 0.926 | <.001 |
| Age Group | 1.10 | (0.93, 1.29) | 0.084 | .275 |
| Household size | 0.91 | (0.82, 1.01) | 0.052 | .064 |
| Breastfeeding History | 0.96 | (0.68, 1.37) | 0.180 | .827 |
| WIC benefits | 0.56 | (0.34, 0.92) | 0.252 | .021 |
| Maternal Education | 1.22 | (1.00, 1.49) | 0.101 | .046 |
| Firstborn | 1.01 | (0.73, 1.38) | 0.161 | .968 |
| Hispanic Ethnicity | 0.91 | (0.62, 1.35) | 0.198 | .647 |
| Income | 1.24 | (0.95, 1.61) | 0.134 | .108 |
| Language | 1.39 | (0.87, 2.21) | 0.236 | .164 |
| Maternal Age | 0.96 | (0.69, 1.33) | 0.167 | .813 |
| Marital Status | 1.26 | (0.84, 1.88) | 0.205 | .262 |
| Racial Identity | 1.02 | (0.84, 1.24) | 0.100 | .846 |
| Sex | 0.99 | (0.74, 1.32) | 0.147 | .942 |
| Insurance Status | 0.71 | (0.59, 0.86) | 0.096 | <.001 |
| Region | 0.72 | (0.63, 0.84) | 0.074 | <.001 |

*Survey-weighted logistic regression estimates of factors associated with Polio vaccine coverage, NIS-Child 2016*.

## **Polio Vaccine Results 2017**

| Predictor | Odds Ratio | 95% CI | SE | P value |
| --- | --- | --- | --- | --- |
| (Intercept) | 18.14 | (1.97, 167.07) | 1.133 | .011 |
| Age Group | 1.11 | (0.94, 1.30) | 0.081 | .217 |
| Household size | 0.94 | (0.85, 1.04) | 0.050 | .210 |
| Breastfeeding History | 1.02 | (0.72, 1.44) | 0.176 | .907 |
| WIC benefits | 0.48 | (0.35, 0.65) | 0.158 | <.001 |
| Maternal Education | 1.15 | (0.99, 1.33) | 0.076 | .070 |
| Firstborn | 1.33 | (0.96, 1.85) | 0.167 | .089 |
| Hispanic Ethnicity | 0.90 | (0.61, 1.33) | 0.199 | .602 |
| Income | 1.32 | (1.11, 1.57) | 0.089 | .002 |
| Language | 1.36 | (0.92, 2.02) | 0.202 | .126 |
| Maternal Age | 1.54 | (1.17, 2.03) | 0.141 | .002 |
| Marital Status | 0.87 | (0.61, 1.24) | 0.180 | .431 |
| Racial Identity | 0.95 | (0.78, 1.14) | 0.096 | .558 |
| Sex | 0.91 | (0.71, 1.17) | 0.129 | .473 |
| Insurance Status | 0.73 | (0.64, 0.84) | 0.071 | <.001 |
| Region | 0.88 | (0.77, 1.01) | 0.070 | .080 |

*Survey-weighted logistic regression estimates of factors associated with Polio vaccine coverage, NIS-Child 2017*.

## **Polio Vaccine Results 2018**

| Predictor | Odds Ratio | 95% CI | SE | P value |
| --- | --- | --- | --- | --- |
| (Intercept) | 22.36 | (2.70, 185.02) | 1.078 | .004 |
| Age Group | 1.21 | (1.05, 1.40) | 0.074 | .010 |
| Household size | 0.88 | (0.77, 1.00) | 0.066 | .050 |
| Breastfeeding History | 1.30 | (0.94, 1.81) | 0.169 | .118 |
| WIC benefits | 0.57 | (0.40, 0.82) | 0.185 | .002 |
| Maternal Education | 1.12 | (0.94, 1.34) | 0.090 | .203 |
| Firstborn | 1.26 | (0.93, 1.70) | 0.154 | .134 |
| Hispanic Ethnicity | 0.98 | (0.69, 1.40) | 0.182 | .921 |
| Income | 1.24 | (1.02, 1.52) | 0.103 | .033 |
| Language | 1.76 | (1.17, 2.65) | 0.208 | .007 |
| Maternal Age | 1.07 | (0.78, 1.46) | 0.162 | .692 |
| Marital Status | 0.79 | (0.57, 1.10) | 0.171 | .167 |
| Racial Identity | 0.90 | (0.76, 1.05) | 0.083 | .189 |
| Sex | 1.13 | (0.88, 1.45) | 0.128 | .351 |
| Insurance Status | 0.64 | (0.56, 0.73) | 0.068 | <.001 |
| Region | 0.92 | (0.79, 1.06) | 0.076 | .250 |

*Survey-weighted logistic regression estimates of factors associated with Polio vaccine coverage, NIS-Child 2018*.

## **Polio Vaccine Results 2019**

| Predictor | OR | 95% CI | SE | p |
| --- | --- | --- | --- | --- |
| (Intercept) | 3.11 | (0.47, 20.64) | 0.965 | .239 |
| Age Group | 1.13 | (0.94, 1.35) | 0.092 | .199 |
| Household size | 0.97 | (0.85, 1.10) | 0.066 | .631 |
| Breastfeeding History | 1.63 | (1.13, 2.34) | 0.186 | .009 |
| WIC benefits | 0.73 | (0.47, 1.12) | 0.223 | .150 |
| Maternal Education | 1.42 | (1.15, 1.75) | 0.107 | .001 |
| Firstborn | 1.52 | (1.11, 2.09) | 0.161 | .009 |
| Hispanic Ethnicity | 0.88 | (0.62, 1.24) | 0.176 | .466 |
| Income | 1.17 | (0.94, 1.46) | 0.112 | .152 |
| Language | 1.54 | (1.02, 2.32) | 0.209 | .038 |
| Maternal Age | 1.28 | (0.95, 1.71) | 0.150 | .104 |
| Marital Status | 0.80 | (0.54, 1.18) | 0.199 | .267 |
| Racial Identity | 0.77 | (0.64, 0.93) | 0.098 | .008 |
| Sex | 0.86 | (0.65, 1.13) | 0.140 | .270 |
| Insurance Status | 0.82 | (0.67, 1.02) | 0.108 | .073 |
| Region | 1.02 | (0.87, 1.20) | 0.081 | .779 |

*Survey-weighted logistic regression estimates of factors associated with Polio vaccine coverage, NIS-Child 2019*.

## **Polio Vaccine Results 2020**

| Predictor | Odds Ratio | 95% CI | SE | P value |
| --- | --- | --- | --- | --- |
| (Intercept) | 3.80 | (0.54, 26.64) | 0.994 | .179 |
| Age Group | 1.14 | (0.98, 1.33) | 0.079 | .094 |
| Household size | 0.95 | (0.86, 1.05) | 0.051 | .280 |
| Breastfeeding History | 1.18 | (0.84, 1.64) | 0.170 | .342 |
| WIC benefits | 0.63 | (0.45, 0.89) | 0.174 | .008 |
| Maternal Education | 1.16 | (0.99, 1.36) | 0.082 | .075 |
| Firstborn | 1.46 | (1.06, 2.03) | 0.166 | .022 |
| Hispanic Ethnicity | 0.96 | (0.69, 1.33) | 0.168 | .792 |
| Income | 1.49 | (1.22, 1.81) | 0.100 | <.001 |
| Language | 1.20 | (0.79, 1.83) | 0.213 | .385 |
| Maternal Age | 1.51 | (1.16, 1.98) | 0.137 | .002 |
| Marital Status | 0.93 | (0.68, 1.27) | 0.158 | .656 |
| Racial Identity | 0.87 | (0.76, 1.00) | 0.071 | .046 |
| Sex | 1.29 | (1.02, 1.63) | 0.121 | .037 |
| Insurance Status | 0.80 | (0.69, 0.92) | 0.075 | .003 |
| Region | 0.97 | (0.86, 1.09) | 0.061 | .571 |

*Survey-weighted logistic regression estimates of factors associated with Polio vaccine coverage, NIS-Child 2020*.

## **Polio Vaccine Results 2021**

| Predictor | Odds Ratio | 95% CI | SE | P value |
| --- | --- | --- | --- | --- |
| (Intercept) | 13.46 | (1.53, 118.39) | 1.109 | .019 |
| Age Group | 1.14 | (0.97, 1.34) | 0.082 | .104 |
| Household size | 0.96 | (0.86, 1.07) | 0.056 | .507 |
| Breastfeeding History | 0.96 | (0.64, 1.43) | 0.205 | .838 |
| WIC benefits | 0.63 | (0.45, 0.87) | 0.165 | .005 |
| Maternal Education | 1.08 | (0.92, 1.27) | 0.082 | .327 |
| Firstborn | 1.40 | (1.01, 1.94) | 0.167 | .043 |
| Hispanic Ethnicity | 0.87 | (0.59, 1.28) | 0.198 | .474 |
| Income | 1.48 | (1.22, 1.78) | 0.096 | <.001 |
| Language | 1.21 | (0.59, 2.51) | 0.371 | .604 |
| Maternal Age | 1.52 | (1.12, 2.06) | 0.155 | .007 |
| Marital Status | 0.81 | (0.59, 1.11) | 0.161 | .187 |
| Racial Identity | 0.89 | (0.77, 1.04) | 0.077 | .147 |
| Sex | 1.09 | (0.83, 1.42) | 0.138 | .548 |
| Insurance Status | 0.65 | (0.51, 0.84) | 0.128 | <.001 |
| Region | 0.95 | (0.83, 1.10) | 0.071 | .518 |

*Survey-weighted logistic regression estimates of factors associated with Polio vaccine coverage, NIS-Child 2021.*

## **Polio Vaccine Results 2022**

| Predictor | Odds Ratio | 95% CI | SE | P value |
| --- | --- | --- | --- | --- |
| (Intercept) | 73.65 | (7.76, 698.83) | 1.148 | <.001 |
| Age Group | 1.03 | (0.87, 1.21) | 0.083 | .742 |
| Household size | 0.85 | (0.76, 0.94) | 0.053 | .002 |
| Breastfeeding History | 1.11 | (0.73, 1.68) | 0.212 | .622 |
| WIC benefits | 0.84 | (0.57, 1.23) | 0.195 | .369 |
| Maternal Education | 1.24 | (1.05, 1.46) | 0.084 | .011 |
| Firstborn | 1.07 | (0.77, 1.50) | 0.172 | .681 |
| Hispanic Ethnicity | 0.71 | (0.50, 1.03) | 0.186 | .070 |
| Income | 1.05 | (0.79, 1.40) | 0.147 | .754 |
| Language | 0.91 | (0.59, 1.41) | 0.225 | .673 |
| Maternal Age | 1.08 | (0.79, 1.47) | 0.159 | .645 |
| Marital Status | 0.95 | (0.66, 1.37) | 0.187 | .770 |
| Racial Identity | 0.84 | (0.71, 0.99) | 0.083 | .034 |
| Sex | 1.11 | (0.85, 1.45) | 0.136 | .441 |
| Insurance Status | 0.69 | (0.56, 0.85) | 0.105 | <.001 |
| Region | 0.96 | (0.84, 1.09) | 0.066 | .508 |

*Survey-weighted logistic regression estimates of factors associated with Polio vaccine coverage, NIS-Child 2022*.

## **Polio Vaccine Results 2023**

| Predictor | Odds Ratio | 95% CI | SE | P value |
| --- | --- | --- | --- | --- |
| (Intercept) | 207.95 | (40.77, 1060.51) | 0.831 | <.001 |
| Age Group | 1.00 | (0.86, 1.17) | 0.078 | .972 |
| Household size | 0.87 | (0.77, 0.98) | 0.063 | .023 |
| Breastfeeding History | 0.63 | (0.45, 0.88) | 0.170 | .006 |
| WIC benefits | 0.57 | (0.43, 0.77) | 0.149 | <.001 |
| Maternal Education | 1.14 | (1.00, 1.31) | 0.070 | .056 |
| Firstborn | 1.00 | (0.74, 1.35) | 0.152 | .980 |
| Hispanic Ethnicity | 0.79 | (0.59, 1.07) | 0.151 | .129 |
| Income | 1.25 | (1.04, 1.52) | 0.098 | .021 |
| Language | 0.98 | (0.64, 1.49) | 0.214 | .925 |
| Maternal Age | 1.33 | (1.01, 1.75) | 0.140 | .042 |
| Marital Status | 0.83 | (0.64, 1.09) | 0.136 | .176 |
| Racial Identity | 1.02 | (0.88, 1.17) | 0.072 | .824 |
| Sex | 0.95 | (0.75, 1.21) | 0.122 | .686 |
| Insurance Status | 0.67 | (0.57, 0.79) | 0.083 | <.001 |
| Region | 0.80 | (0.70, 0.91) | 0.064 | <.001 |

*Survey-weighted logistic regression estimates of factors associated with Polio vaccine coverage, NIS-Child 2023*.

## **Rotavirus Vaccine Results 2010**

| Predictor | Odds Ratio | 95% CI | SE | P value |
| --- | --- | --- | --- | --- |
| (Intercept) | 7.54 | (2.68, 21.25) | 0.528 | <.001 |
| Age Group | 0.73 | (0.67, 0.79) | 0.039 | <.001 |
| Household size | 0.89 | (0.84, 0.93) | 0.026 | <.001 |
| Breastfeeding History | 0.98 | (0.85, 1.13) | 0.074 | .795 |
| WIC benefits | 0.83 | (0.68, 1.02) | 0.104 | .079 |
| Maternal Education | 1.07 | (0.99, 1.16) | 0.041 | .103 |
| Firstborn | 1.02 | (0.89, 1.17) | 0.071 | .805 |
| Hispanic Ethnicity | 0.66 | (0.55, 0.80) | 0.095 | <.001 |
| Income | 1.11 | (1.00, 1.23) | 0.051 | .044 |
| Language | 1.36 | (1.08, 1.72) | 0.119 | .009 |
| Maternal Age | 1.12 | (0.97, 1.29) | 0.071 | .115 |
| Marital Status | 1.03 | (0.86, 1.23) | 0.091 | .772 |
| Racial Identity | 0.98 | (0.89, 1.07) | 0.047 | .599 |
| Sex | 1.02 | (0.90, 1.15) | 0.062 | .747 |
| Insurance Status | 0.93 | (0.85, 1.03) | 0.050 | .170 |
| Region | 1.03 | (0.97, 1.10) | 0.034 | .330 |

*Survey-weighted logistic regression estimates of factors associated with Polio vaccine coverage, NIS-Child 2010*.

## **Rotavirus Vaccine Results 2011**

| Predictor | Odds Ratio | 95% CI | SE | P value |
| --- | --- | --- | --- | --- |
| (Intercept) | 11.65 | (4.34, 31.30) | 0.504 | <.001 |
| Age Group | 0.81 | (0.74, 0.87) | 0.041 | <.001 |
| Household size | 0.95 | (0.89, 1.00) | 0.028 | .046 |
| Breastfeeding History | 0.96 | (0.82, 1.13) | 0.081 | .644 |
| WIC benefits | 0.72 | (0.58, 0.89) | 0.110 | .002 |
| Maternal Education | 1.08 | (0.99, 1.17) | 0.044 | .101 |
| Firstborn | 1.24 | (1.06, 1.46) | 0.081 | .007 |
| Hispanic Ethnicity | 0.62 | (0.49, 0.77) | 0.113 | <.001 |
| Income | 1.17 | (1.04, 1.31) | 0.060 | .011 |
| Language | 1.05 | (0.82, 1.34) | 0.124 | .693 |
| Maternal Age | 1.11 | (0.96, 1.27) | 0.070 | .147 |
| Marital Status | 1.05 | (0.87, 1.26) | 0.095 | .633 |
| Racial Identity | 0.99 | (0.90, 1.08) | 0.049 | .761 |
| Sex | 1.04 | (0.92, 1.19) | 0.066 | .514 |
| Insurance Status | 0.83 | (0.75, 0.91) | 0.049 | <.001 |
| Region | 1.03 | (0.96, 1.10) | 0.034 | .419 |

*Survey-weighted logistic regression estimates of factors associated with Rotavirus vaccine coverage, NIS-Child 2011*

## **Rotavirus Vaccine Results 2012**

| Predictor | Odds Ratio | 95% CI | SE | P value |
| --- | --- | --- | --- | --- |
| (Intercept) | 16.81 | (5.81, 48.63) | 0.542 | <.001 |
| Age Group | 0.91 | (0.83, 1.00) | 0.048 | .044 |
| Household size | 0.91 | (0.86, 0.97) | 0.033 | .006 |
| Breastfeeding History | 0.95 | (0.79, 1.13) | 0.089 | .533 |
| WIC benefits | 0.72 | (0.57, 0.90) | 0.114 | .004 |
| Maternal Education | 1.16 | (1.06, 1.27) | 0.047 | .002 |
| Firstborn | 1.07 | (0.88, 1.29) | 0.098 | .515 |
| Hispanic Ethnicity | 0.66 | (0.53, 0.83) | 0.113 | <.001 |
| Income | 1.12 | (0.99, 1.27) | 0.062 | .066 |
| Language | 1.24 | (0.98, 1.57) | 0.120 | .070 |
| Maternal Age | 1.01 | (0.86, 1.18) | 0.081 | .920 |
| Marital Status | 0.83 | (0.69, 1.01) | 0.097 | .058 |
| Racial Identity | 0.93 | (0.83, 1.03) | 0.054 | .167 |
| Sex | 0.96 | (0.83, 1.11) | 0.074 | .582 |
| Insurance Status | 0.86 | (0.79, 0.94) | 0.046 | .001 |
| Region | 1.02 | (0.95, 1.11) | 0.041 | .558 |

*Survey-weighted logistic regression estimates of factors associated with Rotavirus vaccine coverage, NIS-Child 2012*.

## **Rotavirus Vaccine Results 2013**

| Predictor | Odds Ratio | 95% CI | SE | P value |
| --- | --- | --- | --- | --- |
| (Intercept) | 4.45 | (1.28, 15.49) | 0.636 | .019 |
| Age Group | 0.97 | (0.88, 1.08) | 0.053 | .600 |
| Household size | 0.91 | (0.84, 0.98) | 0.039 | .019 |
| Breastfeeding History | 1.07 | (0.86, 1.33) | 0.112 | .562 |
| WIC benefits | 0.74 | (0.56, 0.97) | 0.139 | .031 |
| Maternal Education | 1.20 | (1.07, 1.34) | 0.057 | .001 |
| Firstborn | 1.22 | (0.98, 1.52) | 0.112 | .070 |
| Hispanic Ethnicity | 0.63 | (0.48, 0.83) | 0.142 | .001 |
| Income | 1.20 | (1.04, 1.39) | 0.074 | .014 |
| Language | 1.22 | (0.90, 1.66) | 0.156 | .200 |
| Maternal Age | 1.26 | (1.03, 1.56) | 0.107 | .028 |
| Marital Status | 1.05 | (0.83, 1.33) | 0.120 | .674 |
| Racial Identity | 0.92 | (0.82, 1.04) | 0.061 | .167 |
| Sex | 1.20 | (1.01, 1.42) | 0.087 | .038 |
| Insurance Status | 0.77 | (0.69, 0.87) | 0.062 | <.001 |
| Region | 1.00 | (0.92, 1.09) | 0.044 | .930 |

*Survey-weighted logistic regression estimates of factors associated with Rotavirus vaccine coverage, NIS-Child 2013*.

## **Rotavirus Vaccine Results 2014**

| Predictor | Odds Ratio | 95% CI | SE | P value |
| --- | --- | --- | --- | --- |
| (Intercept) | 20.25 | (4.79, 85.60) | 0.735 | <.001 |
| Age Group | 0.90 | (0.80, 1.01) | 0.058 | .066 |
| Household size | 0.85 | (0.79, 0.92) | 0.039 | <.001 |
| Breastfeeding History | 0.92 | (0.73, 1.15) | 0.116 | .457 |
| WIC benefits | 0.76 | (0.56, 1.04) | 0.159 | .087 |
| Maternal Education | 1.08 | (0.96, 1.21) | 0.059 | .213 |
| Firstborn | 0.88 | (0.71, 1.11) | 0.114 | .281 |
| Hispanic Ethnicity | 0.71 | (0.53, 0.96) | 0.152 | .025 |
| Income | 1.33 | (1.14, 1.56) | 0.080 | <.001 |
| Language | 1.29 | (0.94, 1.76) | 0.159 | .111 |
| Maternal Age | 1.06 | (0.84, 1.33) | 0.117 | .630 |
| Marital Status | 1.01 | (0.79, 1.30) | 0.129 | .923 |
| Racial Identity | 0.84 | (0.74, 0.95) | 0.062 | .005 |
| Sex | 1.16 | (0.97, 1.39) | 0.093 | .109 |
| Insurance Status | 0.81 | (0.71, 0.93) | 0.070 | .003 |
| Region | 1.01 | (0.92, 1.11) | 0.048 | .804 |

*Survey-weighted logistic regression estimates of factors associated with Rotavirus vaccine coverage, NIS-Child 2014*.

**Rotavirus Vaccine Results 2015**

| Predictor | Odds Ratio | 95% CI | SE | P value |
| --- | --- | --- | --- | --- |
| (Intercept) | 10.77 | (3.48, 33.32) | 0.576 | <.001 |
| Age Group | 0.94 | (0.84, 1.05) | 0.055 | .249 |
| Household size | 0.92 | (0.85, 0.98) | 0.037 | .016 |
| Breastfeeding History | 0.75 | (0.59, 0.95) | 0.122 | .018 |
| WIC benefits | 0.61 | (0.45, 0.82) | 0.151 | .001 |
| Maternal Education | 1.08 | (0.97, 1.21) | 0.057 | .160 |
| Firstborn | 1.20 | (0.98, 1.47) | 0.103 | .074 |
| Hispanic Ethnicity | 0.74 | (0.57, 0.96) | 0.135 | .025 |
| Income | 1.32 | (1.12, 1.55) | 0.083 | <.001 |
| Language | 1.20 | (0.94, 1.54) | 0.126 | .143 |
| Maternal Age | 1.42 | (1.18, 1.71) | 0.094 | <.001 |
| Marital Status | 1.01 | (0.79, 1.29) | 0.124 | .912 |
| Racial Identity | 0.97 | (0.85, 1.09) | 0.063 | .593 |
| Sex | 0.92 | (0.78, 1.10) | 0.088 | .371 |
| Insurance Status | 0.77 | (0.69, 0.86) | 0.054 | <.001 |
| Region | 0.98 | (0.89, 1.08) | 0.049 | .723 |

*Survey-weighted logistic regression estimates of factors associated with Rotavirus vaccine coverage, NIS-Child 2015*.

## **Rotavirus Vaccine Results 2016**

| Predictor | Odds Ratio | 95% CI | SE | P value |
| --- | --- | --- | --- | --- |
| (Intercept) | 13.97 | (3.17, 61.56) | 0.757 | <.001 |
| Age Group | 0.93 | (0.83, 1.04) | 0.056 | .208 |
| Household size | 0.91 | (0.84, 0.99) | 0.042 | .028 |
| Breastfeeding History | 0.88 | (0.70, 1.12) | 0.120 | .304 |
| WIC benefits | 0.78 | (0.55, 1.12) | 0.181 | .176 |
| Maternal Education | 1.16 | (1.03, 1.32) | 0.064 | .019 |
| Firstborn | 1.03 | (0.81, 1.30) | 0.120 | .827 |
| Hispanic Ethnicity | 0.74 | (0.56, 0.97) | 0.143 | .031 |
| Income | 1.20 | (0.99, 1.45) | 0.097 | .066 |
| Language | 0.97 | (0.73, 1.28) | 0.145 | .814 |
| Maternal Age | 1.27 | (1.00, 1.62) | 0.123 | .052 |
| Marital Status | 1.25 | (0.97, 1.61) | 0.129 | .086 |
| Racial Identity | 0.94 | (0.83, 1.07) | 0.065 | .364 |
| Sex | 0.93 | (0.77, 1.12) | 0.098 | .447 |
| Insurance Status | 0.82 | (0.69, 0.98) | 0.089 | .026 |
| Region | 0.91 | (0.82, 1.00) | 0.051 | .049 |

*Survey-weighted logistic regression estimates of factors associated with Rotavirus vaccine coverage, NIS-Child 2016*.

## **Rotavirus Vaccine Results 2017**

| Predictor | Odds Ratio | 95% CI | SE | P value |
| --- | --- | --- | --- | --- |
| (Intercept) | 22.61 | (5.27, 97.01) | 0.743 | <.001 |
| Age Group | 0.88 | (0.79, 0.98) | 0.055 | .024 |
| Household size | 0.90 | (0.84, 0.97) | 0.038 | .008 |
| Breastfeeding History | 0.78 | (0.61, 1.01) | 0.130 | .059 |
| WIC benefits | 0.52 | (0.39, 0.70) | 0.149 | <.001 |
| Maternal Education | 1.23 | (1.10, 1.38) | 0.060 | <.001 |
| Firstborn | 0.96 | (0.77, 1.21) | 0.117 | .756 |
| Hispanic Ethnicity | 0.72 | (0.52, 0.99) | 0.165 | .044 |
| Income | 1.55 | (1.34, 1.79) | 0.074 | <.001 |
| Language | 1.19 | (0.94, 1.52) | 0.123 | .153 |
| Maternal Age | 0.98 | (0.80, 1.21) | 0.104 | .878 |
| Marital Status | 1.26 | (0.99, 1.61) | 0.125 | .065 |
| Racial Identity | 0.92 | (0.81, 1.05) | 0.064 | .214 |
| Sex | 0.99 | (0.81, 1.19) | 0.098 | .885 |
| Insurance Status | 0.82 | (0.72, 0.93) | 0.063 | .002 |
| Region | 0.93 | (0.84, 1.04) | 0.054 | .198 |

*Survey-weighted logistic regression estimates of factors associated with Rotavirus vaccine coverage, NIS-Child 2017*.

**Rotavirus Vaccine Results 2018**

| Predictor | Odds Ratio | 95% CI | SE | P value |
| --- | --- | --- | --- | --- |
| (Intercept) | 6.00 | (1.14, 31.55) | 0.847 | .034 |
| Age Group | 1.10 | (0.97, 1.25) | 0.065 | .145 |
| Household size | 0.80 | (0.73, 0.88) | 0.047 | <.001 |
| Breastfeeding History | 1.10 | (0.82, 1.48) | 0.149 | .512 |
| WIC benefits | 0.86 | (0.61, 1.21) | 0.174 | .377 |
| Maternal Education | 1.24 | (1.06, 1.44) | 0.078 | .006 |
| Firstborn | 0.95 | (0.72, 1.25) | 0.140 | .714 |
| Hispanic Ethnicity | 0.89 | (0.65, 1.22) | 0.160 | .480 |
| Income | 1.36 | (1.15, 1.61) | 0.085 | <.001 |
| Language | 1.16 | (0.84, 1.61) | 0.167 | .375 |
| Maternal Age | 1.14 | (0.89, 1.47) | 0.128 | .303 |
| Marital Status | 1.22 | (0.91, 1.64) | 0.151 | .185 |
| Racial Identity | 0.91 | (0.79, 1.06) | 0.073 | .223 |
| Sex | 1.02 | (0.82, 1.26) | 0.108 | .858 |
| Insurance Status | 0.84 | (0.74, 0.95) | 0.066 | .008 |
| Region | 0.89 | (0.79, 1.00) | 0.059 | .050 |

*Survey-weighted logistic regression estimates of factors associated with Rotavirus vaccine coverage, NIS-Child 2018*.

## **Rotavirus Vaccine Results 2019**

| Predictor | Odds Ratio | 95% CI | SE | P value |
| --- | --- | --- | --- | --- |
| (Intercept) | 11.16 | (2.26, 55.09) | 0.815 | .003 |
| Age Group | 0.91 | (0.80, 1.04) | 0.065 | .165 |
| Household size | 0.90 | (0.83, 0.98) | 0.042 | .012 |
| Breastfeeding History | 1.08 | (0.80, 1.47) | 0.155 | .607 |
| WIC benefits | 0.67 | (0.46, 0.96) | 0.187 | .031 |
| Maternal Education | 1.10 | (0.96, 1.27) | 0.072 | .167 |
| Firstborn | 1.39 | (1.10, 1.75) | 0.118 | .005 |
| Hispanic Ethnicity | 0.81 | (0.60, 1.09) | 0.154 | .168 |
| Income | 1.26 | (1.06, 1.48) | 0.084 | .007 |
| Language | 1.52 | (1.10, 2.09) | 0.163 | .011 |
| Maternal Age | 1.39 | (1.11, 1.74) | 0.115 | .004 |
| Marital Status | 0.69 | (0.51, 0.93) | 0.155 | .016 |
| Racial Identity | 0.82 | (0.71, 0.95) | 0.073 | .008 |
| Sex | 1.00 | (0.81, 1.24) | 0.108 | .968 |
| Insurance Status | 0.78 | (0.69, 0.88) | 0.061 | <.001 |
| Region | 1.01 | (0.89, 1.14) | 0.063 | .881 |

*Survey-weighted logistic regression estimates of factors associated with Rotavirus vaccine coverage, NIS-Child 2019*.

## **Rotavirus Vaccine Results 2020**

| Predictor | Odds Ratio | 95% CI | SE | P value |
| --- | --- | --- | --- | --- |
| (Intercept) | 6.73 | (1.64, 27.55) | 0.719 | .008 |
| Age Group | 0.92 | (0.83, 1.03) | 0.055 | .156 |
| Household size | 0.93 | (0.86, 1.01) | 0.039 | .080 |
| Breastfeeding History | 1.03 | (0.81, 1.30) | 0.122 | .828 |
| WIC benefits | 0.70 | (0.53, 0.93) | 0.144 | .012 |
| Maternal Education | 1.18 | (1.05, 1.33) | 0.061 | .006 |
| Firstborn | 1.37 | (1.08, 1.73) | 0.119 | .008 |
| Hispanic Ethnicity | 0.96 | (0.74, 1.25) | 0.133 | .778 |
| Income | 1.38 | (1.20, 1.60) | 0.073 | <.001 |
| Language | 1.38 | (0.97, 1.97) | 0.181 | .074 |
| Maternal Age | 1.03 | (0.83, 1.28) | 0.109 | .783 |
| Marital Status | 0.92 | (0.74, 1.14) | 0.109 | .446 |
| Racial Identity | 0.87 | (0.78, 0.97) | 0.057 | .014 |
| Sex | 1.03 | (0.86, 1.22) | 0.091 | .781 |
| Insurance Status | 0.76 | (0.66, 0.87) | 0.068 | <.001 |
| Region | 1.02 | (0.93, 1.11) | 0.044 | .732 |

*Survey-weighted logistic regression estimates of factors associated with Rotavirus vaccine coverage, NIS-Child 2020*.

**Rotavirus Vaccine Results 2021**

| Predictor | Odds Ratio | 95% CI | SE | P value |
| --- | --- | --- | --- | --- |
| (Intercept) | 9.46 | (2.17, 41.22) | 0.751 | .003 |
| Age Group | 1.13 | (1.00, 1.27) | 0.062 | .048 |
| Household size | 0.97 | (0.89, 1.06) | 0.043 | .513 |
| Breastfeeding History | 0.99 | (0.76, 1.30) | 0.138 | .945 |
| WIC benefits | 0.81 | (0.62, 1.06) | 0.138 | .131 |
| Maternal Education | 1.17 | (1.03, 1.34) | 0.067 | .018 |
| Firstborn | 1.16 | (0.91, 1.47) | 0.123 | .237 |
| Hispanic Ethnicity | 0.63 | (0.48, 0.84) | 0.144 | .001 |
| Income | 1.32 | (1.14, 1.53) | 0.075 | <.001 |
| Language | 1.29 | (0.79, 2.10) | 0.250 | .310 |
| Maternal Age | 1.23 | (0.98, 1.55) | 0.116 | .070 |
| Marital Status | 0.81 | (0.61, 1.07) | 0.142 | .140 |
| Racial Identity | 0.97 | (0.86, 1.10) | 0.063 | .658 |
| Sex | 1.07 | (0.88, 1.30) | 0.102 | .516 |
| Insurance Status | 0.75 | (0.62, 0.90) | 0.093 | .002 |
| Region | 0.93 | (0.84, 1.03) | 0.054 | .178 |

*Survey-weighted logistic regression estimates of factors associated with Rotavirus vaccine coverage, NIS-Child 2021*.

## **Rotavirus Vaccine Results 2022**

| Predictor | Odds Ratio | 95% CI | SE | P value |
| --- | --- | --- | --- | --- |
| (Intercept) | 19.31 | (4.13, 90.27) | 0.787 | <.001 |
| Age Group | 0.92 | (0.82, 1.04) | 0.059 | .179 |
| Household size | 0.90 | (0.83, 0.97) | 0.042 | .009 |
| Breastfeeding History | 1.04 | (0.77, 1.39) | 0.149 | .815 |
| WIC benefits | 0.85 | (0.64, 1.11) | 0.141 | .232 |
| Maternal Education | 1.23 | (1.10, 1.38) | 0.059 | <.001 |
| Firstborn | 1.11 | (0.85, 1.44) | 0.133 | .444 |
| Hispanic Ethnicity | 0.68 | (0.52, 0.89) | 0.137 | .005 |
| Income | 1.24 | (1.04, 1.49) | 0.093 | .020 |
| Language | 1.10 | (0.81, 1.49) | 0.154 | .538 |
| Maternal Age | 1.17 | (0.93, 1.48) | 0.119 | .180 |
| Marital Status | 0.82 | (0.63, 1.07) | 0.135 | .153 |
| Racial Identity | 1.02 | (0.90, 1.14) | 0.060 | .793 |
| Sex | 0.98 | (0.81, 1.19) | 0.098 | .827 |
| Insurance Status | 0.69 | (0.60, 0.79) | 0.071 | <.001 |
| Region | 0.98 | (0.90, 1.08) | 0.047 | .744 |

*Survey-weighted logistic regression estimates of factors associated with Rotavirus vaccine coverage, NIS-Child 2022*.

## **Rotavirus Vaccine Results 2023**

| Predictor | Odds Ratio | 95% CI | SE | P value |
| --- | --- | --- | --- | --- |
| (Intercept) | 38.49 | (10.53, 140.74) | 0.661 | <.001 |
| Age Group | 0.81 | (0.72, 0.91) | 0.060 | <.001 |
| Household size | 0.87 | (0.79, 0.95) | 0.045 | .002 |
| Breastfeeding History | 0.75 | (0.58, 0.97) | 0.132 | .028 |
| WIC benefits | 0.77 | (0.61, 0.97) | 0.119 | .029 |
| Maternal Education | 1.28 | (1.15, 1.43) | 0.055 | <.001 |
| Firstborn | 1.16 | (0.91, 1.48) | 0.123 | .221 |
| Hispanic Ethnicity | 0.81 | (0.63, 1.03) | 0.126 | .091 |
| Income | 1.21 | (1.05, 1.41) | 0.076 | .011 |
| Language | 1.03 | (0.78, 1.35) | 0.142 | .860 |
| Maternal Age | 1.19 | (0.96, 1.48) | 0.110 | .117 |
| Marital Status | 1.00 | (0.81, 1.24) | 0.107 | .972 |
| Racial Identity | 0.98 | (0.87, 1.10) | 0.058 | .725 |
| Sex | 1.04 | (0.87, 1.26) | 0.095 | .650 |
| Insurance Status | 0.65 | (0.57, 0.74) | 0.068 | <.001 |
| Region | 0.84 | (0.76, 0.92) | 0.048 | <.001 |

*Survey-weighted logistic regression estimates of factors associated with Rotavirus vaccine coverage, NIS-Child 2023*.

## **7 VACCINE SERIES Results 2010**

| Predictor | Odds Ratio | 95% CI | SE | P value |
| --- | --- | --- | --- | --- |
| (Intercept) | 4.35 | (1.56, 12.12) | 0.522 | .005 |
| Age Group | 1.10 | (1.02, 1.19) | 0.040 | .015 |
| Household size | 0.89 | (0.84, 0.94) | 0.027 | <.001 |
| Breastfeeding History | 0.95 | (0.82, 1.11) | 0.076 | .521 |
| WIC benefits | 0.69 | (0.56, 0.87) | 0.113 | .001 |
| Maternal Education | 1.04 | (0.96, 1.13) | 0.040 | .312 |
| Firstborn | 1.07 | (0.93, 1.22) | 0.071 | .368 |
| Hispanic Ethnicity | 0.90 | (0.74, 1.09) | 0.099 | .293 |
| Income | 1.13 | (1.02, 1.26) | 0.054 | .022 |
| Language | 1.50 | (1.18, 1.91) | 0.123 | .001 |
| Maternal Age | 1.03 | (0.89, 1.18) | 0.071 | .707 |
| Marital Status | 1.00 | (0.84, 1.19) | 0.089 | .984 |
| Racial Identity | 0.96 | (0.88, 1.06) | 0.046 | .431 |
| Sex | 1.07 | (0.95, 1.22) | 0.064 | .272 |
| Insurance Status | 0.81 | (0.73, 0.89) | 0.051 | <.001 |
| Region | 0.97 | (0.90, 1.04) | 0.035 | .336 |

*Survey-weighted logistic regression estimates of factors associated with 7 vaccine series coverage, NIS-Child 2010*.

## **7 VACCINE SERIES Results 2011**

| Predictor | Odds Ratio | 95% CI | SE | P value |
| --- | --- | --- | --- | --- |
| (Intercept) | 1.97 | (0.75, 5.20) | 0.495 | .171 |
| Age Group | 1.29 | (1.20, 1.39) | 0.038 | <.001 |
| Household size | 0.96 | (0.91, 1.02) | 0.028 | .183 |
| Breastfeeding History | 0.88 | (0.75, 1.03) | 0.082 | .109 |
| WIC benefits | 0.67 | (0.54, 0.83) | 0.107 | <.001 |
| Maternal Education | 0.98 | (0.90, 1.06) | 0.042 | .629 |
| Firstborn | 1.33 | (1.14, 1.55) | 0.078 | <.001 |
| Hispanic Ethnicity | 1.00 | (0.83, 1.21) | 0.097 | .960 |
| Income | 1.26 | (1.13, 1.41) | 0.057 | <.001 |
| Language | 1.35 | (1.09, 1.67) | 0.108 | .005 |
| Maternal Age | 1.05 | (0.91, 1.20) | 0.071 | .530 |
| Marital Status | 0.96 | (0.81, 1.14) | 0.087 | .624 |
| Racial Identity | 0.98 | (0.89, 1.08) | 0.049 | .692 |
| Sex | 0.97 | (0.85, 1.09) | 0.064 | .588 |
| Insurance Status | 0.80 | (0.73, 0.89) | 0.050 | <.001 |
| Region | 1.03 | (0.97, 1.10) | 0.033 | .312 |

*Survey-weighted logistic regression estimates of factors associated with 7 Vaccine Series, NIS-Child 2011*.

## **7 VACCINE SERIES Results 2012**

| Predictor | Odds Ratio | 95% CI | SE | P value |
| --- | --- | --- | --- | --- |
| (Intercept) | 2.63 | (0.97, 7.11) | 0.508 | .057 |
| Age Group | 1.22 | (1.12, 1.33) | 0.044 | <.001 |
| Household size | 0.88 | (0.83, 0.93) | 0.030 | <.001 |
| Breastfeeding History | 1.12 | (0.95, 1.33) | 0.084 | .163 |
| WIC benefits | 0.72 | (0.58, 0.89) | 0.107 | .002 |
| Maternal Education | 1.12 | (1.03, 1.22) | 0.044 | .008 |
| Firstborn | 1.14 | (0.95, 1.38) | 0.095 | .158 |
| Hispanic Ethnicity | 0.86 | (0.69, 1.06) | 0.109 | .158 |
| Income | 1.13 | (1.01, 1.26) | 0.059 | .040 |
| Language | 1.17 | (0.94, 1.45) | 0.111 | .171 |
| Maternal Age | 1.15 | (0.99, 1.33) | 0.075 | .064 |
| Marital Status | 0.90 | (0.75, 1.08) | 0.091 | .245 |
| Racial Identity | 1.03 | (0.94, 1.13) | 0.048 | .495 |
| Sex | 0.92 | (0.80, 1.06) | 0.070 | .251 |
| Insurance Status | 0.84 | (0.77, 0.91) | 0.045 | <.001 |
| Region | 1.01 | (0.94, 1.09) | 0.038 | .759 |

*Survey-weighted logistic regression estimates of factors associated with 7 Vaccine Series, NIS-Child 2012*.

## **7 VACCINE SERIES Results 2013**

| Predictor | Odds Ratio | 95% CI | SE | P value |
| --- | --- | --- | --- | --- |
| (Intercept) | 1.00 | (0.31, 3.20) | 0.592 | .996 |
| Age Group | 1.25 | (1.14, 1.37) | 0.047 | <.001 |
| Household size | 0.97 | (0.91, 1.04) | 0.034 | .389 |
| Breastfeeding History | 1.01 | (0.83, 1.22) | 0.097 | .950 |
| WIC benefits | 0.86 | (0.68, 1.09) | 0.122 | .207 |
| Maternal Education | 1.12 | (1.02, 1.24) | 0.050 | .020 |
| Firstborn | 1.44 | (1.19, 1.73) | 0.095 | <.001 |
| Hispanic Ethnicity | 0.80 | (0.63, 1.01) | 0.120 | .056 |
| Income | 1.16 | (1.02, 1.32) | 0.065 | .022 |
| Language | 1.16 | (0.90, 1.50) | 0.130 | .248 |
| Maternal Age | 1.22 | (1.03, 1.45) | 0.087 | .019 |
| Marital Status | 1.04 | (0.85, 1.27) | 0.101 | .690 |
| Racial Identity | 1.03 | (0.93, 1.14) | 0.053 | .560 |
| Sex | 1.05 | (0.90, 1.22) | 0.078 | .521 |
| Insurance Status | 0.75 | (0.68, 0.83) | 0.051 | <.001 |
| Region | 0.96 | (0.88, 1.03) | 0.039 | .245 |

*Survey-weighted logistic regression estimates of factors associated with 7 Vaccine Series, NIS-Child 2013*.

## **7 VACCINE SERIES Results 2014**

| Predictor | Odds Ratio | 95% CI | SE | P value |
| --- | --- | --- | --- | --- |
| (Intercept) | 1.89 | (0.57, 6.23) | 0.609 | .296 |
| Age Group | 1.23 | (1.12, 1.36) | 0.050 | <.001 |
| Household size | 0.92 | (0.86, 0.98) | 0.033 | .008 |
| Breastfeeding History | 1.00 | (0.82, 1.22) | 0.103 | .979 |
| WIC benefits | 0.84 | (0.66, 1.07) | 0.123 | .150 |
| Maternal Education | 1.10 | (1.00, 1.21) | 0.051 | .060 |
| Firstborn | 1.06 | (0.87, 1.28) | 0.098 | .569 |
| Hispanic Ethnicity | 0.70 | (0.55, 0.89) | 0.124 | .004 |
| Income | 1.26 | (1.11, 1.43) | 0.066 | <.001 |
| Language | 1.21 | (0.95, 1.55) | 0.126 | .123 |
| Maternal Age | 1.20 | (1.00, 1.44) | 0.094 | .056 |
| Marital Status | 1.18 | (0.95, 1.45) | 0.108 | .132 |
| Racial Identity | 0.96 | (0.86, 1.07) | 0.055 | .426 |
| Sex | 1.13 | (0.97, 1.33) | 0.080 | .121 |
| Insurance Status | 0.82 | (0.73, 0.92) | 0.058 | <.001 |
| Region | 0.98 | (0.91, 1.06) | 0.040 | .652 |

*Survey-weighted logistic regression estimates of factors associated with 7 Vaccine Series, NIS-Child 2014*.

## **7 VACCINE SERIES Results 2015**

| Predictor | Odds Ratio | 95% CI | SE | P value |
| --- | --- | --- | --- | --- |
| (Intercept) | 1.65 | (0.43, 6.33) | 0.685 | .463 |
| Age Group | 1.21 | (1.10, 1.33) | 0.048 | <.001 |
| Household size | 0.96 | (0.90, 1.02) | 0.032 | .149 |
| Breastfeeding History | 1.00 | (0.83, 1.20) | 0.093 | .997 |
| WIC benefits | 0.62 | (0.47, 0.80) | 0.135 | <.001 |
| Maternal Education | 1.08 | (0.99, 1.19) | 0.048 | .099 |
| Firstborn | 1.56 | (1.30, 1.87) | 0.093 | <.001 |
| Hispanic Ethnicity | 0.88 | (0.70, 1.10) | 0.115 | .264 |
| Income | 1.16 | (1.02, 1.32) | 0.066 | .025 |
| Language | 1.13 | (0.92, 1.40) | 0.107 | .246 |
| Maternal Age | 1.38 | (1.15, 1.64) | 0.089 | <.001 |
| Marital Status | 0.90 | (0.72, 1.12) | 0.112 | .357 |
| Racial Identity | 1.01 | (0.91, 1.12) | 0.051 | .850 |
| Sex | 1.03 | (0.89, 1.20) | 0.076 | .695 |
| Insurance Status | 0.76 | (0.69, 0.84) | 0.052 | <.001 |
| Region | 0.98 | (0.91, 1.07) | 0.041 | .683 |

*Survey-weighted logistic regression estimates of factors associated with 7 Vaccine Series, NIS-Child 2015*.

## **7 VACCINE SERIES Results 2016**

| Predictor | Odds Ratio | 95% CI | SE | P value |
| --- | --- | --- | --- | --- |
| (Intercept) | 1.71 | (0.51, 5.69) | 0.614 | .382 |
| Age Group | 1.20 | (1.09, 1.32) | 0.047 | <.001 |
| Household size | 0.94 | (0.88, 1.01) | 0.034 | .077 |
| Breastfeeding History | 1.08 | (0.87, 1.33) | 0.107 | .492 |
| WIC benefits | 0.92 | (0.68, 1.24) | 0.152 | .593 |
| Maternal Education | 1.12 | (1.01, 1.25) | 0.056 | .039 |
| Firstborn | 1.23 | (1.01, 1.50) | 0.099 | .035 |
| Hispanic Ethnicity | 0.92 | (0.72, 1.16) | 0.121 | .485 |
| Income | 1.09 | (0.94, 1.27) | 0.077 | .260 |
| Language | 1.20 | (0.93, 1.53) | 0.127 | .158 |
| Maternal Age | 1.16 | (0.96, 1.41) | 0.099 | .131 |
| Marital Status | 1.04 | (0.83, 1.30) | 0.114 | .723 |
| Racial Identity | 0.95 | (0.85, 1.07) | 0.060 | .406 |
| Sex | 1.02 | (0.87, 1.20) | 0.083 | .812 |
| Insurance Status | 0.82 | (0.71, 0.95) | 0.074 | .007 |
| Region | 0.89 | (0.82, 0.96) | 0.042 | .004 |

*Survey-weighted logistic regression estimates of factors associated with 7 Vaccine Series, NIS-Child 2016*

## **7 VACCINE SERIES Results 2017**

| Predictor | Odds Ratio | 95% CI | SE | P value |
| --- | --- | --- | --- | --- |
| (Intercept) | 3.11 | (1.03, 9.37) | 0.563 | .044 |
| Age Group | 1.23 | (1.12, 1.35) | 0.048 | <.001 |
| Household size | 0.95 | (0.88, 1.01) | 0.034 | .099 |
| Breastfeeding History | 0.94 | (0.75, 1.16) | 0.110 | .549 |
| WIC benefits | 0.62 | (0.48, 0.80) | 0.128 | <.001 |
| Maternal Education | 1.11 | (1.00, 1.22) | 0.050 | .043 |
| Firstborn | 1.27 | (1.05, 1.54) | 0.099 | .016 |
| Hispanic Ethnicity | 0.88 | (0.69, 1.10) | 0.118 | .258 |
| Income | 1.34 | (1.18, 1.52) | 0.065 | <.001 |
| Language | 1.09 | (0.89, 1.34) | 0.105 | .395 |
| Maternal Age | 1.23 | (1.03, 1.45) | 0.087 | .019 |
| Marital Status | 0.88 | (0.71, 1.10) | 0.112 | .255 |
| Racial Identity | 0.92 | (0.83, 1.02) | 0.053 | .095 |
| Sex | 1.00 | (0.86, 1.17) | 0.079 | .979 |
| Insurance Status | 0.83 | (0.75, 0.92) | 0.052 | <.001 |
| Region | 0.94 | (0.86, 1.03) | 0.044 | .171 |

*Survey-weighted logistic regression estimates of factors associated with 7 Vaccine Series, NIS-Child 2017*.

## **7 VACCINE SERIES Results 2018**

| Predictor | Odds Ratio | 95% CI | SE | P value |
| --- | --- | --- | --- | --- |
| (Intercept) | 1.61 | (0.50, 5.22) | 0.600 | .427 |
| Age Group | 1.38 | (1.24, 1.52) | 0.052 | <.001 |
| Household size | 0.89 | (0.82, 0.97) | 0.042 | .008 |
| Breastfeeding History | 0.95 | (0.76, 1.18) | 0.111 | .638 |
| WIC benefits | 0.99 | (0.74, 1.32) | 0.148 | .931 |
| Maternal Education | 1.13 | (1.00, 1.27) | 0.062 | .054 |
| Firstborn | 1.25 | (0.99, 1.58) | 0.119 | .062 |
| Hispanic Ethnicity | 1.00 | (0.77, 1.30) | 0.134 | .983 |
| Income | 1.11 | (0.96, 1.29) | 0.075 | .151 |
| Language | 1.50 | (1.15, 1.96) | 0.135 | .003 |
| Maternal Age | 1.11 | (0.92, 1.34) | 0.098 | .291 |
| Marital Status | 0.92 | (0.74, 1.16) | 0.115 | .484 |
| Racial Identity | 1.01 | (0.91, 1.13) | 0.056 | .839 |
| Sex | 0.98 | (0.83, 1.16) | 0.087 | .823 |
| Insurance Status | 0.72 | (0.65, 0.80) | 0.053 | <.001 |
| Region | 0.95 | (0.86, 1.04) | 0.049 | .250 |

*Survey-weighted logistic regression estimates of factors associated with 7 Vaccine Series, NIS-Child 2018*.

## **7 VACCINE SERIES Results 2019**

| Predictor | Odds Ratio | 95% CI | SE | P value |
| --- | --- | --- | --- | --- |
| (Intercept) | 2.96 | (0.84, 10.37) | 0.640 | .090 |
| Age Group | 1.15 | (1.03, 1.28) | 0.056 | .014 |
| Household size | 0.89 | (0.82, 0.97) | 0.041 | .005 |
| Breastfeeding History | 1.26 | (1.00, 1.58) | 0.116 | .050 |
| WIC benefits | 0.77 | (0.59, 1.02) | 0.140 | .066 |
| Maternal Education | 1.15 | (1.02, 1.29) | 0.060 | .022 |
| Firstborn | 1.33 | (1.06, 1.66) | 0.116 | .015 |
| Hispanic Ethnicity | 0.94 | (0.72, 1.22) | 0.134 | .623 |
| Income | 1.16 | (1.01, 1.33) | 0.071 | .039 |
| Language | 1.32 | (1.00, 1.75) | 0.144 | .050 |
| Maternal Age | 1.27 | (1.05, 1.55) | 0.099 | .016 |
| Marital Status | 0.84 | (0.66, 1.06) | 0.119 | .141 |
| Racial Identity | 0.80 | (0.70, 0.90) | 0.064 | <.001 |
| Sex | 0.97 | (0.81, 1.16) | 0.092 | .721 |
| Insurance Status | 0.82 | (0.73, 0.92) | 0.061 | .001 |
| Region | 0.93 | (0.84, 1.03) | 0.053 | .160 |

*Survey-weighted logistic regression estimates of factors associated with 7 Vaccine Series, NIS-Child 2019*.

## **7 VACCINE SERIES Results 2020**

| Predictor | Odds Ratio | 95% CI | SE | P value |
| --- | --- | --- | --- | --- |
| (Intercept) | 1.51 | (0.53, 4.34) | 0.538 | .443 |
| Age Group | 1.26 | (1.16, 1.38) | 0.044 | <.001 |
| Household size | 0.93 | (0.87, 0.99) | 0.032 | .021 |
| Breastfeeding History | 1.03 | (0.85, 1.24) | 0.098 | .794 |
| WIC benefits | 0.80 | (0.64, 0.99) | 0.112 | .042 |
| Maternal Education | 1.08 | (0.98, 1.18) | 0.048 | .110 |
| Firstborn | 1.29 | (1.08, 1.53) | 0.090 | .005 |
| Hispanic Ethnicity | 1.10 | (0.92, 1.33) | 0.095 | .294 |
| Income | 1.26 | (1.13, 1.41) | 0.057 | <.001 |
| Language | 1.15 | (0.90, 1.48) | 0.127 | .260 |
| Maternal Age | 1.29 | (1.09, 1.53) | 0.087 | .003 |
| Marital Status | 0.83 | (0.70, 0.99) | 0.089 | .037 |
| Racial Identity | 0.91 | (0.84, 0.99) | 0.044 | .033 |
| Sex | 0.95 | (0.83, 1.09) | 0.070 | .468 |
| Insurance Status | 0.83 | (0.75, 0.92) | 0.053 | <.001 |
| Region | 1.00 | (0.94, 1.07) | 0.034 | .971 |

*Survey-weighted logistic regression estimates of factors associated with 7 Vaccine Series, NIS-Child 2020*.

## **7 VACCINE SERIES Results 2021**

| Predictor | Odds Ratio | 95% CI | SE | P value |
| --- | --- | --- | --- | --- |
| (Intercept) | 2.91 | (0.93, 9.12) | 0.584 | .068 |
| Age Group | 1.36 | (1.23, 1.50) | 0.050 | <.001 |
| Household size | 0.94 | (0.88, 1.01) | 0.034 | .079 |
| Breastfeeding History | 1.04 | (0.83, 1.32) | 0.120 | .715 |
| WIC benefits | 0.79 | (0.64, 0.98) | 0.110 | .032 |
| Maternal Education | 1.15 | (1.04, 1.27) | 0.050 | .005 |
| Firstborn | 1.30 | (1.06, 1.58) | 0.101 | .010 |
| Hispanic Ethnicity | 0.85 | (0.69, 1.04) | 0.104 | .115 |
| Income | 1.20 | (1.06, 1.36) | 0.064 | .004 |
| Language | 1.15 | (0.84, 1.58) | 0.160 | .381 |
| Maternal Age | 1.26 | (1.06, 1.51) | 0.091 | .011 |
| Marital Status | 0.72 | (0.59, 0.88) | 0.101 | .001 |
| Racial Identity | 0.97 | (0.88, 1.07) | 0.050 | .550 |
| Sex | 1.02 | (0.87, 1.19) | 0.078 | .809 |
| Insurance Status | 0.70 | (0.62, 0.80) | 0.063 | <.001 |
| Region | 0.95 | (0.87, 1.03) | 0.041 | .188 |

*Survey-weighted logistic regression estimates of factors associated with 7 Vaccine Series, NIS-Child 2021.*

## **7 VACCINE SERIES Results 2022**

| Predictor | Odds Ratio | 95% CI | SE | P value |
| --- | --- | --- | --- | --- |
| (Intercept) | 3.91 | (1.20, 12.77) | 0.604 | .024 |
| Age Group | 1.27 | (1.16, 1.40) | 0.048 | <.001 |
| Household size | 0.88 | (0.82, 0.95) | 0.036 | <.001 |
| Breastfeeding History | 0.90 | (0.71, 1.12) | 0.115 | .341 |
| WIC benefits | 0.71 | (0.57, 0.88) | 0.111 | .002 |
| Maternal Education | 1.32 | (1.20, 1.46) | 0.051 | <.001 |
| Firstborn | 1.24 | (1.01, 1.51) | 0.102 | .037 |
| Hispanic Ethnicity | 0.89 | (0.72, 1.09) | 0.104 | .260 |
| Income | 1.18 | (1.03, 1.35) | 0.069 | .018 |
| Language | 1.17 | (0.90, 1.53) | 0.136 | .243 |
| Maternal Age | 1.22 | (1.01, 1.47) | 0.095 | .038 |
| Marital Status | 0.90 | (0.74, 1.10) | 0.103 | .319 |
| Racial Identity | 0.91 | (0.83, 1.01) | 0.050 | .070 |
| Sex | 0.93 | (0.80, 1.09) | 0.079 | .389 |
| Insurance Status | 0.74 | (0.66, 0.84) | 0.063 | <.001 |
| Region | 0.93 | (0.86, 1.00) | 0.039 | .053 |

*Survey-weighted logistic regression estimates of factors associated with 7 Vaccine Series, NIS-Child 2022*.

## **7 VACCINE SERIES Results 2023**

| Predictor | Odds Ratio | 95% CI | SE | P value |
| --- | --- | --- | --- | --- |
| (Intercept) | 6.79 | (2.39, 19.28) | 0.533 | <.001 |
| Age Group | 1.24 | (1.13, 1.36) | 0.047 | <.001 |
| Household size | 0.87 | (0.81, 0.93) | 0.036 | <.001 |
| Breastfeeding History | 0.78 | (0.63, 0.97) | 0.111 | .028 |
| WIC benefits | 0.81 | (0.67, 0.98) | 0.098 | .029 |
| Maternal Education | 1.19 | (1.08, 1.31) | 0.049 | <.001 |
| Firstborn | 1.06 | (0.88, 1.27) | 0.094 | .542 |
| Hispanic Ethnicity | 0.85 | (0.70, 1.04) | 0.098 | .110 |
| Income | 1.25 | (1.10, 1.41) | 0.062 | <.001 |
| Language | 1.01 | (0.79, 1.28) | 0.122 | .953 |
| Maternal Age | 1.14 | (0.95, 1.37) | 0.092 | .151 |
| Marital Status | 0.93 | (0.77, 1.12) | 0.094 | .452 |
| Racial Identity | 0.98 | (0.89, 1.07) | 0.046 | .635 |
| Sex | 1.01 | (0.87, 1.17) | 0.076 | .872 |
| Insurance Status | 0.76 | (0.68, 0.85) | 0.056 | <.001 |
| Region | 0.88 | (0.82, 0.95) | 0.039 | .001 |

*Survey-weighted logistic regression estimates of factors associated with 7 Vaccine Series, NIS-Child 2023*.

## **Varicella Results 2010**

| Predictor | Odds Ratio | 95% CI | SE | P value |
| --- | --- | --- | --- | --- |
| (Intercept) | 16.92 | (2.65, 107.99) | 0.946 | .003 |
| Age Group | 1.18 | (1.04, 1.34) | 0.065 | .009 |
| Household size | 0.83 | (0.76, 0.90) | 0.042 | <.001 |
| Breastfeeding History | 1.11 | (0.88, 1.39) | 0.116 | .381 |
| WIC benefits | 0.68 | (0.51, 0.91) | 0.148 | .009 |
| Maternal Education | 1.02 | (0.89, 1.17) | 0.069 | .801 |
| Firstborn | 1.01 | (0.80, 1.26) | 0.114 | .959 |
| Hispanic Ethnicity | 0.71 | (0.51, 0.99) | 0.172 | .044 |
| Income | 1.18 | (1.00, 1.39) | 0.084 | .054 |
| Language | 1.36 | (0.86, 2.14) | 0.231 | .186 |
| Maternal Age | 1.18 | (0.95, 1.45) | 0.108 | .134 |
| Marital Status | 1.26 | (0.96, 1.64) | 0.135 | .093 |
| Racial Identity | 1.05 | (0.90, 1.23) | 0.078 | .506 |
| Sex | 0.99 | (0.80, 1.22) | 0.106 | .934 |
| Insurance Status | 0.90 | (0.78, 1.04) | 0.076 | .167 |
| Region | 0.94 | (0.83, 1.06) | 0.061 | .305 |

*Survey-weighted logistic regression estimates of factors associated with Varicella Vaccine, NIS-Child 2010*.

## **Varicella Results 2011**

| Predictor | Odds Ratio | 95% CI | SE | P value |
| --- | --- | --- | --- | --- |
| (Intercept) | 13.34 | (3.14, 56.61) | 0.738 | <.001 |
| Age Group | 1.33 | (1.19, 1.49) | 0.057 | <.001 |
| Household size | 0.88 | (0.82, 0.95) | 0.038 | .001 |
| Breastfeeding History | 0.92 | (0.73, 1.16) | 0.120 | .482 |
| WIC benefits | 0.50 | (0.37, 0.68) | 0.155 | <.001 |
| Maternal Education | 1.02 | (0.91, 1.15) | 0.059 | .694 |
| Firstborn | 1.28 | (1.02, 1.61) | 0.117 | .036 |
| Hispanic Ethnicity | 0.77 | (0.58, 1.03) | 0.149 | .082 |
| Income | 1.21 | (1.04, 1.42) | 0.080 | .015 |
| Language | 1.61 | (1.13, 2.31) | 0.184 | .009 |
| Maternal Age | 1.11 | (0.93, 1.34) | 0.093 | .245 |
| Marital Status | 1.14 | (0.89, 1.47) | 0.130 | .301 |
| Racial Identity | 1.19 | (1.03, 1.37) | 0.073 | .019 |
| Sex | 1.04 | (0.87, 1.25) | 0.093 | .642 |
| Insurance Status | 0.73 | (0.65, 0.82) | 0.060 | <.001 |
| Region | 0.96 | (0.87, 1.06) | 0.050 | .403 |

*Survey-weighted logistic regression estimates of factors associated with Varicella Vaccine, NIS-Child 2011*.

## **Varicella Results 2012**

| Predictor | Odds Ratio | 95% CI | SE | P value |
| --- | --- | --- | --- | --- |
| (Intercept) | 37.59 | (8.78, 160.95) | 0.742 | <.001 |
| Age Group | 1.20 | (1.06, 1.37) | 0.066 | .006 |
| Household size | 0.88 | (0.81, 0.95) | 0.042 | .002 |
| Breastfeeding History | 1.20 | (0.93, 1.57) | 0.134 | .167 |
| WIC benefits | 0.57 | (0.42, 0.79) | 0.161 | <.001 |
| Maternal Education | 1.20 | (1.06, 1.37) | 0.064 | .004 |
| Firstborn | 1.05 | (0.82, 1.35) | 0.128 | .712 |
| Hispanic Ethnicity | 0.75 | (0.53, 1.05) | 0.177 | .097 |
| Income | 1.04 | (0.87, 1.23) | 0.088 | .665 |
| Language | 1.28 | (0.85, 1.93) | 0.210 | .239 |
| Maternal Age | 0.86 | (0.67, 1.10) | 0.126 | .236 |
| Marital Status | 0.90 | (0.69, 1.16) | 0.132 | .408 |
| Racial Identity | 1.05 | (0.92, 1.21) | 0.069 | .466 |
| Sex | 1.07 | (0.87, 1.30) | 0.102 | .519 |
| Insurance Status | 0.73 | (0.66, 0.82) | 0.057 | <.001 |
| Region | 0.99 | (0.89, 1.11) | 0.054 | .913 |

*Survey-weighted logistic regression estimates of factors associated with Varicella Vaccine, NIS-Child 2012*.

## **Varicella Results 2013**

| Predictor | Odds Ratio | 95% CI | SE | P value |
| --- | --- | --- | --- | --- |
| (Intercept) | 6.44 | (1.04, 39.94) | 0.931 | .045 |
| Age Group | 1.34 | (1.15, 1.55) | 0.077 | <.001 |
| Household size | 0.91 | (0.81, 1.03) | 0.060 | .129 |
| Breastfeeding History | 1.50 | (1.09, 2.06) | 0.161 | .012 |
| WIC benefits | 0.67 | (0.47, 0.94) | 0.175 | .020 |
| Maternal Education | 1.21 | (1.05, 1.40) | 0.072 | .007 |
| Firstborn | 1.16 | (0.85, 1.58) | 0.156 | .340 |
| Hispanic Ethnicity | 0.77 | (0.52, 1.15) | 0.203 | .201 |
| Income | 0.99 | (0.82, 1.20) | 0.097 | .912 |
| Language | 1.86 | (0.99, 3.52) | 0.324 | .055 |
| Maternal Age | 1.27 | (0.95, 1.70) | 0.150 | .109 |
| Marital Status | 0.77 | (0.57, 1.03) | 0.148 | .076 |
| Racial Identity | 1.27 | (1.06, 1.51) | 0.089 | .008 |
| Sex | 0.93 | (0.73, 1.19) | 0.125 | .576 |
| Insurance Status | 0.67 | (0.58, 0.79) | 0.078 | <.001 |
| Region | 0.92 | (0.80, 1.05) | 0.068 | .204 |

*Survey-weighted logistic regression estimates of factors associated with Varicella Vaccine, NIS-Child 2013*.

## **Varicella Results 2014**

| Predictor | Odds Ratio | 95% CI | SE | P value |
| --- | --- | --- | --- | --- |
| (Intercept) | 7.14 | (1.09, 46.66) | 0.957 | .040 |
| Age Group | 1.31 | (1.12, 1.53) | 0.079 | <.001 |
| Household size | 0.90 | (0.82, 0.99) | 0.046 | .026 |
| Breastfeeding History | 1.22 | (0.91, 1.65) | 0.153 | .188 |
| WIC benefits | 0.74 | (0.49, 1.12) | 0.212 | .155 |
| Maternal Education | 1.05 | (0.90, 1.23) | 0.079 | .536 |
| Firstborn | 1.35 | (1.03, 1.77) | 0.139 | .031 |
| Hispanic Ethnicity | 0.70 | (0.46, 1.05) | 0.208 | .082 |
| Income | 1.29 | (1.03, 1.61) | 0.115 | .028 |
| Language | 1.53 | (0.97, 2.41) | 0.234 | .071 |
| Maternal Age | 1.07 | (0.80, 1.43) | 0.148 | .661 |
| Marital Status | 1.46 | (1.05, 2.02) | 0.167 | .024 |
| Racial Identity | 1.14 | (0.95, 1.38) | 0.096 | .168 |
| Sex | 0.99 | (0.78, 1.25) | 0.122 | .907 |
| Insurance Status | 0.71 | (0.61, 0.83) | 0.080 | <.001 |
| Region | 0.86 | (0.76, 0.97) | 0.063 | .014 |

*Survey-weighted logistic regression estimates of factors associated with Varicella Vaccine, NIS-Child 2014*.

## **Varicella Results 2015**

| Predictor | Odds Ratio | 95% CI | SE | P value |
| --- | --- | --- | --- | --- |
| (Intercept) | 14.08 | (3.24, 61.24) | 0.750 | <.001 |
| Age Group | 1.19 | (1.05, 1.36) | 0.066 | .008 |
| Household size | 0.88 | (0.81, 0.96) | 0.045 | .005 |
| Breastfeeding History | 1.18 | (0.88, 1.57) | 0.147 | .266 |
| WIC benefits | 0.68 | (0.49, 0.95) | 0.168 | .022 |
| Maternal Education | 1.23 | (1.07, 1.42) | 0.073 | .004 |
| Firstborn | 1.36 | (1.03, 1.79) | 0.140 | .030 |
| Hispanic Ethnicity | 0.67 | (0.49, 0.92) | 0.159 | .013 |
| Income | 1.06 | (0.89, 1.26) | 0.089 | .503 |
| Language | 1.19 | (0.84, 1.67) | 0.175 | .331 |
| Maternal Age | 1.32 | (1.01, 1.73) | 0.137 | .042 |
| Marital Status | 0.85 | (0.63, 1.14) | 0.152 | .281 |
| Racial Identity | 1.10 | (0.93, 1.30) | 0.085 | .251 |
| Sex | 0.98 | (0.78, 1.23) | 0.115 | .852 |
| Insurance Status | 0.67 | (0.58, 0.77) | 0.072 | <.001 |
| Region | 1.01 | (0.89, 1.13) | 0.061 | .933 |

*Survey-weighted logistic regression estimates of factors associated with Varicella vaccine coverage, NIS-Child 2015*.

## **Varicella Results 2016**

| Predictor | Odds Ratio | 95% CI | SE | P value |
| --- | --- | --- | --- | --- |
| (Intercept) | 4.52 | (0.74, 27.65) | 0.924 | .103 |
| Age Group | 1.18 | (1.01, 1.38) | 0.080 | .040 |
| Household size | 0.95 | (0.86, 1.06) | 0.051 | .363 |
| Breastfeeding History | 1.07 | (0.77, 1.48) | 0.165 | .690 |
| WIC benefits | 0.65 | (0.42, 1.02) | 0.227 | .061 |
| Maternal Education | 1.33 | (1.12, 1.58) | 0.089 | .001 |
| Firstborn | 0.97 | (0.73, 1.29) | 0.145 | .849 |
| Hispanic Ethnicity | 0.95 | (0.66, 1.37) | 0.188 | .779 |
| Income | 1.22 | (0.96, 1.54) | 0.121 | .105 |
| Language | 1.84 | (1.14, 2.98) | 0.246 | .013 |
| Maternal Age | 1.06 | (0.79, 1.41) | 0.146 | .713 |
| Marital Status | 1.35 | (0.98, 1.86) | 0.164 | .067 |
| Racial Identity | 1.06 | (0.88, 1.26) | 0.091 | .543 |
| Sex | 1.02 | (0.79, 1.31) | 0.127 | .892 |
| Insurance Status | 0.73 | (0.61, 0.88) | 0.095 | <.001 |
| Region | 0.82 | (0.72, 0.94) | 0.066 | .003 |

*Survey-weighted logistic regression estimates of factors associated with Varicella vaccine coverage, NIS-Child 2016*.

## **Varicella Results 2017**

| Predictor | Odds Ratio | 95% CI | SE | P value |
| --- | --- | --- | --- | --- |
| (Intercept) | 1.94 | (0.46, 8.12) | 0.730 | .363 |
| Age Group | 1.26 | (1.09, 1.45) | 0.072 | .001 |
| Household size | 0.90 | (0.83, 0.98) | 0.043 | .012 |
| Breastfeeding History | 1.44 | (1.05, 1.99) | 0.163 | .025 |
| WIC benefits | 0.50 | (0.33, 0.75) | 0.210 | <.001 |
| Maternal Education | 1.37 | (1.18, 1.59) | 0.076 | <.001 |
| Firstborn | 1.12 | (0.86, 1.45) | 0.133 | .407 |
| Hispanic Ethnicity | 0.84 | (0.60, 1.16) | 0.167 | .290 |
| Income | 1.38 | (1.14, 1.68) | 0.100 | .001 |
| Language | 1.54 | (1.09, 2.18) | 0.178 | .015 |
| Maternal Age | 1.45 | (1.16, 1.82) | 0.115 | .001 |
| Marital Status | 1.25 | (0.96, 1.62) | 0.133 | .095 |
| Racial Identity | 0.98 | (0.83, 1.16) | 0.086 | .819 |
| Sex | 1.16 | (0.92, 1.45) | 0.115 | .203 |
| Insurance Status | 0.77 | (0.65, 0.91) | 0.087 | .003 |
| Region | 0.98 | (0.88, 1.10) | 0.059 | .771 |

*Survey-weighted logistic regression estimates of factors associated with Varicella vaccine coverage, NIS-Child 2017*

## **Varicella Results 2018**

| Predictor | Odds Ratio | 95% CI | SE | P value |
| --- | --- | --- | --- | --- |
| (Intercept) | 25.10 | (3.57, 176.52) | 0.995 | .001 |
| Age Group | 1.39 | (1.22, 1.59) | 0.066 | <.001 |
| Household size | 0.83 | (0.73, 0.93) | 0.060 | .001 |
| Breastfeeding History | 1.13 | (0.82, 1.56) | 0.165 | .453 |
| WIC benefits | 0.67 | (0.48, 0.93) | 0.168 | .016 |
| Maternal Education | 1.11 | (0.95, 1.29) | 0.077 | .187 |
| Firstborn | 1.02 | (0.76, 1.39) | 0.155 | .878 |
| Hispanic Ethnicity | 0.70 | (0.50, 1.00) | 0.178 | .048 |
| Income | 1.09 | (0.91, 1.32) | 0.094 | .337 |
| Language | 1.69 | (1.11, 2.56) | 0.212 | .014 |
| Maternal Age | 1.25 | (0.94, 1.66) | 0.144 | .119 |
| Marital Status | 0.85 | (0.62, 1.15) | 0.156 | .287 |
| Racial Identity | 1.01 | (0.87, 1.18) | 0.079 | .863 |
| Sex | 1.17 | (0.93, 1.47) | 0.116 | .179 |
| Insurance Status | 0.65 | (0.57, 0.73) | 0.064 | <.001 |
| Region | 0.97 | (0.85, 1.11) | 0.069 | .694 |

*Survey-weighted logistic regression estimates of factors associated with Varicella vaccine coverage, NIS-Child 2018*.

## **Varicella Results 2019**

| Predictor | Odds Ratio | 95% CI | SE | P value |
| --- | --- | --- | --- | --- |
| (Intercept) | 3.28 | (0.47, 22.77) | 0.989 | .230 |
| Age Group | 1.12 | (0.94, 1.32) | 0.086 | .204 |
| Household size | 0.90 | (0.79, 1.03) | 0.066 | .117 |
| Breastfeeding History | 2.09 | (1.45, 3.01) | 0.186 | <.001 |
| WIC benefits | 0.69 | (0.47, 1.03) | 0.203 | .071 |
| Maternal Education | 1.28 | (1.05, 1.57) | 0.102 | .014 |
| Firstborn | 1.00 | (0.69, 1.47) | 0.195 | .984 |
| Hispanic Ethnicity | 1.11 | (0.72, 1.73) | 0.225 | .630 |
| Income | 1.26 | (1.02, 1.56) | 0.108 | .030 |
| Language | 2.00 | (1.25, 3.18) | 0.237 | .004 |
| Maternal Age | 1.22 | (0.89, 1.67) | 0.160 | .209 |
| Marital Status | 1.03 | (0.72, 1.48) | 0.184 | .870 |
| Racial Identity | 0.71 | (0.58, 0.88) | 0.107 | .001 |
| Sex | 0.83 | (0.63, 1.10) | 0.142 | .198 |
| Insurance Status | 0.83 | (0.68, 1.01) | 0.100 | .056 |
| Region | 0.93 | (0.80, 1.09) | 0.078 | .371 |

*Survey-weighted logistic regression estimates of factors associated with Varicella vaccine coverage, NIS-Child 2019*.

## **Varicella Results 2020**

| Predictor | Odds Ratio | 95% CI | SE | P value |
| --- | --- | --- | --- | --- |
| (Intercept) | 4.45 | (0.77, 25.92) | 0.898 | .096 |
| Age Group | 1.31 | (1.14, 1.51) | 0.070 | <.001 |
| Household size | 0.89 | (0.81, 0.97) | 0.045 | .009 |
| Breastfeeding History | 1.07 | (0.81, 1.42) | 0.143 | .626 |
| WIC benefits | 0.91 | (0.69, 1.21) | 0.145 | .533 |
| Maternal Education | 1.08 | (0.94, 1.24) | 0.072 | .307 |
| Firstborn | 1.17 | (0.86, 1.59) | 0.158 | .328 |
| Hispanic Ethnicity | 1.12 | (0.84, 1.51) | 0.149 | .430 |
| Income | 1.21 | (1.02, 1.43) | 0.087 | .031 |
| Language | 1.78 | (1.16, 2.74) | 0.220 | .009 |
| Maternal Age | 1.25 | (0.97, 1.60) | 0.128 | .085 |
| Marital Status | 1.05 | (0.80, 1.36) | 0.134 | .741 |
| Racial Identity | 0.92 | (0.81, 1.05) | 0.064 | .207 |
| Sex | 1.03 | (0.84, 1.28) | 0.108 | .767 |
| Insurance Status | 0.69 | (0.59, 0.81) | 0.079 | <.001 |
| Region | 0.96 | (0.87, 1.06) | 0.052 | .426 |

*Survey-weighted logistic regression estimates of factors associated with Varicella vaccine coverage, NIS-Child 2020*.

## **Varicella Results 2021**

| Predictor | Odds Ratio | 95% CI | SE | P value |
| --- | --- | --- | --- | --- |
| (Intercept) | 12.63 | (1.79, 89.17) | 0.997 | .011 |
| Age Group | 1.35 | (1.15, 1.57) | 0.080 | <.001 |
| Household size | 0.92 | (0.83, 1.03) | 0.057 | .168 |
| Breastfeeding History | 1.01 | (0.68, 1.51) | 0.204 | .949 |
| WIC benefits | 0.77 | (0.57, 1.05) | 0.157 | .097 |
| Maternal Education | 1.13 | (0.97, 1.32) | 0.079 | .122 |
| Firstborn | 1.19 | (0.87, 1.62) | 0.159 | .279 |
| Hispanic Ethnicity | 1.00 | (0.71, 1.39) | 0.171 | .984 |
| Income | 1.15 | (0.95, 1.38) | 0.094 | .143 |
| Language | 1.17 | (0.64, 2.16) | 0.311 | .607 |
| Maternal Age | 1.40 | (1.06, 1.85) | 0.142 | .019 |
| Marital Status | 0.75 | (0.56, 1.01) | 0.152 | .062 |
| Racial Identity | 0.90 | (0.77, 1.05) | 0.077 | .166 |
| Sex | 1.03 | (0.80, 1.32) | 0.126 | .813 |
| Insurance Status | 0.72 | (0.58, 0.89) | 0.112 | .003 |
| Region | 0.86 | (0.76, 0.98) | 0.065 | .025 |

*Survey-weighted logistic regression estimates of factors associated with Varicella vaccine coverage, NIS-Child 2021*.

## **Varicella Results 2022**

| Predictor | Odds Ratio | 95% CI | SE | P value |
| --- | --- | --- | --- | --- |
| (Intercept) | 28.12 | (3.66, 215.79) | 1.040 | .001 |
| Age Group | 1.26 | (1.09, 1.47) | 0.076 | .002 |
| Household size | 0.87 | (0.79, 0.96) | 0.049 | .005 |
| Breastfeeding History | 0.85 | (0.58, 1.25) | 0.194 | .418 |
| WIC benefits | 0.76 | (0.53, 1.08) | 0.185 | .129 |
| Maternal Education | 1.24 | (1.08, 1.43) | 0.072 | .002 |
| Firstborn | 0.98 | (0.72, 1.32) | 0.152 | .875 |
| Hispanic Ethnicity | 0.77 | (0.56, 1.06) | 0.164 | .114 |
| Income | 1.03 | (0.80, 1.33) | 0.128 | .812 |
| Language | 1.29 | (0.83, 2.01) | 0.228 | .265 |
| Maternal Age | 1.12 | (0.84, 1.50) | 0.148 | .428 |
| Marital Status | 0.99 | (0.70, 1.40) | 0.175 | .960 |
| Racial Identity | 1.01 | (0.87, 1.18) | 0.078 | .890 |
| Sex | 1.07 | (0.84, 1.36) | 0.122 | .570 |
| Insurance Status | 0.68 | (0.58, 0.80) | 0.081 | <.001 |
| Region | 0.97 | (0.86, 1.10) | 0.061 | .637 |

*Survey-weighted logistic regression estimates of factors associated with Varicella vaccine coverage, NIS-Child 2022*.

## **Varicella Vaccine Results 2023**

| Predictor | Odds Ratio | 95% CI | SE | P value |
| --- | --- | --- | --- | --- |
| (Intercept) | 76.03 | (15.66, 369.15) | 0.806 | <.001 |
| Age Group | 1.16 | (1.00, 1.34) | 0.074 | .050 |
| Household size | 0.84 | (0.74, 0.95) | 0.062 | .004 |
| Breastfeeding History | 0.77 | (0.55, 1.08) | 0.170 | .131 |
| WIC benefits | 0.77 | (0.58, 1.02) | 0.143 | .072 |
| Maternal Education | 1.20 | (1.06, 1.37) | 0.067 | .006 |
| Firstborn | 0.83 | (0.64, 1.07) | 0.132 | .156 |
| Hispanic Ethnicity | 0.80 | (0.59, 1.09) | 0.158 | .161 |
| Income | 1.07 | (0.89, 1.30) | 0.096 | .465 |
| Language | 1.38 | (0.81, 2.34) | 0.270 | .237 |
| Maternal Age | 1.16 | (0.88, 1.52) | 0.139 | .293 |
| Marital Status | 0.88 | (0.68, 1.14) | 0.129 | .328 |
| Racial Identity | 1.01 | (0.89, 1.16) | 0.069 | .840 |
| Sex | 1.02 | (0.82, 1.27) | 0.113 | .862 |
| Insurance Status | 0.67 | (0.59, 0.77) | 0.066 | <.001 |
| Region | 0.82 | (0.73, 0.93) | 0.060 | .001 |

*Survey-weighted logistic regression estimates of factors associated with Varicella vaccine coverage, NIS-Child 2023*.
